# Supplementary material for: Synthesis and Properties of (1R(S),5R(S),7R(S),8R(S))-1,8-Bis(hydroxymethyl)-6-azadispiro[4.1.4.2]tridecane-6-oxyl: Reduction-Resistant Spin Labels with High Spin Relaxation Times
Source: Int J Mol Sci. 2023 Jul 15;24(14):11498. doi: 10.3390/ijms241411498 (PMC10380268; doi:10.3390/ijms241411498)
Supplement: Supplementary file 1 [file ijms-24-11498-s001.zip › SupportEaredFin-5.pdf]

International Journal of
Molecular Sciences

MDPI

1. Table of Contents.

1. Table of Contents....1

2. IR spectral data. 5

Fig. S1. IR spectrum of (1*R*(*S*),5*R*(*S*),7*R*(*S*),8*R*(*S*))-1,8-Bis(hydroxymethyl)-6-azadispiro[4.1.4.2]tridecane 6-oxyl (**5**) (KBr)....5

Fig. S2. IR spectrum of [(5*R*(*S*),6*R*(*S*))-1-Oxido-1-azaspiro[4.4]non-1-en-6-yl]methanol (**9**) (KBr)....5

Fig. S3. IR spectrum of [(5*R*(*S*),6*R*(*S*))-1-Oxido-2-pent-4-en-1-yl-1-azaspiro[4.4]non-1-en-6-yl]methanol (**12**) (neat)....6

Fig. S4. IR spectrum of (1*R*(*S*),2*R*(*S*),6a′*R*(*S*),9a′*R*(*S*))-Hexahydro-6′*H*-spiro[cyclopentan-1,3′-cyclopenta[*c*]pyrrolo[1,2-*b*]isoxazol]-2-ylmethanol (**13**) (neat)....6

Fig. S5. IR spectrum of (1*R*(*S*),5*R*(*S*),7*R*(*S*),8*R*(*S*))-6-azadispiro[4.1.4.2]tridecan-1,8-diyldimethanol (**14**) (KBr)....7

Fig. S6. IR spectrum of (1*R*(*S*),5*R*(*S*),7*R*(*S*),8*R*(*S*))-6-azadispiro[4.1.4.2]tridecan-1,8-diyldi(methylene) diacetate (**16**) (neat)....7

Fig. S7. IR spectrum of 1*R*(*S*),5*R*(*S*),7*R*(*S*),8*R*(*S*))-1,8-Bis[(acetyloxy)methyl]-6-azadispiro[4.1.4.2]tridecane-6-oxyl (**17**) (KBr)....8

Fig. S8. IR spectrum of (4*R*(*S*),5*S*(*R*),7*R*(*S*),8*R*(*S*))-4,8-Bis[(acetyloxy)methyl]-6-azadispiro[4.1.4.2]tridec-1-ene-6-oxyl (**18**) (neat)....8

Fig. S9. IR spectrum of. (1*R*(*S*),5*R*(*S*),7*R*(*S*),8*R*(*S*))-6-Azadispiro[4.1.4.2]tridecan-1,8-dicarboxylic acid **20** (KBr)....9

Fig. S10. IR spectrum of Dimethyl (1*R*(*S*),5*R*(*S*),7*R*(*S*),8*R*(*S*))-6-azadispiro[4.1.4.2]tridecan-1,8-dicarboxylate (**21**) (neat)....9

Fig. S11. IR spectrum of (1*R*(*S*),5*R*(*S*),7*R*(*S*),8*R*(*S*))-1,8-Bis(methoxycarbonyl)-6-azadispiro[4.1.4.2]tridecane-6-oxyl (**22**) (KBr)....10

Fig. S12. IR spectrum of 1*R*(*S*),5*R*(*S*),7*R*(*S*),8*R*(*S*))-1,8-Dicarboxy-6-azadispiro[4.1.4.2]tridecane-6-oxyl (**23a**) (KBr)....10

Fig. S13. IR spectrum of (1*R*(*S*),5*R*(*S*),7*R*(*S*),8*R*(*S*))-1,8-Bis[({1*H*-imidazol-1-ylcarbonyl}oxy)methyl]-6-azadispiro[4.1.4.2]tridecane-6-oxyl (**26**) (KBr)....11

Fig. S14. IR spectrum of (1*R*(*S*),5*R*(*S*),7*R*(*S*),8*R*(*S*))-1,8-Bis[(([{3-(dimethylamino)propyl}amino]carbonyl)oxy)methyl]-6-azadispiro[4.1.4.2]tridecane-6-oxyl (**27**) (neat)....11

Fig. S15. IR spectrum of 1*R*(*S*),5*R*(*S*),7*R*(*S*),8*R*(*S*)-1,8-Bis[(([{3-methoxy-3-oxopropyl}amino]carbonyl)oxy)methyl]-6-azadispiro[4.1.4.2]tridecane-6-oxyl (**28**) (neat)....12

Fig. SI16. IR spectrum of 1*R*(*S*),5*R*(*S*),7*R*(*S*),8*R*(*S*)-1,8-Bis[(([{2-carboxyethyl}amino]carbonyl)oxy)methyl]-6-azadispiro[4.1.4.2]tridecane-6-oxyl (**29**) (KBr)....12

Fig. S17. IR spectrum of (1*R*(*S*),5*R*(*S*),7*R*(*S*),8*R*(*S*))-1,8-Bis[(prop-2-yn-1-yloxy)methyl]-6-azadispiro[4.1.4.2]tridecane-6-oxyl (**30**) (neat)....13

Fig. S18. IR spectrum of (1*R*(*S*),5*R*(*S*),7*R*(*S*),8*R*(*S*))-1-(Hydroxymethyl)-8-[(prop-2-yn-1-yloxy)methyl]-6-azadispiro[4.1.4.2]tridecane-6-oxyl (**31**) (neat)....13

Fig. S19. IR spectrum of (1*R*(*S*),5*R*(*S*),7*R*(*S*),8*R*(*S*))-1-(Hydroxymethyl-8-({(1-[2,3,4,6-tetra-*O*-acetyl-β-D-galactopyranosyl]-1*H*-1,2,3-triazol-4-yl)methoxy}methyl)-6-azadispiro[4.1.4.2]tridecane-6-oxyl (**32**) (KBr) 14

Fig. S20. IR spectrum of (1*R*(*S*),5*R*(*S*),7*R*(*S*),8*R*(*S*))-1-(Hydroxymethyl)-8-({(1-[β-D-galactopyranosyl]-1*H*-1,2,3-triazol-4-yl)methoxy}methyl)-6-azadispiro[4.1.4.2]tridecane-6-oxyl (**33**) (KBr)....14

Fig. S21. IR spectrum of 2,2,5,5-Tetramethyl-3-(((1-((β-D-galactopyranosyl)-1*H*-1,2,3-triazol-4-yl)methoxy)methyl)pyrrolidine-1-oxyl (**35**) (KBr)....15

Fig. S22. IR spectrum of 2,2,5,5-Tetramethyl-3-((prop-2-ynyloxy)methyl)pyrrolidine-1-oxyl (**37**) (KBr)....15

Fig. S23. IR spectrum of 2,2,5,5-Tetramethyl-3-(((1-(2,3,4,6-tetra-*O*-acetyl-β-D-galactopyranosyl)-1*H*-1,2,3-triazol-4-yl)methoxy)methyl)pyrrolidine-1-oxyl (**38**) (KBr)....16

3. UV spectral data....17

Fig. S24. UV spectrum of [(5*R*(*S*),6*R*(*S*))-1-Oxido-1-azaspiro[4.4]non-1-en-6-yl]methanol (**9**)....17

Fig. S25. UV spectrum of [(5*R*(*S*),6*R*(*S*))-1-Oxido-2-pent-4-en-1-yl-1-azaspiro[4.4]non-1-en-6-yl]methanol (**12**)....17

Fig. S26. UV spectrum of (1*R*(*S*),5*S*(*R*),7*R*(*S*),8*R*(*S*))-1,8-Bis[(acetyloxy)methyl]-6-azadispiro[4.1.4.2]tridecane-6-oxyl (**17**)....18

Fig. S27. UV spectrum of (1*R*(*S*),5*R*(*S*),7*R*(*S*),8*R*(*S*))-1,8-Bis[Methoxycarbonyl]-6-azadispiro[4.1.4.2]tridecane-6-oxyl (**22**)....18

Fig. S28. UV spectrum of 1*R*(*S*),5*R*(*S*),7*R*(*S*),8*R*(*S*))-1,8-Dicarboxy-6-azadispiro[4.1.4.2]tridecane-6-oxyl (**23a**) in EtOH....19

Fig. S29. UV spectrum of (1*R*(*S*),5*R*(*S*),7*R*(*S*),8*R*(*S*))-1,8-Bis[({1*H*-imidazol-1-ylcarbonyl}oxy)methyl]-6-azadispiro[4.1.4.2]tridecane-6-oxyl (**26**)....19

Fig. S30. UV spectrum of (1*R*(*S*),5*R*(*S*),7*R*(*S*),8*R*(*S*))-1-(Hydroxymethyl)-8-[(prop-2-yn-1-yloxy)methyl]-6-azadispiro[4.1.4.2]tridecane-6-oxyl (**31**)....20

4. ^{1}H and ^{13}C NMR spectral data....21

Fig. S31. ^{1}H NMR spectrum of [(5*R*(*S*),6*R*(*S*))-1-Oxido-1-azaspiro[4.4]non-1-en-6-yl]methanol (**9**) in $CDCl_3$ at 400 MHz....21

Fig. S32. ^{13}C NMR spectrum of [(5*R*(*S*),6*R*(*S*))-1-Oxido-1-azaspiro[4.4]non-1-en-6-yl]methanol (**9**) in $CDCl_3$ at 125 MHz....21

Fig. S33. ^{1}H NMR spectrum of [(5*R*(*S*),6*R*(*S*))-1-Oxido-2-(pent-4-en-1-yl)-1-azaspiro[4.4]non-1-en-6-yl]methanol (**12**) in $CDCl_3$ at 400 MHz....22

Fig. S34. ^{13}C NMR spectrum of [(5*R*(*S*),6*R*(*S*))-1-Oxido-2-(pent-4-en-1-yl)-1-azaspiro[4.4]non-1-en-6-yl]methanol (**12**) in $CDCl_3$ at 100 MHz....22

Fig. S35. ^{1}H NMR spectrum of (1*R*(*S*),2*R*(*S*),6a′*R*(*S*),9a′*R*(*S*)-Hexahydro-6′*H*-spiro[cyclopentan-1,3′-cyclopenta[*c*]pyrrolo[1,2-*b*]isoxazole]-2-ylmethanol (**13**) in $CDCl_3$ at 600 MHz....23

Fig. S36. ^{13}C NMR spectrum of (1*R*(*S*),2*R*(*S*),6a′*R*(*S*),9a′*R*(*S*)-Hexahydro-6′*H*-spiro[cyclopentan-1,3′-cyclopenta[*c*]pyrrolo[1,2-*b*]isoxazole]-2-ylmethanol (**13**) in $CDCl_3$ at 150 MHz....23

Fig. S37. ^{1}H NMR spectrum of (1*R*(*S*),5*R*(*S*),7*R*(*S*),8*R*(*S*))-6-azadispiro[4.1.4.2]tridecan-1,8-diyldimethanol (**14**) in $CDCl_3$ at 400 MHz....24

Fig. S38. ^{13}C NMR spectrum of (1*R*(*S*),5*R*(*S*),7*R*(*S*),8*R*(*S*))-6-azadispiro[4.1.4.2]tridecan-1,8-diyldimethanol (**14**) in $CDCl_3$ at 100 MHz....24

Fig. S39. ^{1}H NMR spectrum of (1*R*(*S*),5*R*(*S*),7*R*(*S*),8*R*(*S*))-6-azadispiro[4.1.4.2]tridecan-1,8-diyldi(methylene) diacetate (**16**) in $CDCl_3$ at 500 MHz....25

Fig. S40. ^{13}C NMR spectrum of (1*R*(*S*),5*R*(*S*),7*R*(*S*),8*R*(*S*))-6-azadispiro[4.1.4.2]tridecan-1,8-diyldi(methylene) diacetate (**16**) in $CDCl_3$ at 125 MHz....25

Fig. S41. ^{1}H NMR spectrum of (4*R*(*S*),5*S*(*R*),7*R*(*S*),8*R*(*S*))-4,8-Bis[(acetyloxy)methyl]-6-azoniadispiro[4.1.4.2]tridec-1-ene trifluoroacetate (**19**) in CD_3OD+CF_3COOH at 400 MHz....26

Fig. S42. ^{13}C NMR spectrum of (4*R*(*S*),5*S*(*R*),7*R*(*S*),8*R*(*S*))-4,8-Bis[(acetyloxy)methyl]-6-azoniadispiro[4.1.4.2]tridec-1-ene trifluoroacetate (**19**) in CD_3OD+CF_3COOH at 100 MHz 26

Fig. S43. ^{1}H NMR spectrum of (1*R*(*S*),5*R*(*S*),7*R*(*S*),8*R*(*S*))-6-Azadisporo[4.1.4.2]tridecan-1,8-dicarboxylic acid (**20**) in CD_3OD at 400 MHz 27

Fig. S44. ^{1}H NMR spectrum of (1*R*(*S*),5*R*(*S*),7*R*(*S*),8*R*(*S*))-6-Azadisporo[4.1.4.2]tridecan-1,8-dicarboxylic acid (**20**) in CD_3OD at 75 MHz 27

Fig. S45. ^{1}H NMR spectrum of Dimethyl (1*R*(*S*),5*R*(*S*),7*R*(*S*),8*R*(*S*))-6-azadispiro[4.1.4.2]tridecan-1,8-dicarboxylate (**21**) in $CDCl_3$ at 300 MHz 28

Fig. S46. ^{1}H NMR spectrum of Dimethyl (1*R*(*S*),5*R*(*S*),7*R*(*S*),8*R*(*S*))-6-azadispiro[4.1.4.2]tridecan-1,8-dicarboxylate (**21**) in $CDCl_3$ at 75 MHz 28

Fig. S47. ^{1}H NMR spectrum of (1*R*(*S*),5*R*(*S*),7*R*(*S*),8*R*(*S*))-1,8-Bis(methoxycarbonyl)-6-azoniadispiro[4.1.4.2]tridecane trifluoroacetate (**22-red**) in CD_3OD+CF_3COOH at 300 MHz 29

Fig. S48. ^{1}H NMR spectrum of (1*R*(*S*),5*R*(*S*),7*R*(*S*),8*R*(*S*))-1,8-Dicarboxy-6-azoniadispiro[4.1.4.2]tridecane trifluoroacetate (**23a-red**) in CD_3OD+CF_3COOH at 500 MHz 29

Fig. S49. ^{13}C NMR spectrum of (1*R*(*S*),5*R*(*S*),7*R*(*S*),8*R*(*S*))-1,8-Dicarboxy-6-azoniadispiro[4.1.4.2]tridecane trifluoroacetate (**23a-red**) in CD_3OD+CF_3COOH at 125 MHz 30

Fig. S50. ^{1}H NMR spectrum of isomeric mixture of 1,8-Dicarboxy-6-azoniadispiro[4.1.4.2]tridecane trifluoroacetates (**23a+23b+23c-red**) in CD_3OD+CF_3COOH at 500 MHz 30

Fig. S51. ^{13}C NMR spectrum of isomeric mixture of 1,8-Dicarboxy-6-azoniadispiro[4.1.4.2]tridecane trifluoroacetates (**23a+23b+23c-red**) in CD_3OD+CF_3COOH at 125 MHz 31

Fig. S52. ^{1}H NMR spectrum of (1R(S),5R(S),7R(S),8R(S))-1,8-Bis[((([{3-(dimethylamino)propyl}amino]carbonyl)oxy)methyl]-6-azoniadispiro[4.1.4.2]tridecane trifluoroacetate (**27-red**) in CD_3OD+CF_3COOD at 500 MHz 31

Fig. S53. ^{13}C NMR spectrum of (1R(S),5R(S),7R(S),8R(S))-1,8-Bis[((([{3-(dimethylamino)propyl}amino]carbonyl)oxy)methyl]-6-azoniadispiro[4.1.4.2]tridecane trifluoroacetate (**27-red**) in CD_3OD+CF_3COOD at 125 MHz 32

Fig. S54. ^{1}H NMR spectrum of (1*R*(*S*),5*R*(*S*),7*R*(*S*),8*R*(*S*))-1,8-Bis[((([{3-methoxy-3-oxopropyl}amino]carbonyl)oxy)methyl]-6-azoniadispiro[4.1.4.2]tridecane trifluoroacetate (**28-red**) in CD_3OD+CF_3COOD at 500 MHz 32

Fig. S55. ^{13}C NMR spectrum of (1*R*(*S*),5*R*(*S*),7*R*(*S*),8*R*(*S*))-1,8-Bis[((([{3-methoxy-3-oxopropyl}amino]carbonyl)oxy)methyl]-6-azoniadispiro[4.1.4.2]tridecane trifluoroacetate (**28-red**) in CD_3OD+CF_3COOD at 125 MHz 33

Fig. S56. ^{1}H NMR spectrum of (1*R*(*S*),5*R*(*S*),7*R*(*S*),8*R*(*S*))-1,8-Bis[((([{2-carboxyethyl}amino]carbonyl)oxy)methyl]-6-azoniadispiro[4.1.4.2]tridecane trifluoroacetate (**29-red**) in CD_3OD+CF_3COOD at 400 MHz 33

Fig. S57. ^{13}C NMR spectrum of (1*R*(*S*),5*R*(*S*),7*R*(*S*),8*R*(*S*))-1,8-Bis[((([{2-carboxyethyl}amino]carbonyl)oxy)methyl]-6-azoniadispiro[4.1.4.2]tridecane trifluoroacetate (**29-red**) in CD_3OD+CF_3COOD at 150 MHz 34

Fig. S58. ^{1}H NMR spectrum of (1*R*(*S*),5*R*(*S*),7*R*(*S*),8*R*(*S*))-1-(Hydroxymethyl)-8-({(1-[2,3,4,6-tetra-*O*-acetyl-β-D-galactopyranosyl]-1*H*-1,2,3-triazol-4-yl)methoxy}methyl)-6-azoniadispiro[4.1.4.2]tridecane trifluoroacetate (**32-red**) in CD_3OD+CF_3COOH at 400 MHz 34

Fig. S59. ^{13}C NMR spectrum of (1*R*(*S*),5*R*(*S*),7*R*(*S*),8*R*(*S*))-1-(Hydroxymethyl)-8-({(1-[2,3,4,6-tetra-*O*-acetyl-β-D-galactopyranosyl]-1*H*-1,2,3-triazol-4-yl)methoxy}methyl)-6-azoniadispiro[4.1.4.2]tridecane trifluoroacetate (**32-red**) in CD_3OD+CF_3COOD at 100 MHz 35

Fig. S60. ^{1}H NMR spectrum of (1*R*(*S*),5*R*(*S*),7*R*(*S*),8*R*(*S*))-1-(Hydroxymethyl)-8-({(1-[β-D-galactopyranosyl]-1*H*-1,2,3-triazol-4-yl)methoxy}methyl)-6-azoniadispiro[4.1.4.2]tridecane trifluoroacetate (**33-red**) in CD_3OD+HCOOH+HOOCCOOH at 500 MHz 35

Fig. S61. ^{13}C NMR spectrum of (1*R*(*S*),5*R*(*S*),7*R*(*S*),8*R*(*S*))-1-(Hydroxymethyl)-8-({(1-[β-D-galactopyranosyl]-1*H*-1,2,3-triazol-4-yl)methoxy}methyl)-6-azoniadispiro[4.1.4.2]tridecane trifluoroacetate (**33-red**) in CD_3OD+HCOOH+HOOCCOOH at 125 MHz 36

Fig. S62. ^{1}H NMR spectrum of 2,2,5,5-Tetramethyl-3-(((1-(2,3,4,6-tetra-*O*-acetyl-β-D-galactopyranosyl)-1*H*-1,2,3-triazol-4-yl)methoxy)methyl)pyrrolidinium trifluoroacetate (**38-red**) in CD_3OD+CF_3COOH at 300 MHz 36

Fig. S63. ^{1}H NMR spectrum of 1-Hydroxy-2,2,5,5-tetramethyl-3-(((1-((β-D-galactopyranosyl)-1*H*-1,2,3-triazol-4-yl)methoxy)methyl)pyrrolidinium trifluoroacetate (**35-red**) in CD_3OD+CF_3COOH at 500 MHz 37

5. 2D NMR spectral data 38

Fig. S64. ^{1}H-^{1}H COSY NMR spectrum of (1*R*(*S*),2*R*(*S*),6a′*R*(*S*),9a′*R*(*S*)-Hexahydro-6′*H*-spiro[cyclopentan-1,3′-cyclopenta[*c*]pyrrolo[1,2-*b*]isoxazol]-2-ylmethanol (**13**) in $CDCl_3$. 38

Fig. S65. ^{1}H-^{13}C HMBC NMR spectrum of (1*R*(*S*),2*R*(*S*),6a′*R*(*S*),9a′*R*(*S*)-Hexahydro-6′*H*-spiro[cyclopentan-1,3′-cyclopenta[*c*]pyrrolo[1,2-*b*]isoxazol]-2-ylmethanol (**13**) in $CDCl_3$. 38

Fig. S66. ^{1}H-^{13}C HSQC NMR spectrum of (1*R*(*S*),2*R*(*S*),6a′*R*(*S*),9a′*R*(*S*)-Hexahydro-6′*H*-spiro[cyclopentan-1,3′-cyclopenta[*c*]pyrrolo[1,2-*b*]isoxazol]-2-ylmethanol (**13**) in $CDCl_3$. 39

Fig. S67. ^{1}H-^{1}H NOESY NMR spectrum of (1*R*(*S*),2*R*(*S*),6a′*R*(*S*),9a′*R*(*S*)-Hexahydro-6′*H*-spiro[cyclopentan-1,3′-cyclopenta[*c*]pyrrolo[1,2-*b*]isoxazol]-2-ylmethanol (**13**) in $CDCl_3$. 39

Fig. S68. ^{1}H-^{1}H COSY NMR spectrum of (4*R*(*S*),5*S*(*R*),7*R*(*S*),8*R*(*S*))-4,8-Bis[(acetyloxy)methyl]-6-azoniadispiro[4.1.4.2]tridec-1-ene trifluoroacetate (**19**) in CD_3OD+CF_3COOH. 40

Fig. S69. ^{1}H-^{13}C HSQC NMR spectrum of (4*R*(*S*),5*S*(*R*),7*R*(*S*),8*R*(*S*))-4,8-Bis[(acetyloxy)methyl]-6-azoniadispiro[4.1.4.2]tridec-1-ene trifluoroacetate (**19**) in CD_3OD+CF_3COOH. 40

Fig. S70. ^{1}H-^{13}C HMBC NMR spectrum of (4*R*(*S*),5*S*(*R*),7*R*(*S*),8*R*(*S*))-4,8-Bis[(acetyloxy)methyl]-6-azoniadispiro[4.1.4.2]tridec-1-ene trifluoroacetate (**19**) in CD_3OD+CF_3COOH. 41

6. EPR measurements. 42

Fig. S71. The kinetics of decrease of EPR signal of nitroxides **5**, **29** and **33** in solution of ascorbic acid (100mM) and glutathione (5mM). 42

Fig. S72. The dependence of first order reaction rate constants versus ascorbic acid concentration of the radicals **5**, **29** and **33**. 42

Fig. S73. Data for calculation of partition coefficients of the radicals **5**, **29** and 33. 43

Table S1. Experimental details of X-ray diffraction experiments for compounds investigated. 44

International Journal of
Molecular Sciences

MDPI

2. IR spectral data.

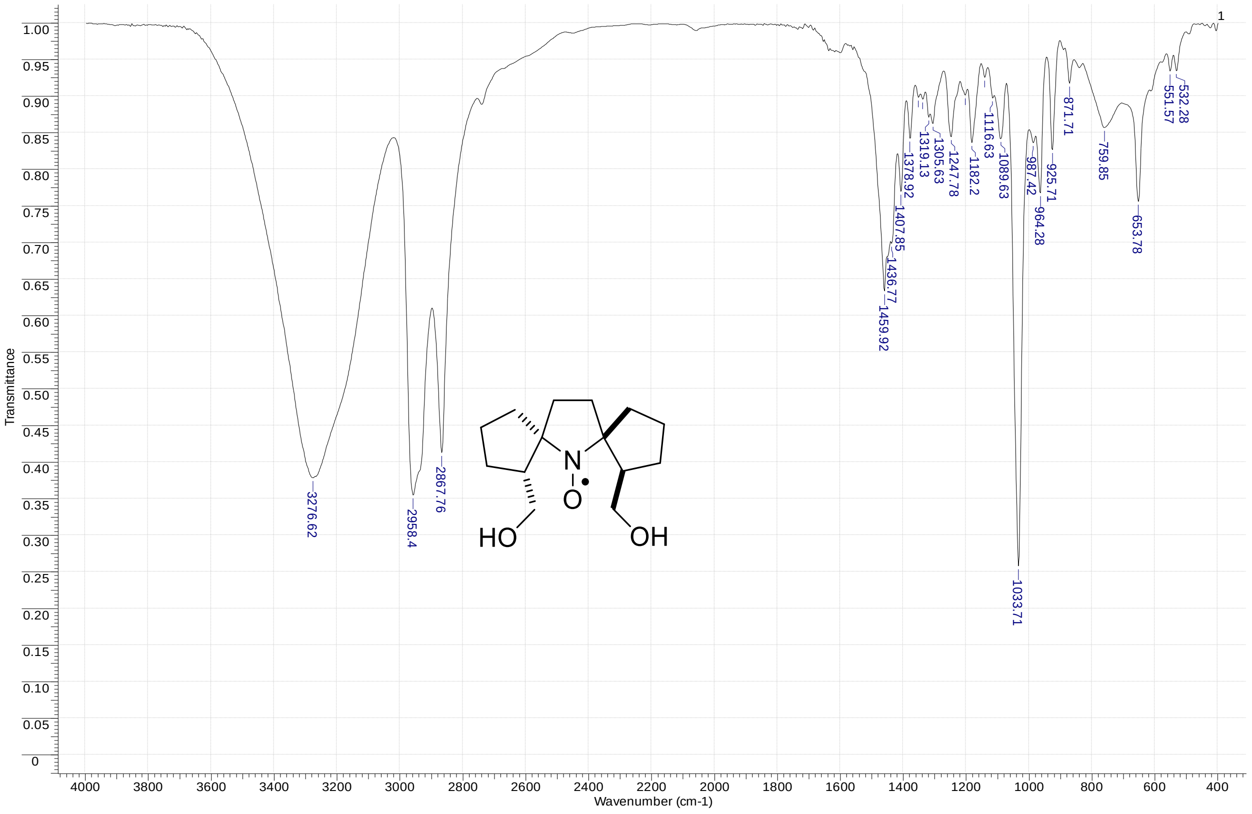

Fig. S1. IR spectrum of (1*R*(*S*),5*R*(*S*),7*R*(*S*),8*R*(*S*))-1,8-Bis(hydroxymethyl)-6-azadispiro[4.1.4.2]tridecane 6-oxyl (**5**) (KBr)

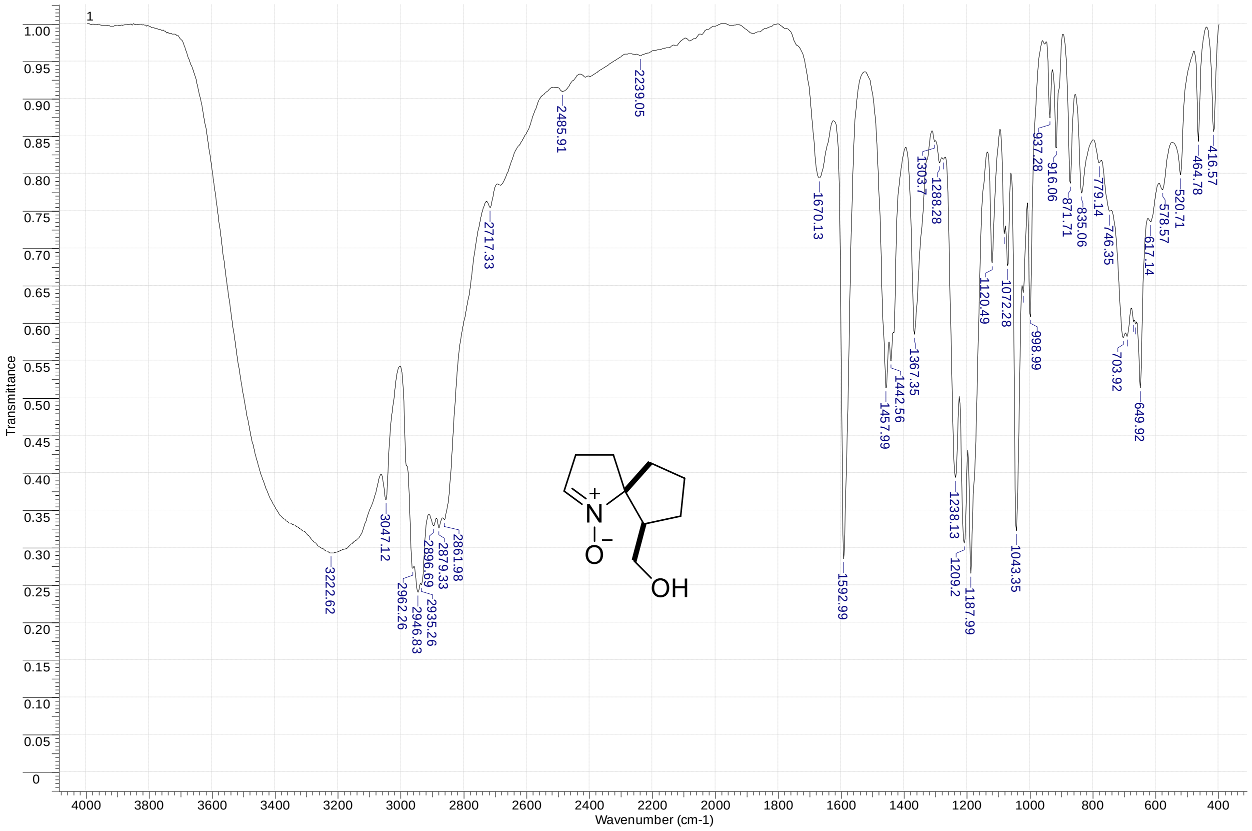

Fig. S2. IR spectrum of [(5*R*(*S*),6*R*(*S*))-1-Oxido-1-azaspiro[4.4]non-1-en-6-yl]methanol (**9**) (KBr)

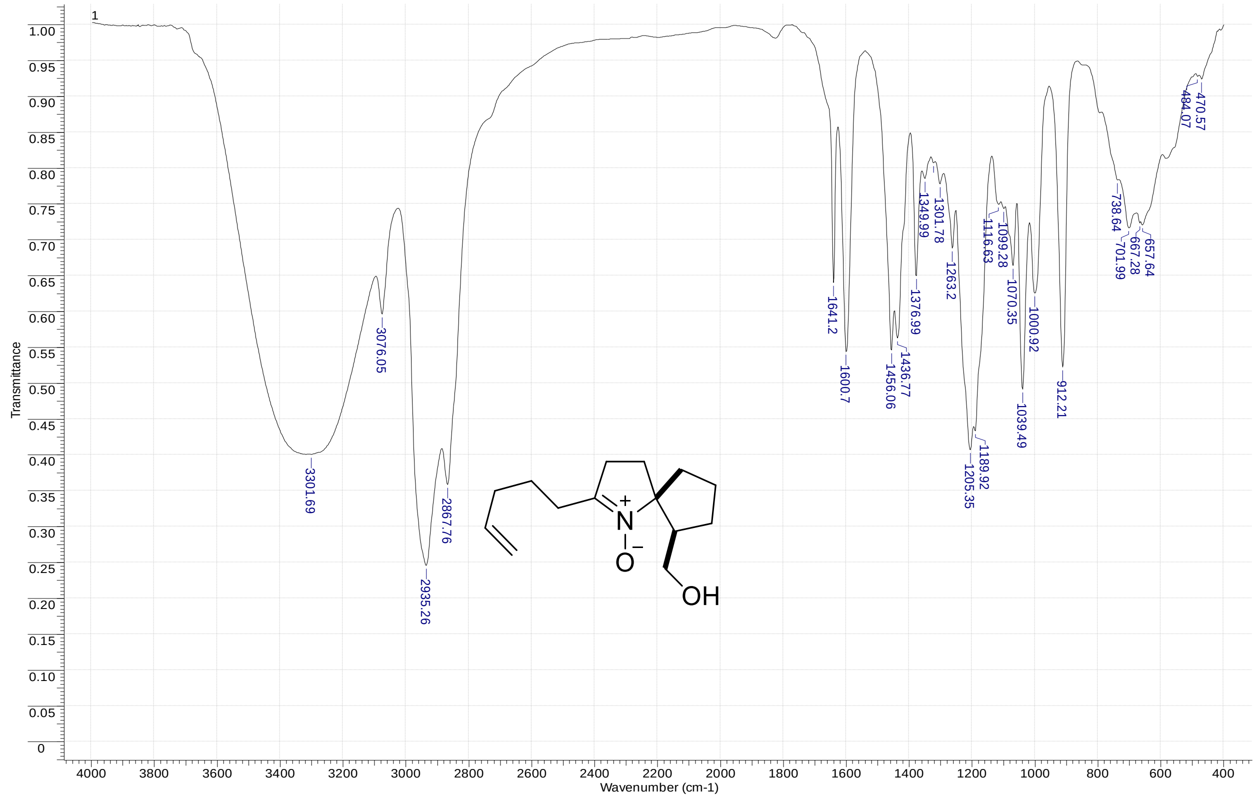

Fig. S3. IR spectrum of [(5*R*(*S*),6*R*(*S*))-1-Oxido-2-pent-4-en-1-yl-1-azaspiro[4.4]non-1-en-6-yl]methanol (**12**) (neat)

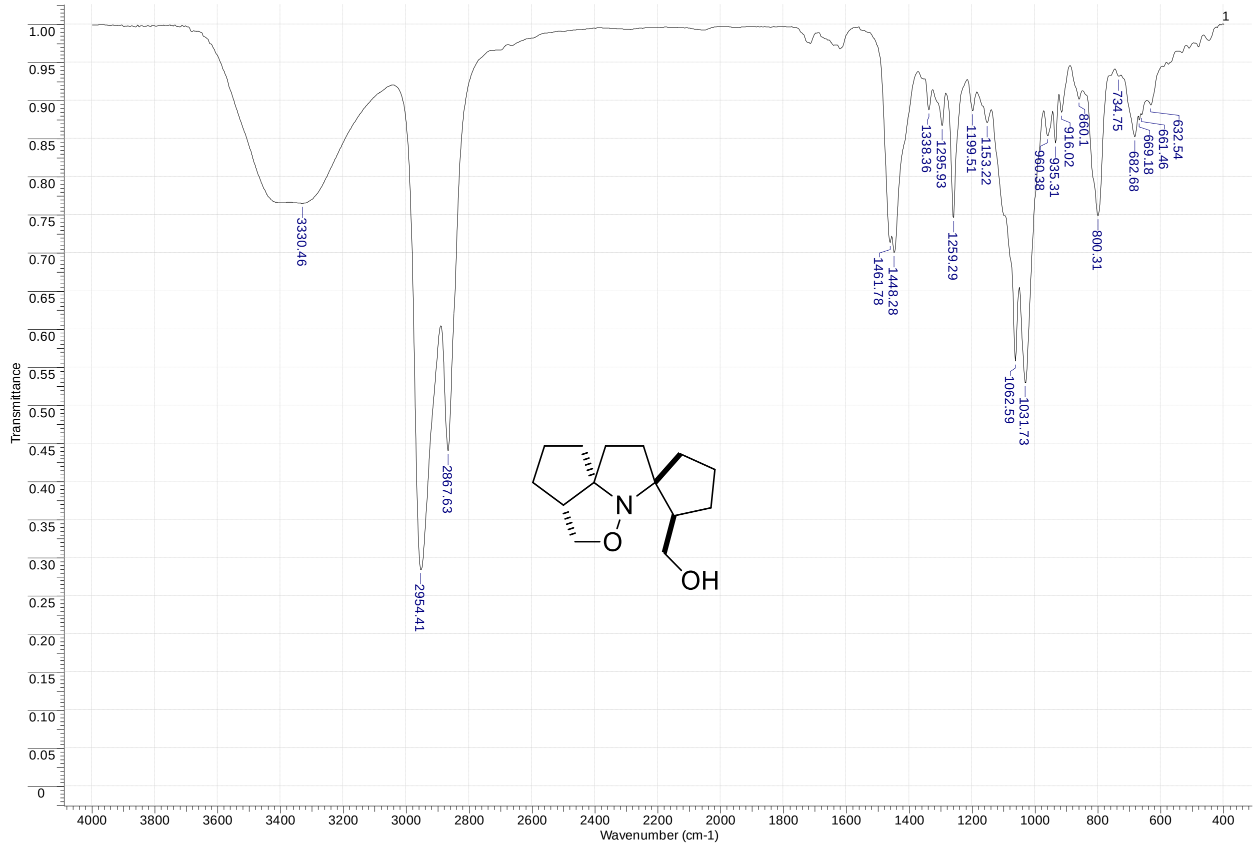

Fig. S4. IR spectrum of (1*R*(*S*),2*R*(*S*),6a′*R*(*S*),9a′*R*(*S*))-Hexahydro-6′*H*-spiro[cyclopentan-1,3′-cyclopenta[*c*]pyrrolo[1,2-*b*]isoxazol]-2-ylmethanol (**13**) (neat)

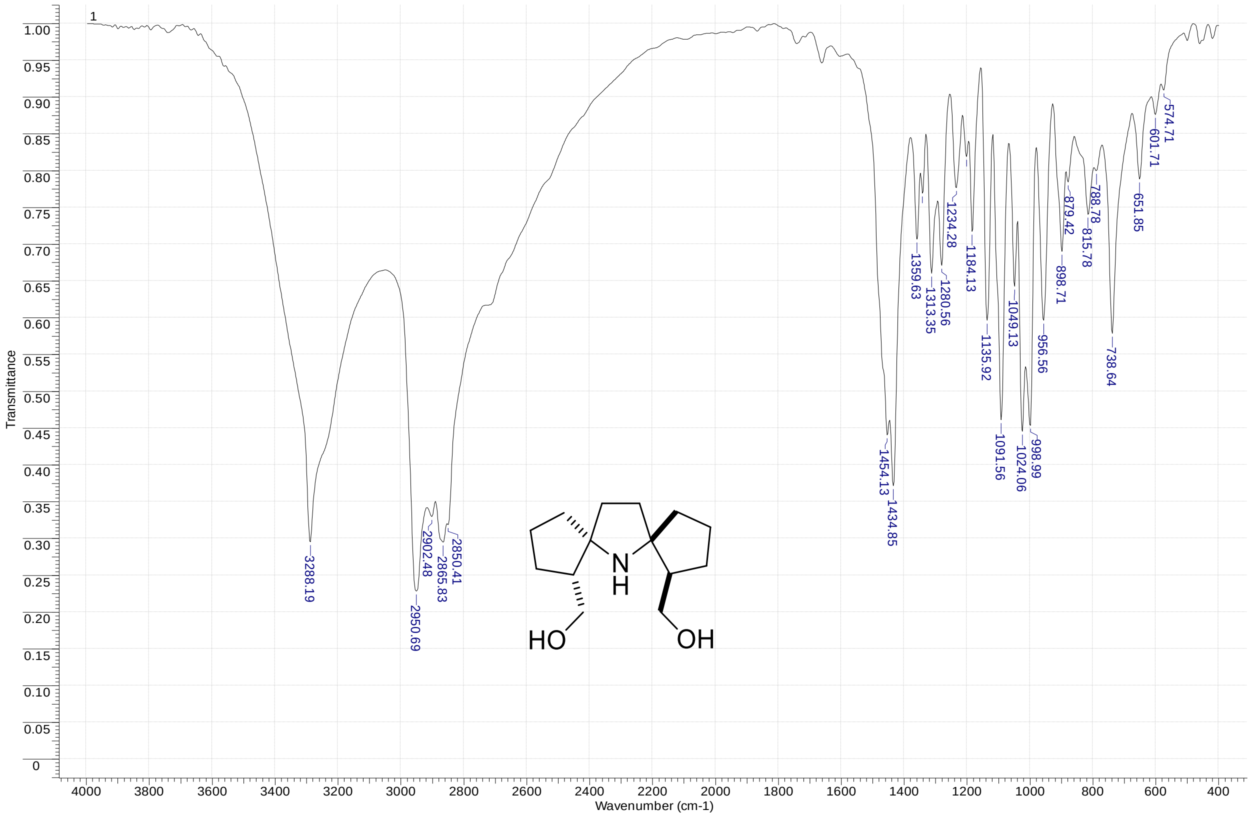

Fig. S5. IR spectrum of (1*R*(*S*),5*R*(*S*),7*R*(*S*),8*R*(*S*))-6-azadispiro[4.1.4.2]tridecan-1,8-diyldimethanol (**14**) (KBr)

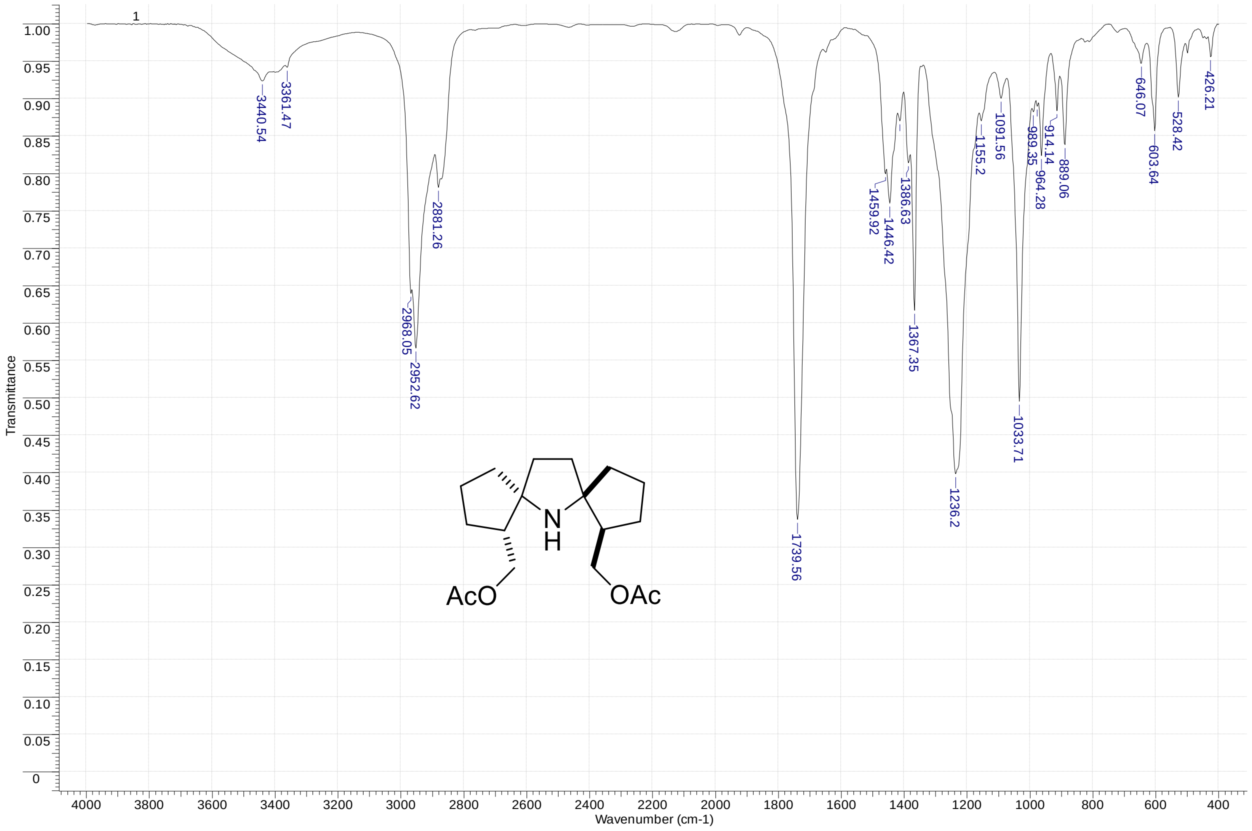

Fig. S6. IR spectrum of (1*R*(*S*),5*R*(*S*),7*R*(*S*),8*R*(*S*))-6-azadispiro[4.1.4.2]tridecan-1,8-diyldi(methylene) diacetate (**16**) (neat)

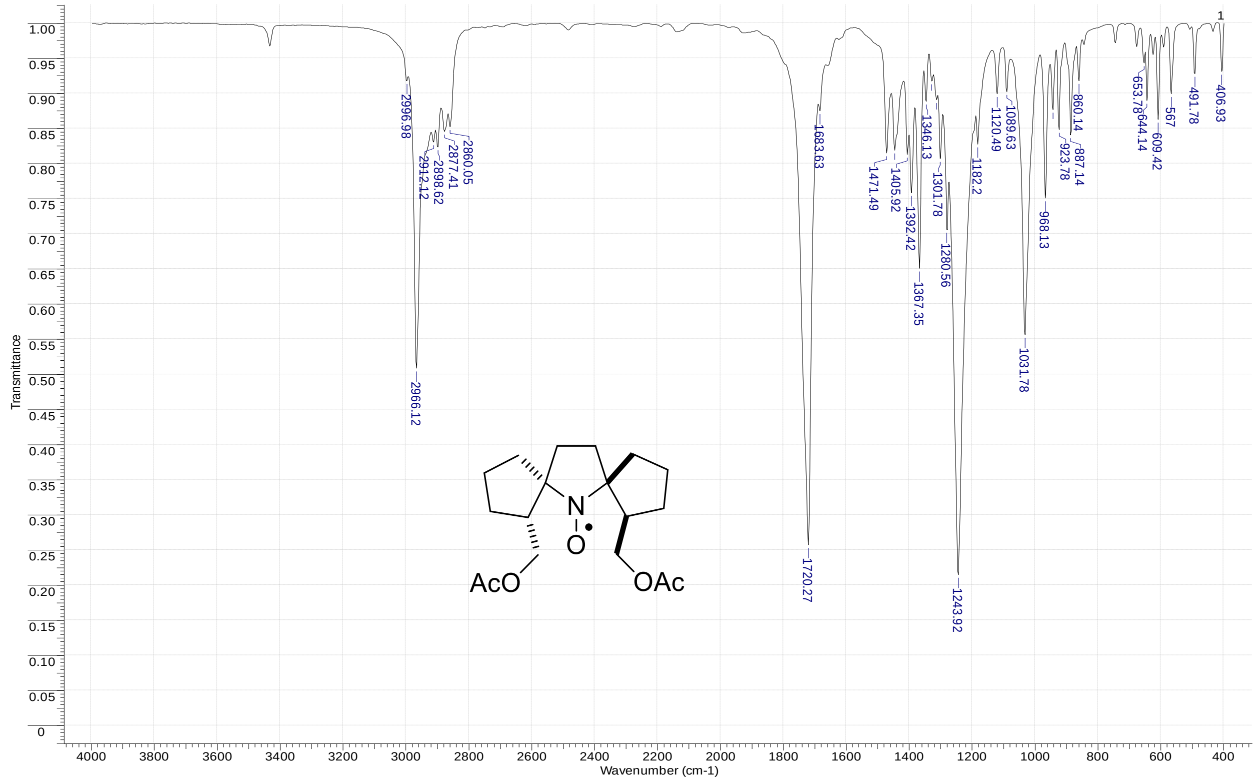

Fig. S7. IR spectrum of 1*R*(*S*),5*R*(*S*),7*R*(*S*),8*R*(*S*))-1,8-Bis[(acetyloxy)methyl]-6-azadispiro[4.1.4.2]tridecane-6-oxyl (**17**) (KBr)

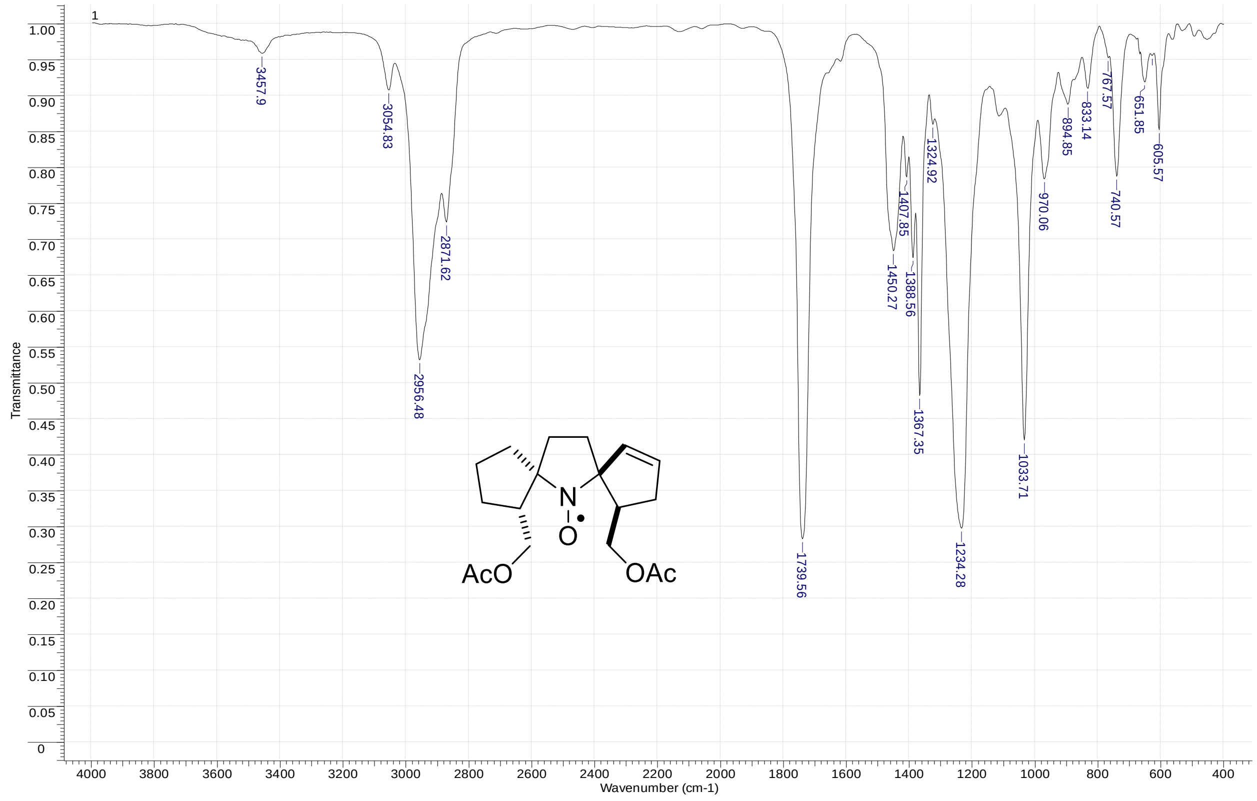

Fig. S8. IR spectrum of (4*R*(*S*),5*S*(*R*),7*R*(*S*),8*R*(*S*))-4,8-Bis[(acetyloxy)methyl]-6-azadispiro[4.1.4.2]tridec-1-ene-6-oxyl (**18**) (neat)

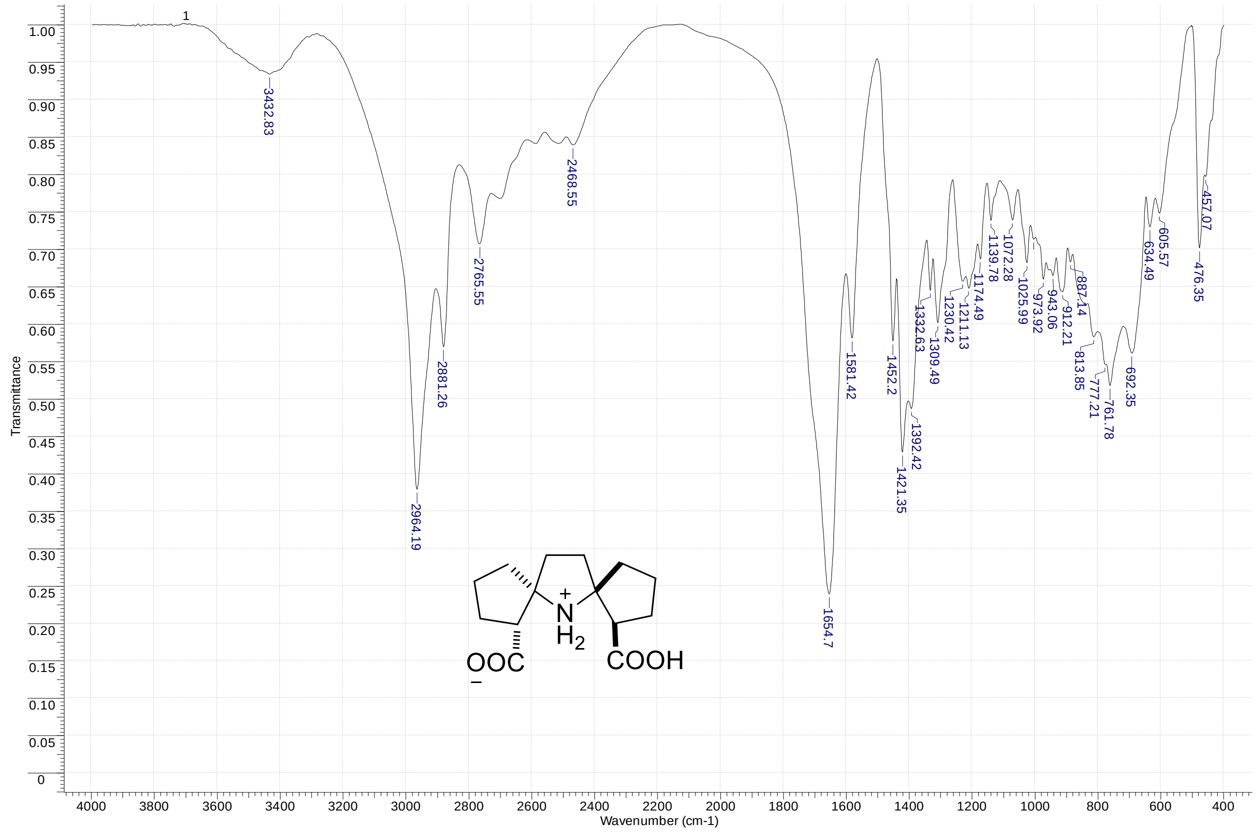

Fig. S9. IR spectrum of. (1*R*(*S*),5*R*(*S*),7*R*(*S*),8*R*(*S*))-6-Azadispiro[4.1.4.2]tridecan-1,8-dicarboxylic acid **20** (KBr)

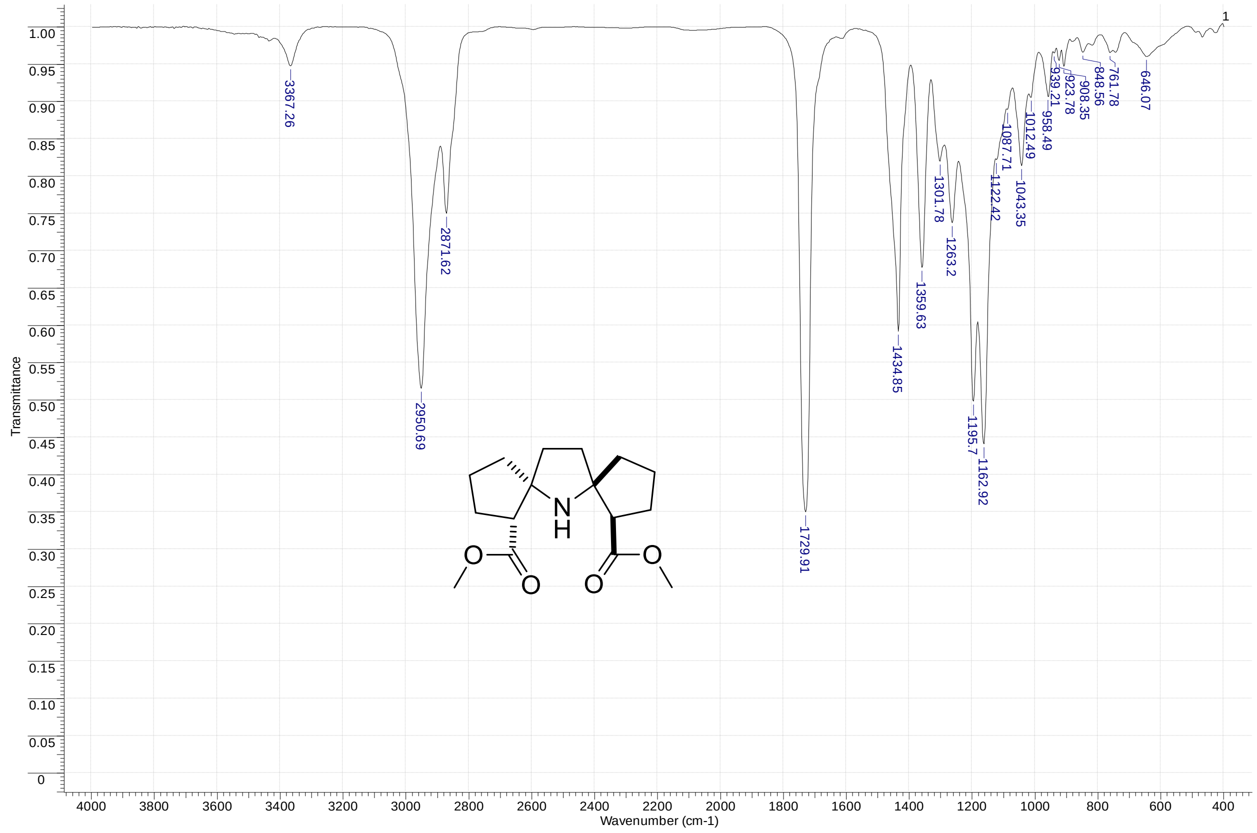

Fig. S10. IR spectrum of Dimethyl (1*R*(*S*),5*R*(*S*),7*R*(*S*),8*R*(*S*))-6-azadispiro[4.1.4.2]tridecan-1,8-dicarboxylate (**21**) (neat)

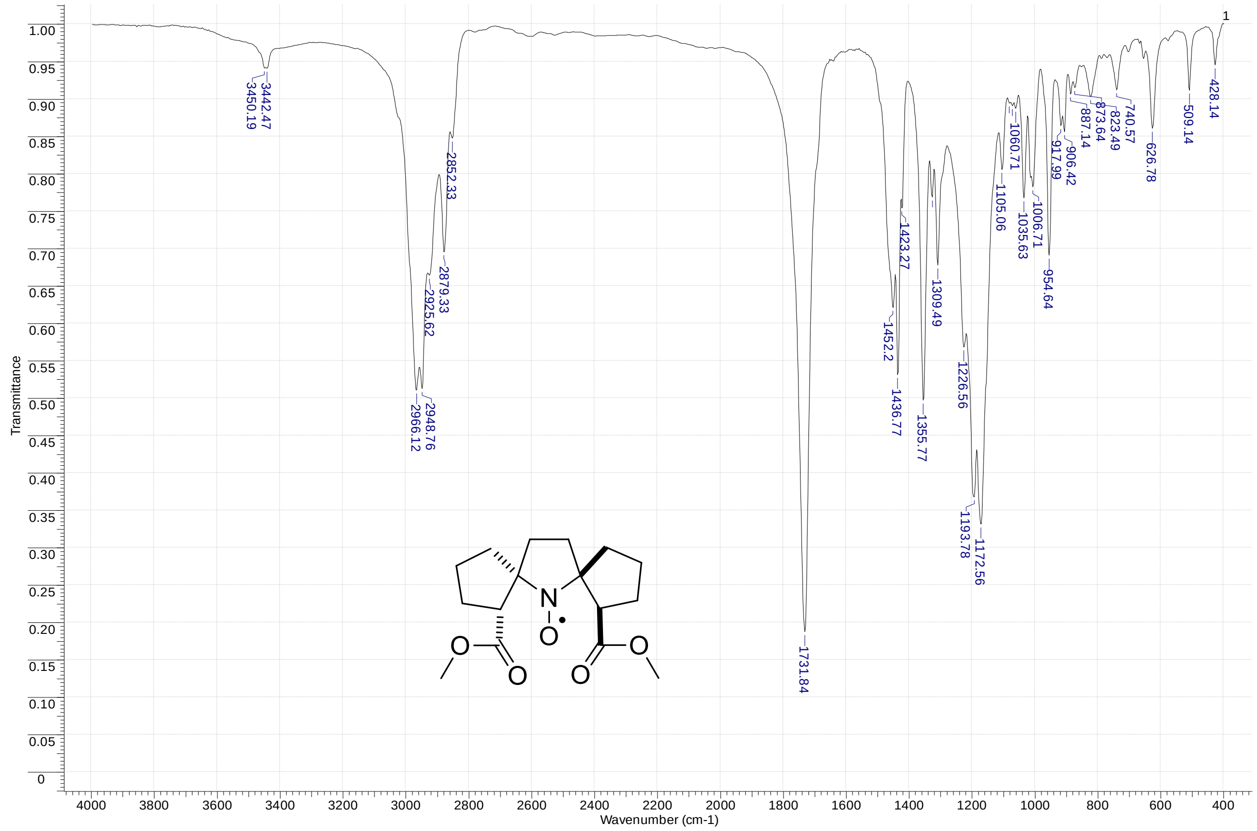

Fig. S11. IR spectrum of (1*R*(*S*),5*R*(*S*),7*R*(*S*),8*R*(*S*))-1,8-Bis(methoxycarbonyl)-6-azadispiro[4.1.4.2]tridecane-6-oxyl (**22**) (KBr)

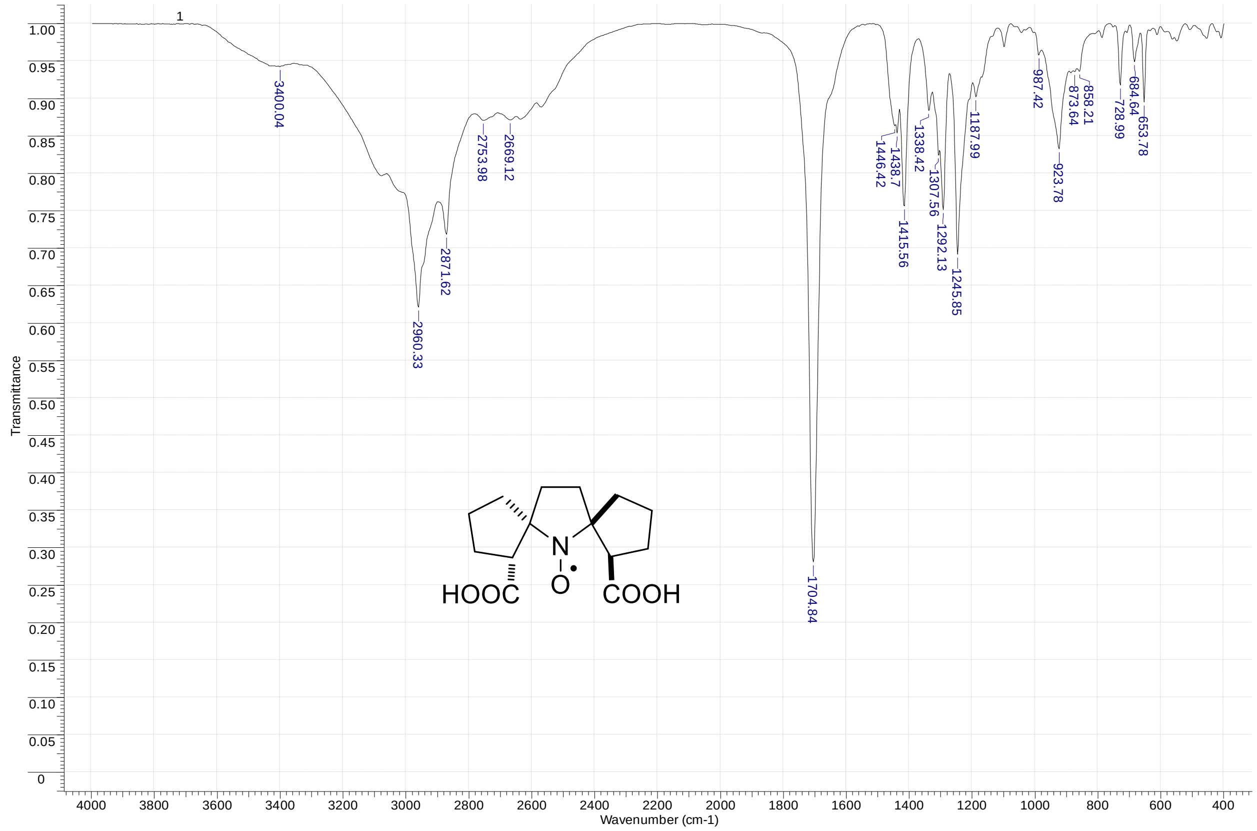

Fig. S12. IR spectrum of 1*R*(*S*),5*R*(*S*),7*R*(*S*),8*R*(*S*))-1,8-Dicarboxy-6-azadispiro[4.1.4.2]tridecane-6-oxyl (**23a**) (KBr)

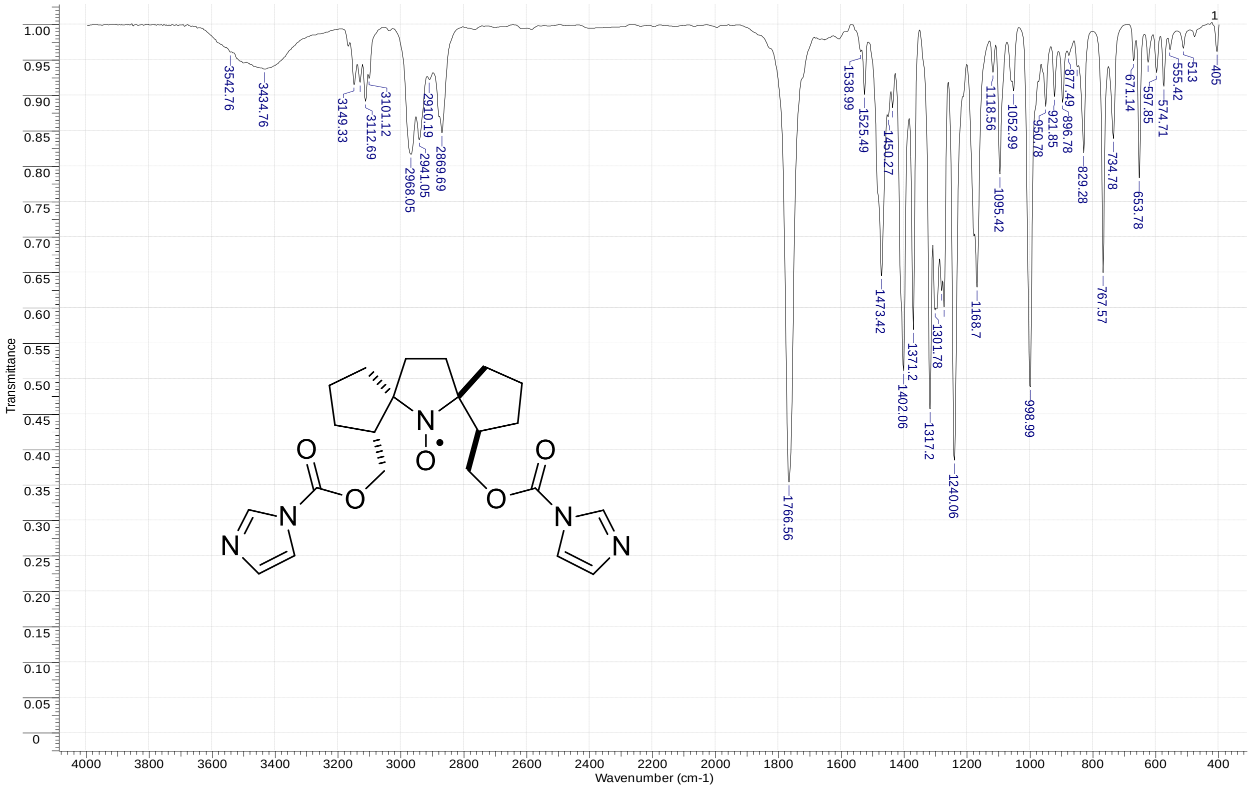

Fig. S13. IR spectrum of (1*R*(*S*),5*R*(*S*),7*R*(*S*),8*R*(*S*))-1,8-Bis[({1*H*-imidazol-1-ylcarbonyl}oxy)methyl]-6-azadispiro[4.1.4.2]tridecane-6-oxyl (**26**) (KBr)

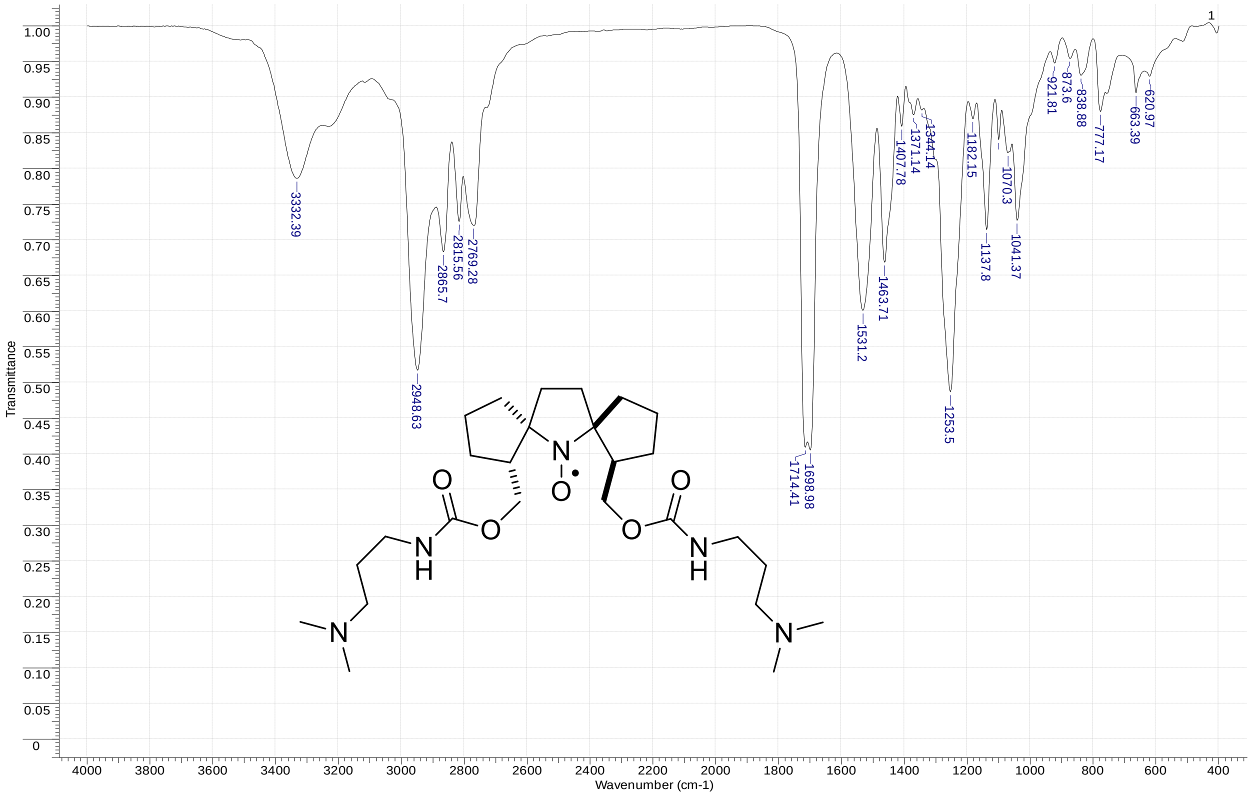

Fig. S14. IR spectrum of (1*R*(*S*),5*R*(*S*),7*R*(*S*),8*R*(*S*))-1,8-Bis[(([{3-(dimethylamino)propyl}amino]carbonyl)oxy)methyl]-6-azadispiro[4.1.4.2]tridecane-6-oxyl (**27**) (neat)

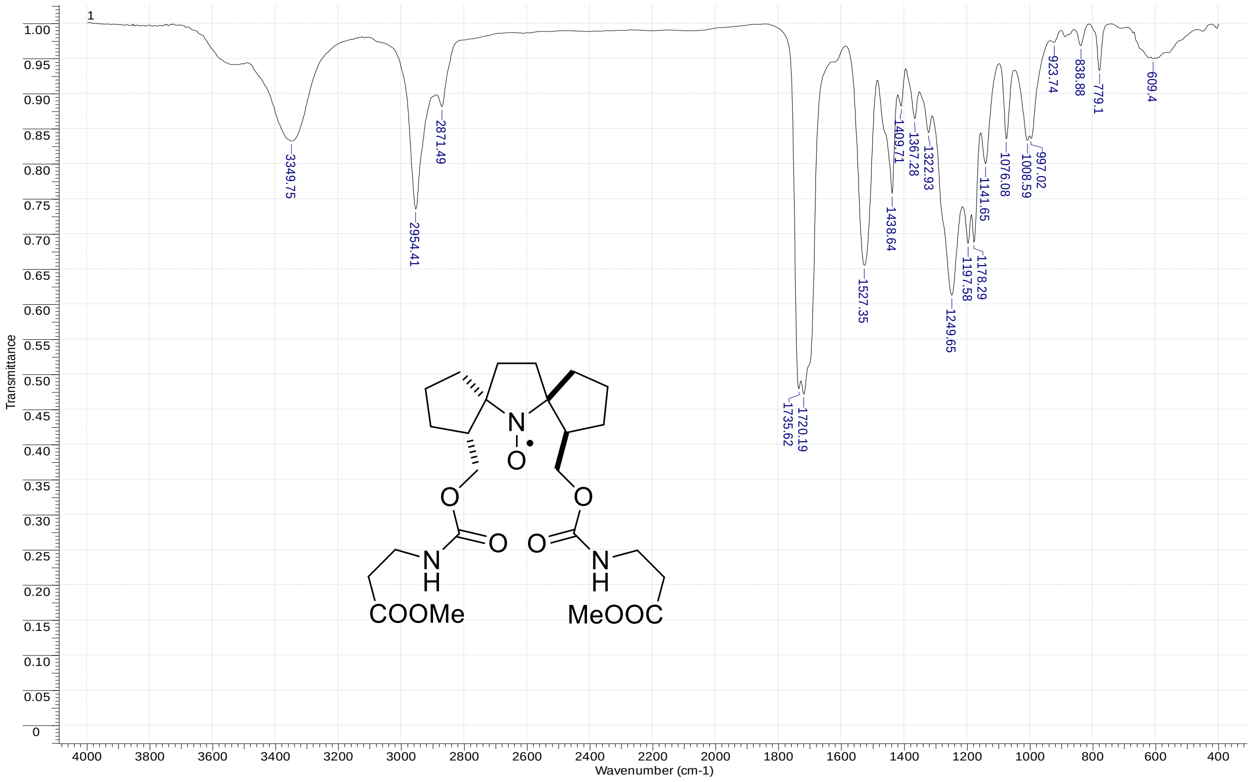

Fig. S15. IR spectrum of 1*R*(*S*),5*R*(*S*),7*R*(*S*),8*R*(*S*)-1,8-Bis[((([{3-methoxy-3-oxopropyl}amino]carbonyl)oxy)methyl]-6-azadispiro[4.1.4.2]tridecane-6-oxyl (**28**) (neat)

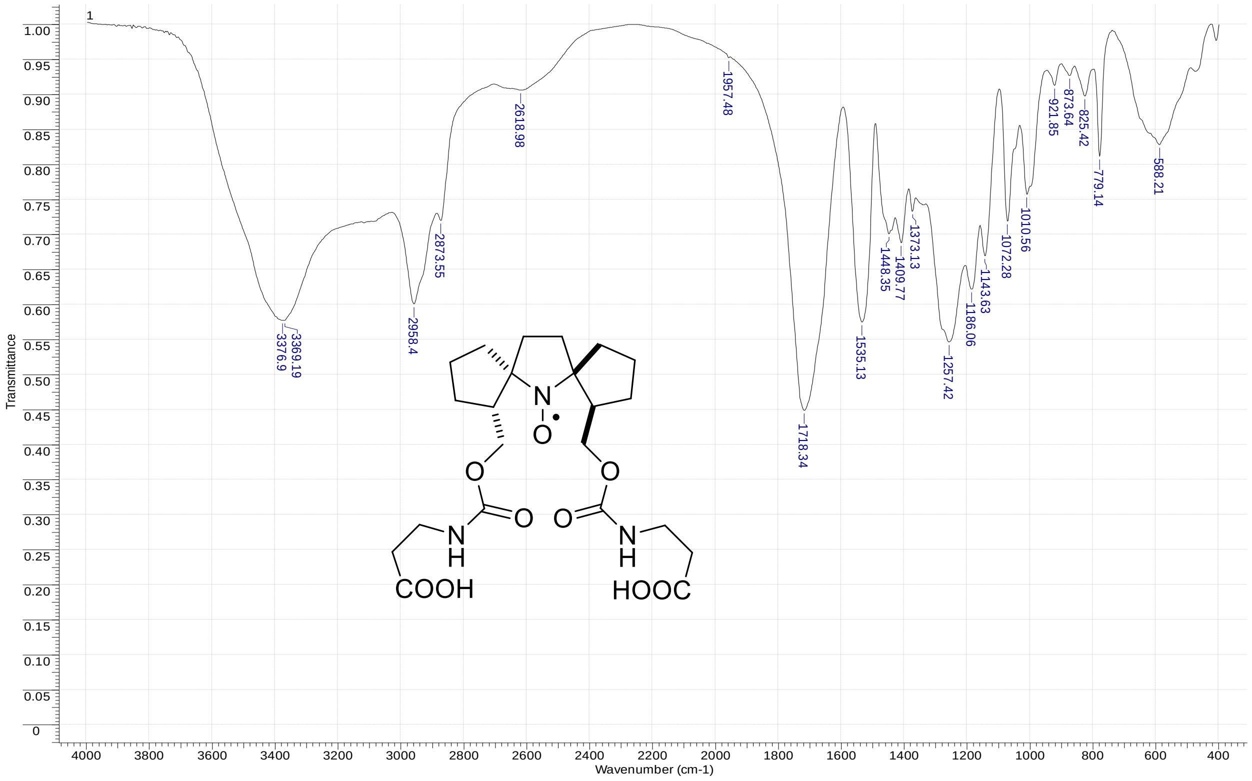

Fig. S16. IR spectrum of 1*R*(*S*),5*R*(*S*),7*R*(*S*),8*R*(*S*)-1,8-Bis[((([{2-carboxyethyl}amino]carbonyl)oxy)methyl]-6-azadispiro[4.1.4.2]tridecane-6-oxyl (**29**) (KBr)

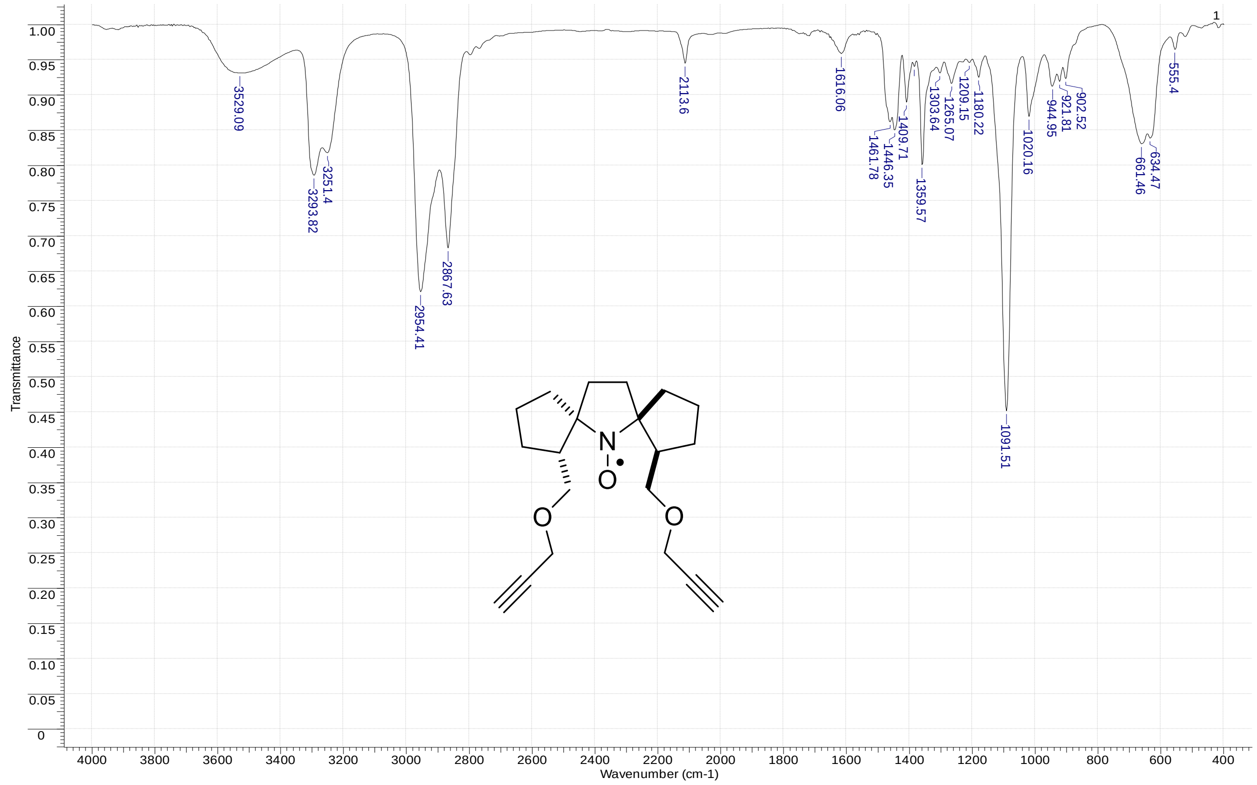

Fig. S17. IR spectrum of (1*R*(*S*),5*R*(*S*),7*R*(*S*),8*R*(*S*))-1,8-Bis[(prop-2-yn-1-yloxy)methyl]-6-azadispiro[4.1.4.2]tridecane-6-oxyl (**30**) (neat)

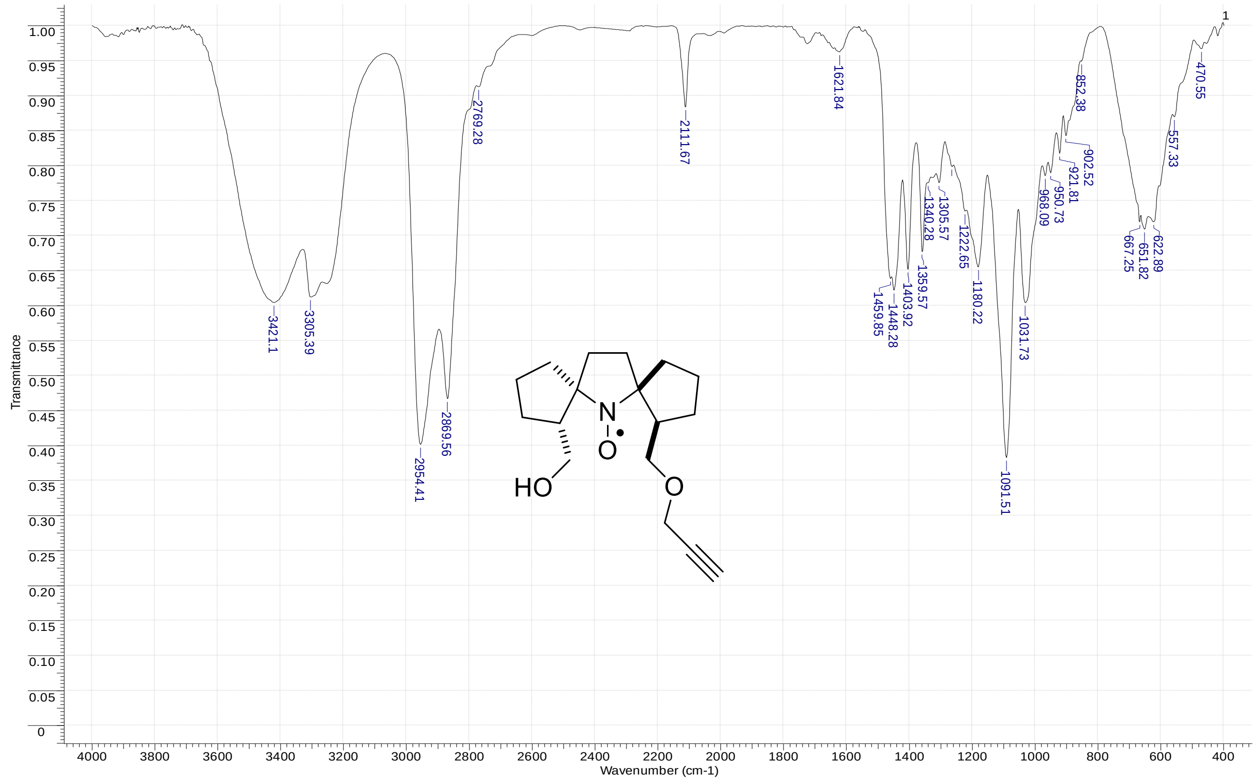

Fig. S18. IR spectrum of (1*R*(*S*),5*R*(*S*),7*R*(*S*),8*R*(*S*))-1-(Hydroxymethyl)-8-[(prop-2-yn-1-yloxy)methyl]-6-azadispiro[4.1.4.2]tridecane-6-oxyl (**31**) (neat)

International Journal of Molecular Sciences

MDPI

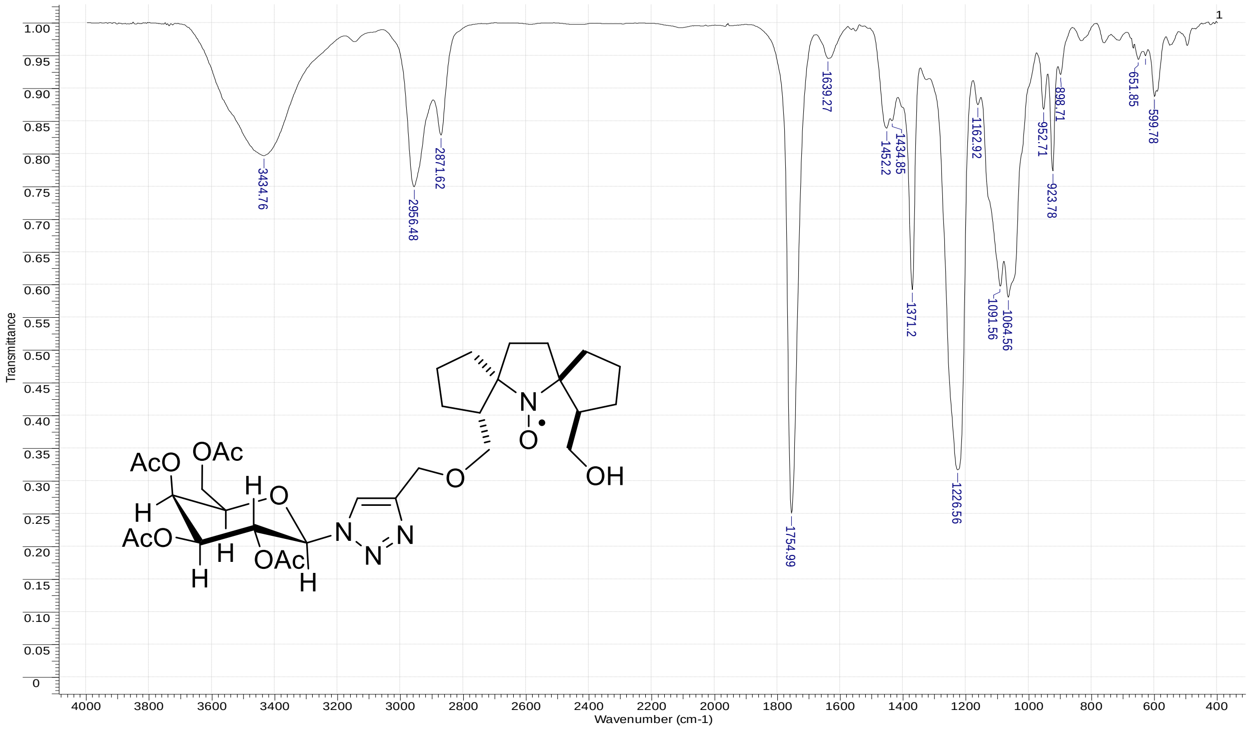

Fig. S19. IR spectrum of (1*R*(*S*),5*R*(*S*),7*R*(*S*),8*R*(*S*))-1-(Hydroxymethyl-8-({(1-[2,3,4,6-tetra-*O*-acetyl-β-D-galactopyranosyl]-1*H*-1,2,3-triazol-4-yl)methoxy}methyl)-6-azadispiro[4.1.4.2]tridecane-6-oxyl (**32**) (KBr)

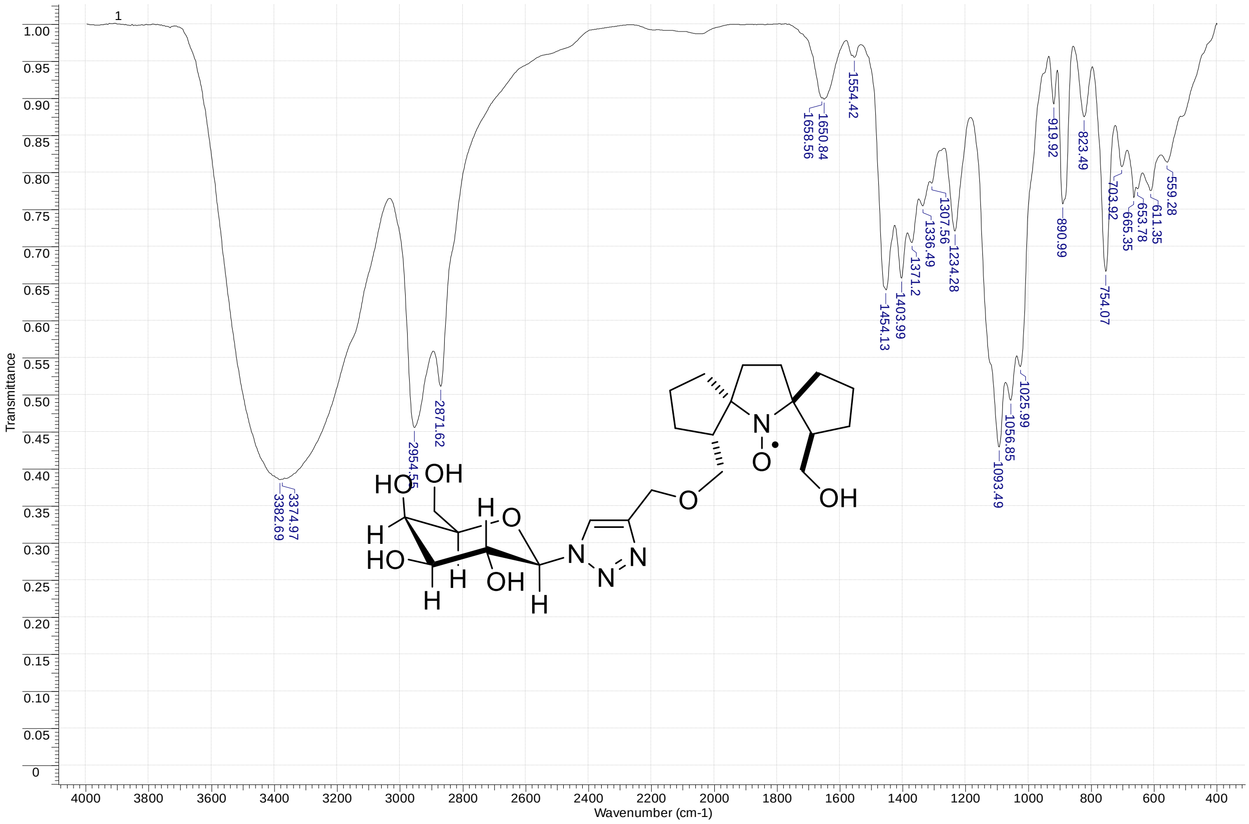

Fig. S20. IR spectrum of (1*R*(*S*),5*R*(*S*),7*R*(*S*),8*R*(*S*))-1-(Hydroxymethyl)-8-({(1-[β-D-galactopyranosyl]-1*H*-1,2,3-triazol-4-yl)methoxy}methyl)-6-azadispiro[4.1.4.2]tridecane-6-oxyl (**33**) (KBr)

International Journal of
Molecular Sciences

MDPI

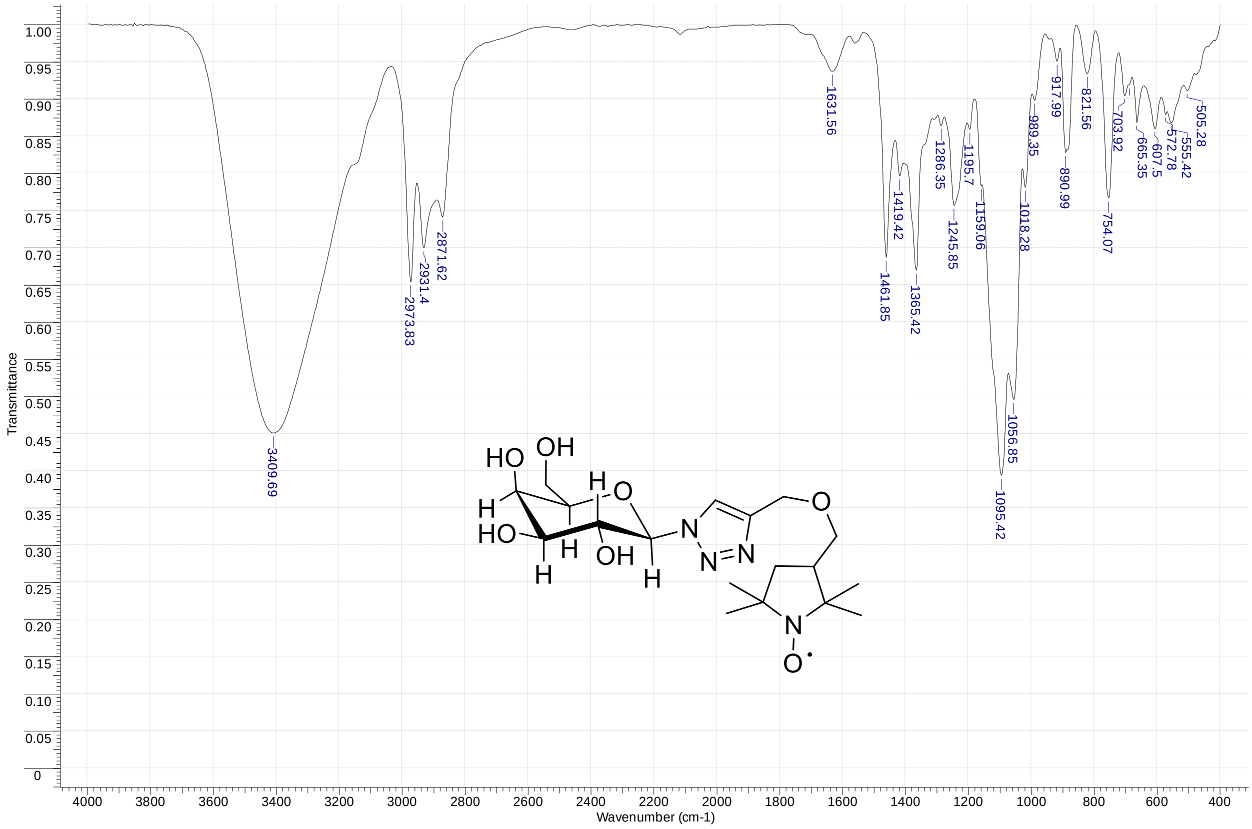

Fig. S21. IR spectrum of 2,2,5,5-Tetramethyl-3-(((1-(β-D-galactopyranosyl)-1*H*-1,2,3-triazol-4-yl)methoxy)methyl)pyrrolidine-1-oxyl (**35**) (KBr)

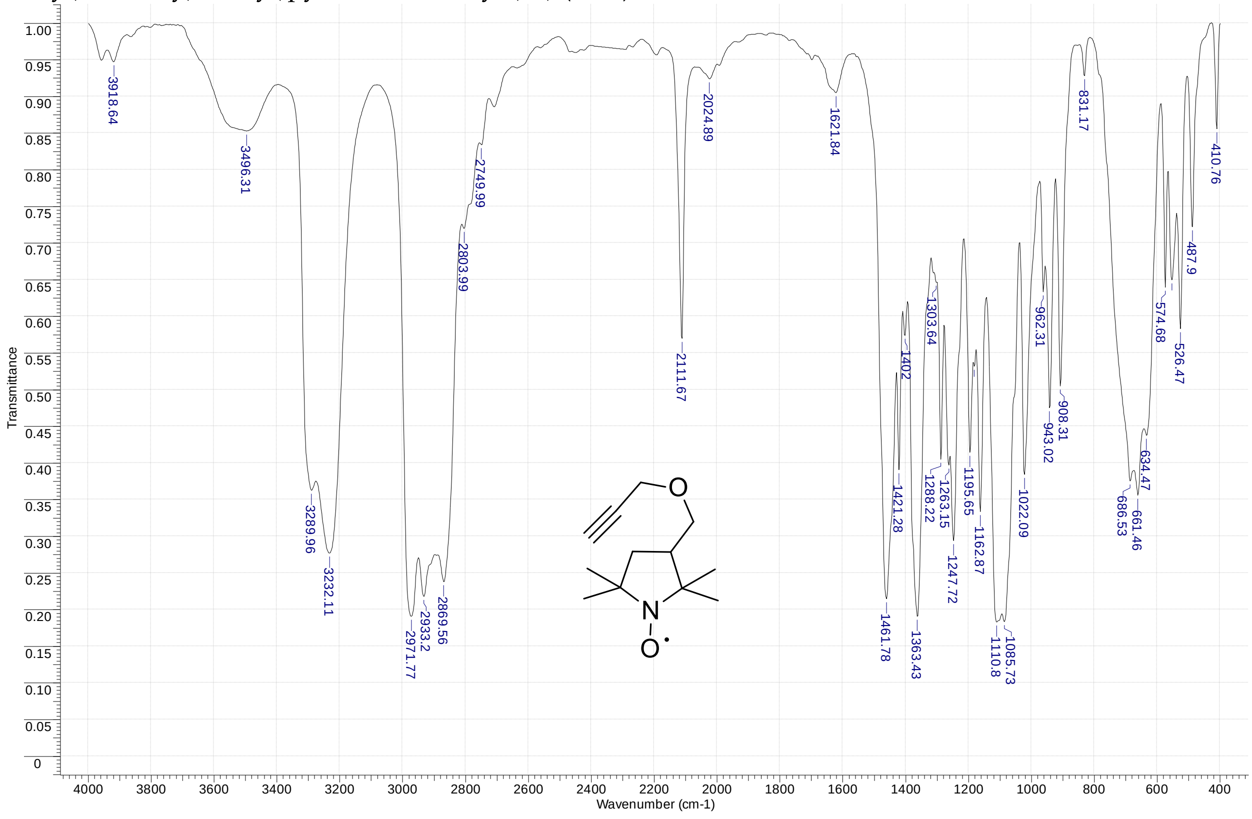

Fig. S22. IR spectrum of 2,2,5,5-Tetramethyl-3-((prop-2-ynyloxy)methyl)pyrrolidine-1-oxyl (**37**) (KBr)

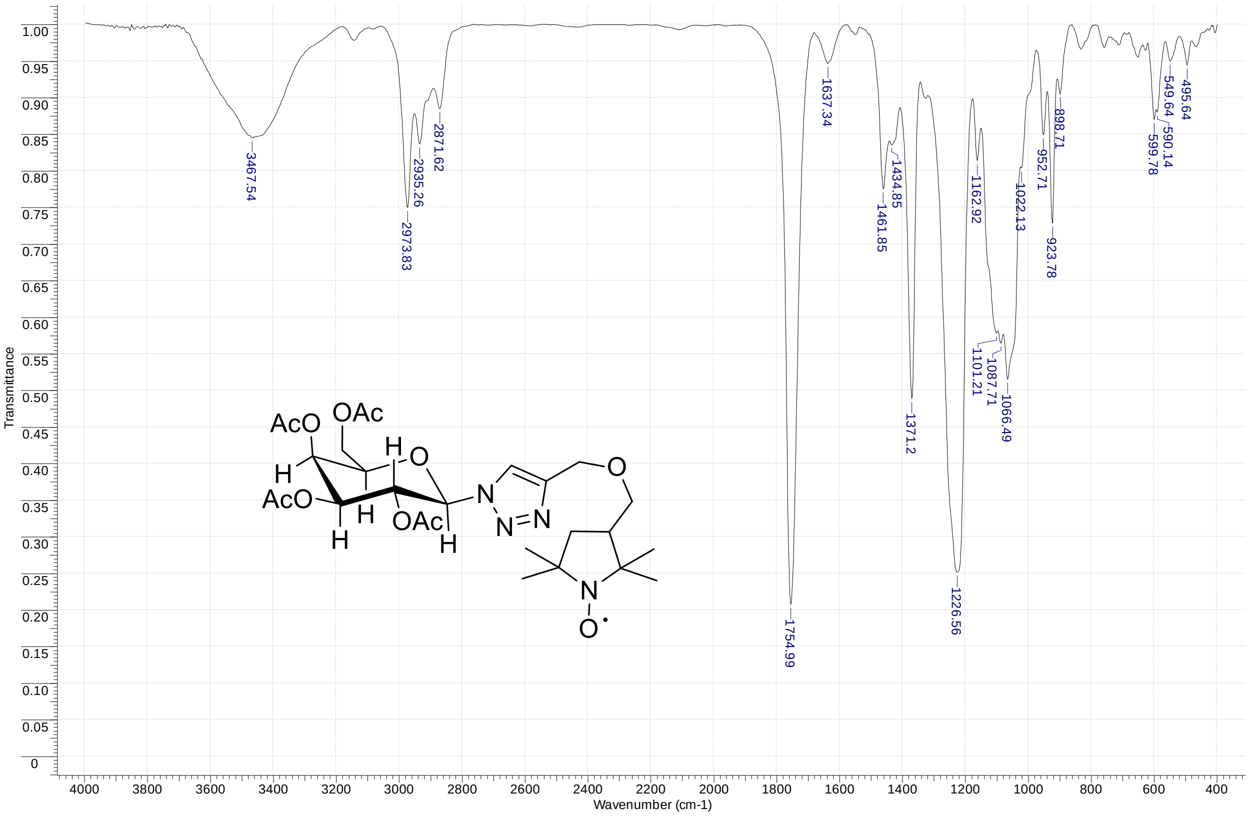

Fig. S23. IR spectrum of 2,2,5,5-Tetramethyl-3-(((1-(2,3,4,6-tetra-*O*-acetyl-β-D-galactopyranosyl)-1*H*-1,2,3-triazol-4-yl)methoxy)methyl)pyrrolidine-1-oxyl (**38**) (KBr)

3. UV spectral data

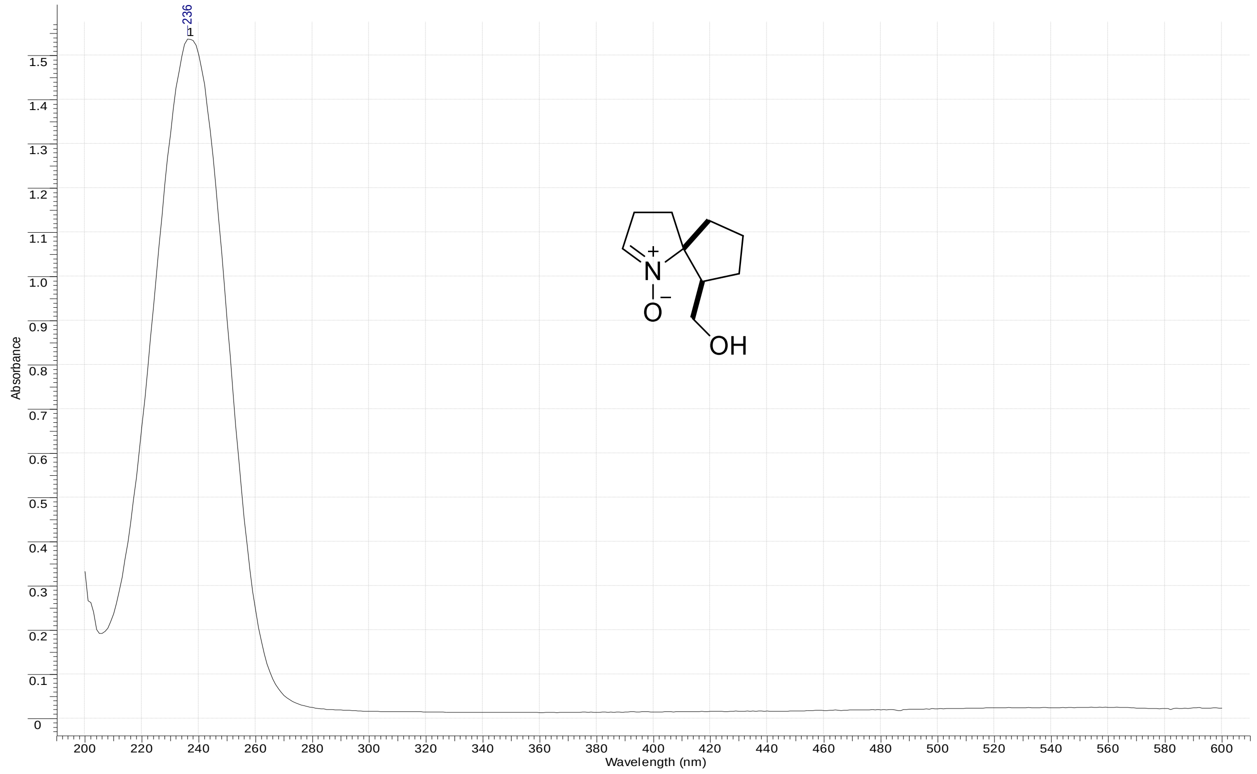

Fig. S24. UV spectrum of [(5*R*(*S*),6*R*(*S*))-1-Oxido-1-azaspiro[4.4]non-1-en-6-yl]methanol (**9**) in EtOH (0.429mg/25ml, L=2cm)

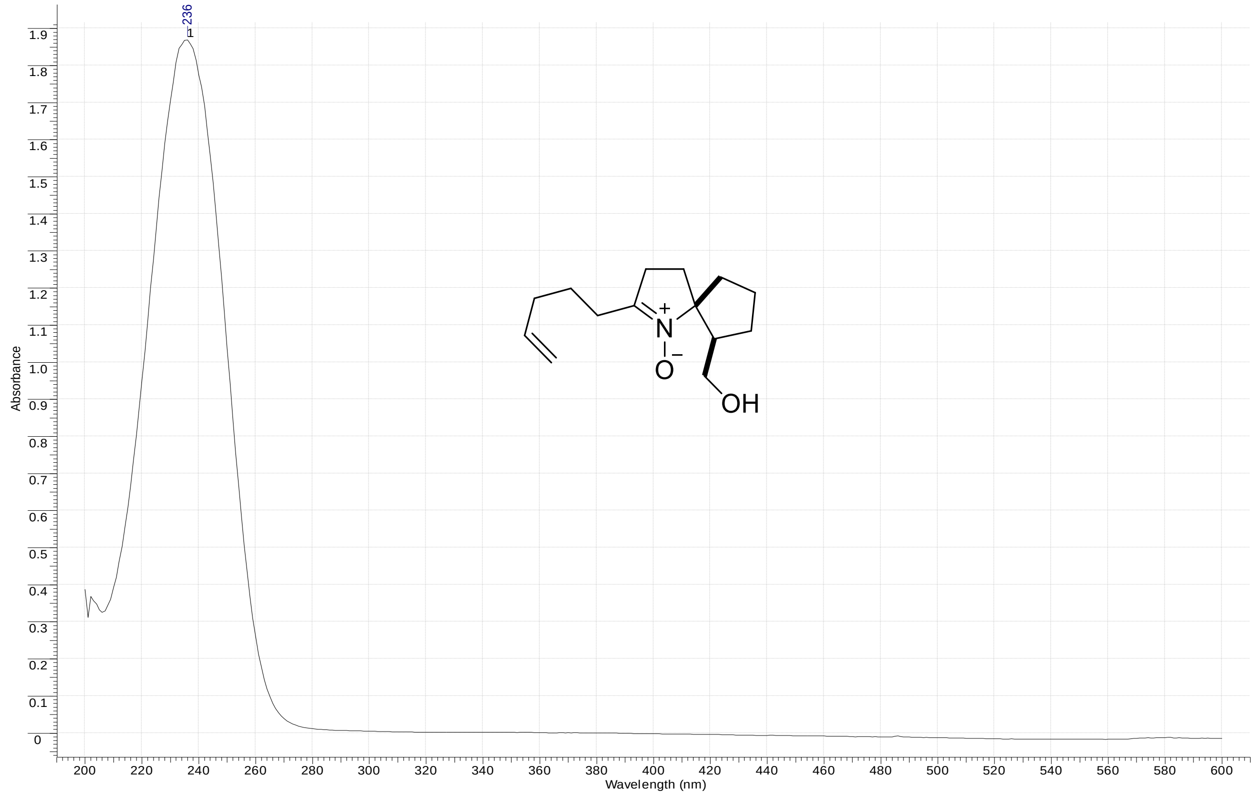

Fig. S25. UV spectrum of [(5*R*(*S*),6*R*(*S*))-1-Oxido-2-pent-4-en-1-yl-1-azaspiro[4.4]non-1-en-6-yl]methanol (**12**) in EtOH (0.596mg/25ml, L=2cm)

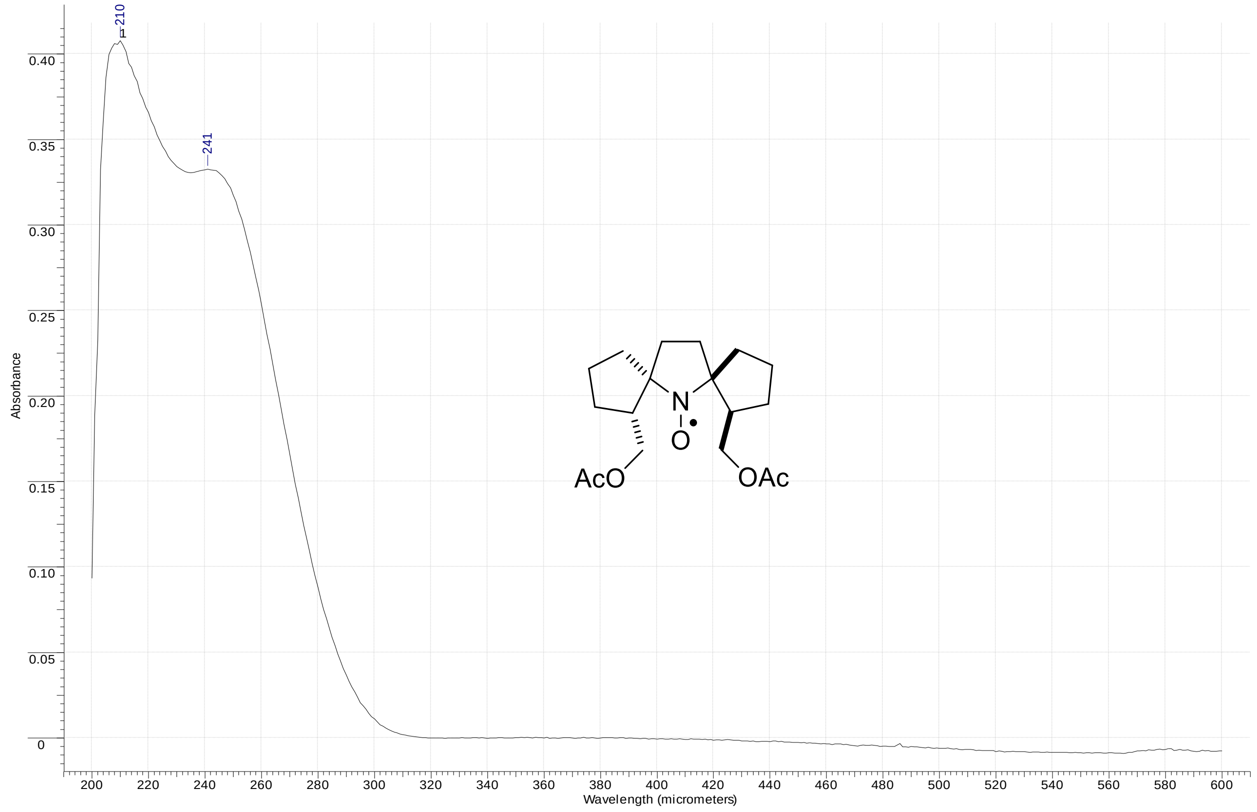

Fig. S26. UV spectrum of (1*R*(*S*),5*S*(*R*),7*R*(*S*),8*R*(*S*))-1,8-Bis[(acetyloxy)methyl]-6-azadispiro[4.1.4.2]tridecane-6-oxyl (**17**) in EtOH (0.848mg/25ml, L=2cm)

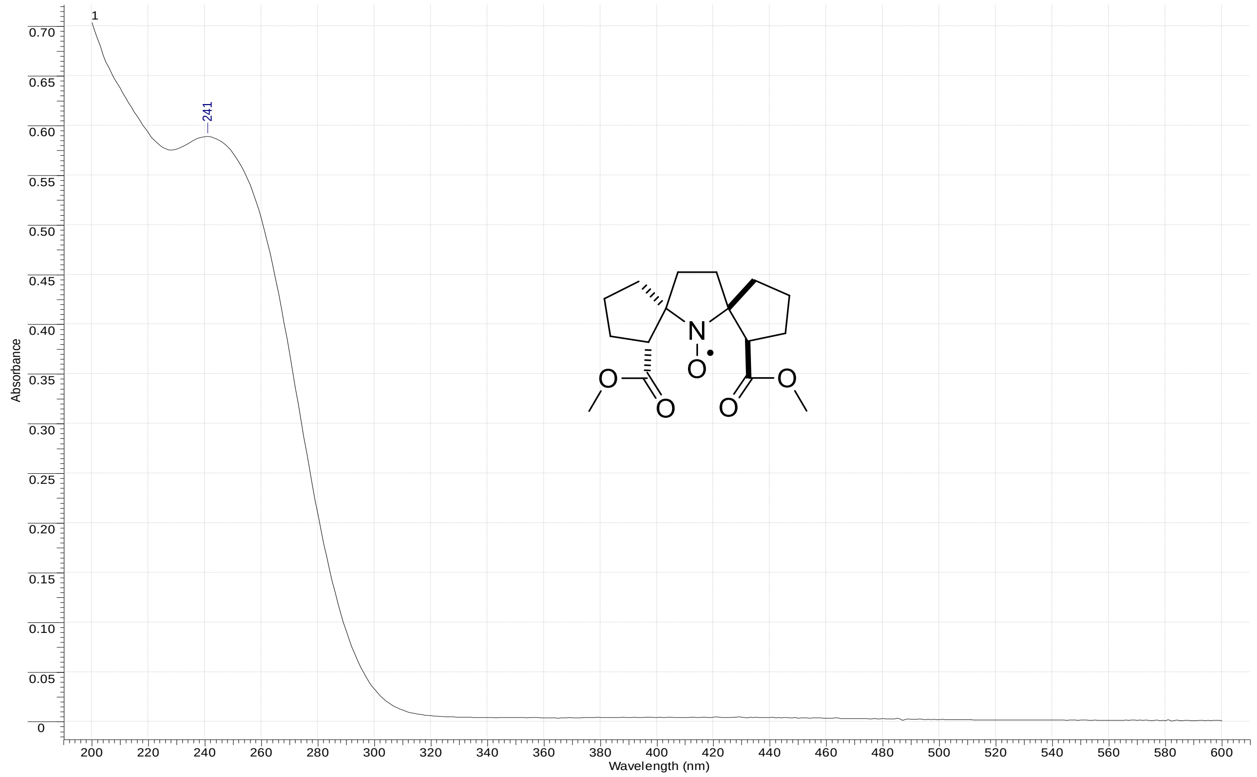

Fig. S27. UV spectrum of (1*R*(*S*),5*R*(*S*),7*R*(*S*),8*R*(*S*))-1,8-Bis[Methoxycarbonyl]-6-azadispiro[4.1.4.2]tridecane-6-oxyl (**22**) in EtOH (4.985mg/25ml, L=0.5cm)

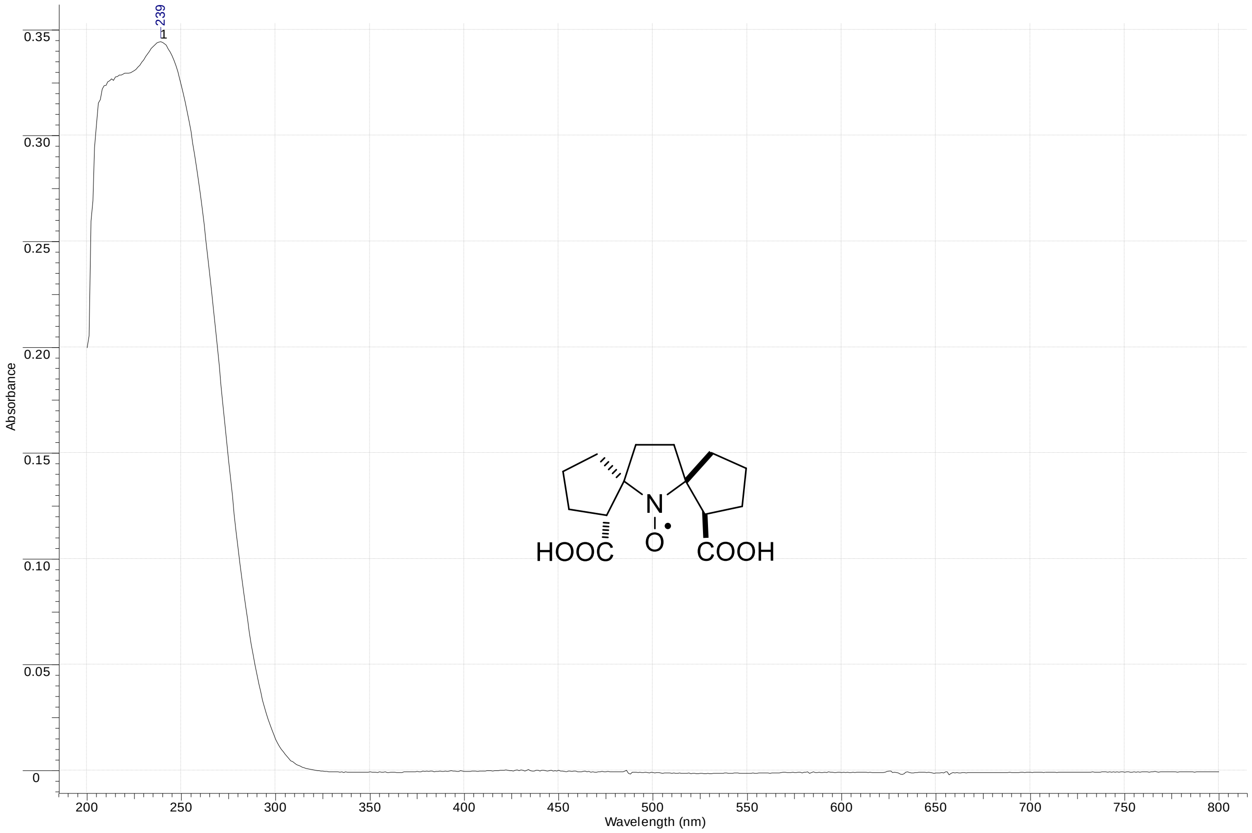

Fig. S28. UV spectrum of 1*R*(*S*),5*R*(*S*),7*R*(*S*),8*R*(*S*))-1,8-Dicarboxy-6-azadispiro[4.1.4.2]tridecane-6-oxyl (**23a**) in EtOH (0.708mg/25ml, L=2cm)

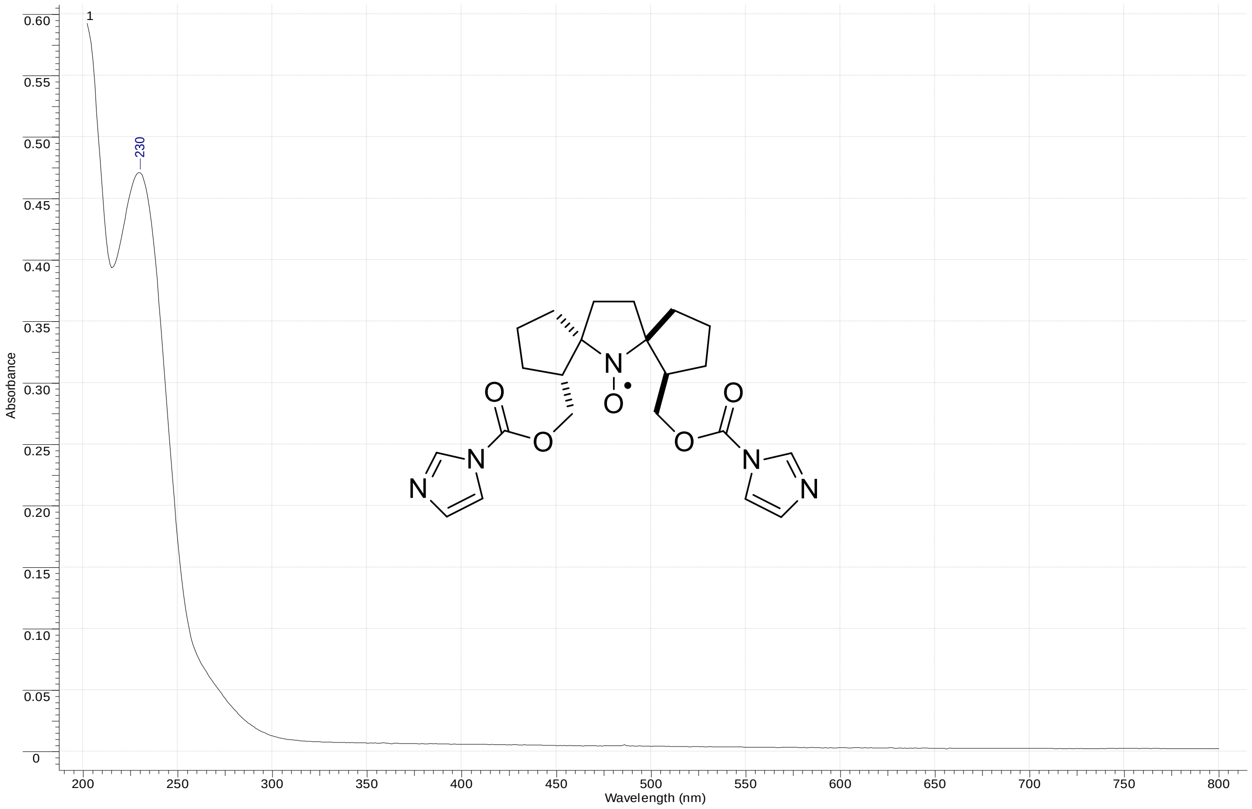

Fig. S29. UV spectrum of (1*R*(*S*),5*R*(*S*),7*R*(*S*),8*R*(*S*))-1,8-Bis[({1*H*-imidazol-1-ylcarbonyl}oxy)methyl]-6-azadispiro[4.1.4.2]tridecane-6-oxyl (**26**) in EtOH (1.150mg/25ml, L=0.5cm)

International Journal of
Molecular Sciences

MDPI

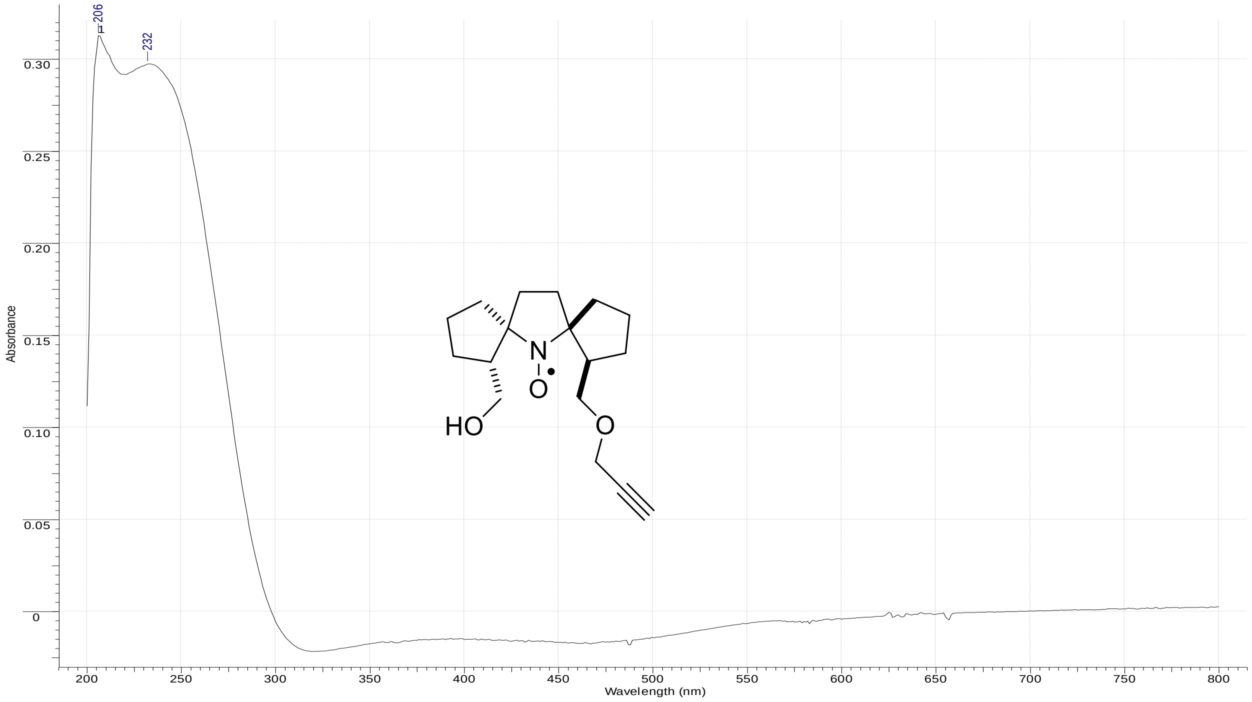

Fig. S30. UV spectrum of (1*R*(*S*),5*R*(*S*),7*R*(*S*),8*R*(*S*))-1-(Hydroxymethyl)-8-[(prop-2-yn-1-yloxy)methyl]-6-azadispiro[4.1.4.2]tridecane-6-oxyl (**31**) in EtOH (0.740mg/25ml, L=2cm)

4. ^{1}H and ^{13}C NMR spectral data

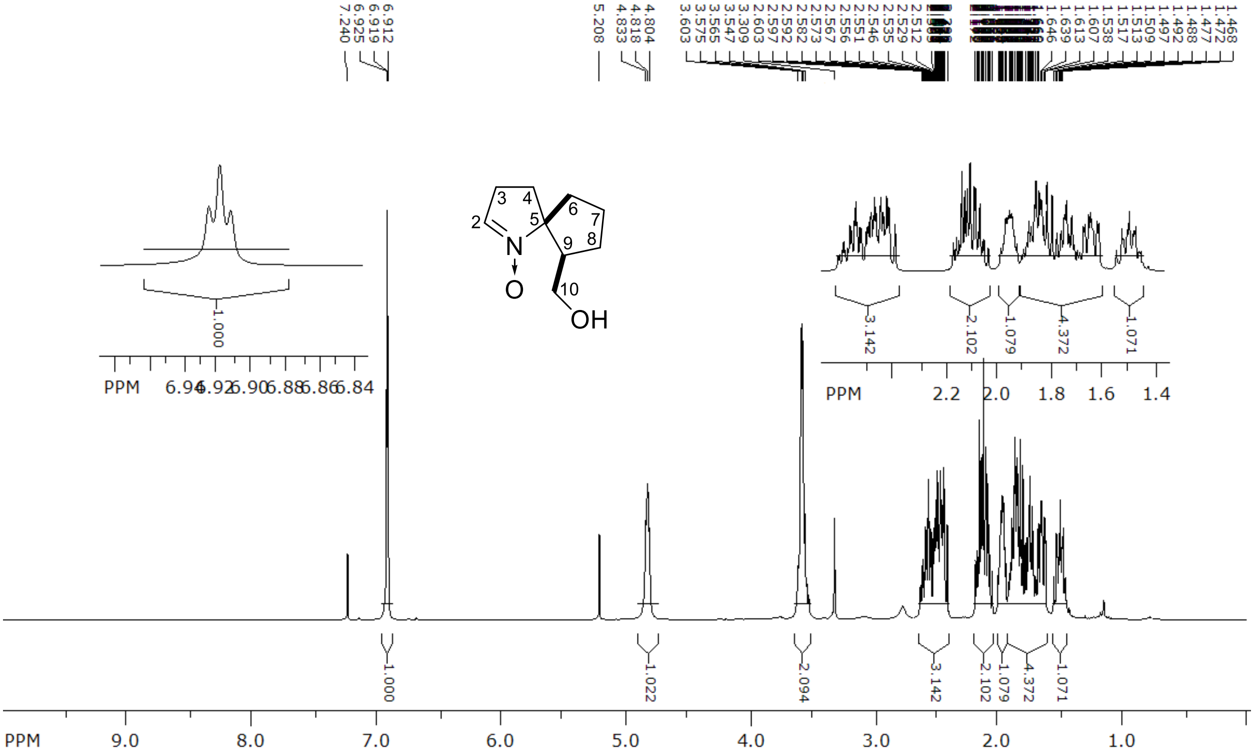

Fig. S31. ^{1}H NMR spectrum of [(5*R*(*S*),6*R*(*S*))-1-Oxido-1-azaspiro[4.4]non-1-en-6-yl]methanol (**9**) in $CDCl_3$ at 400 MHz

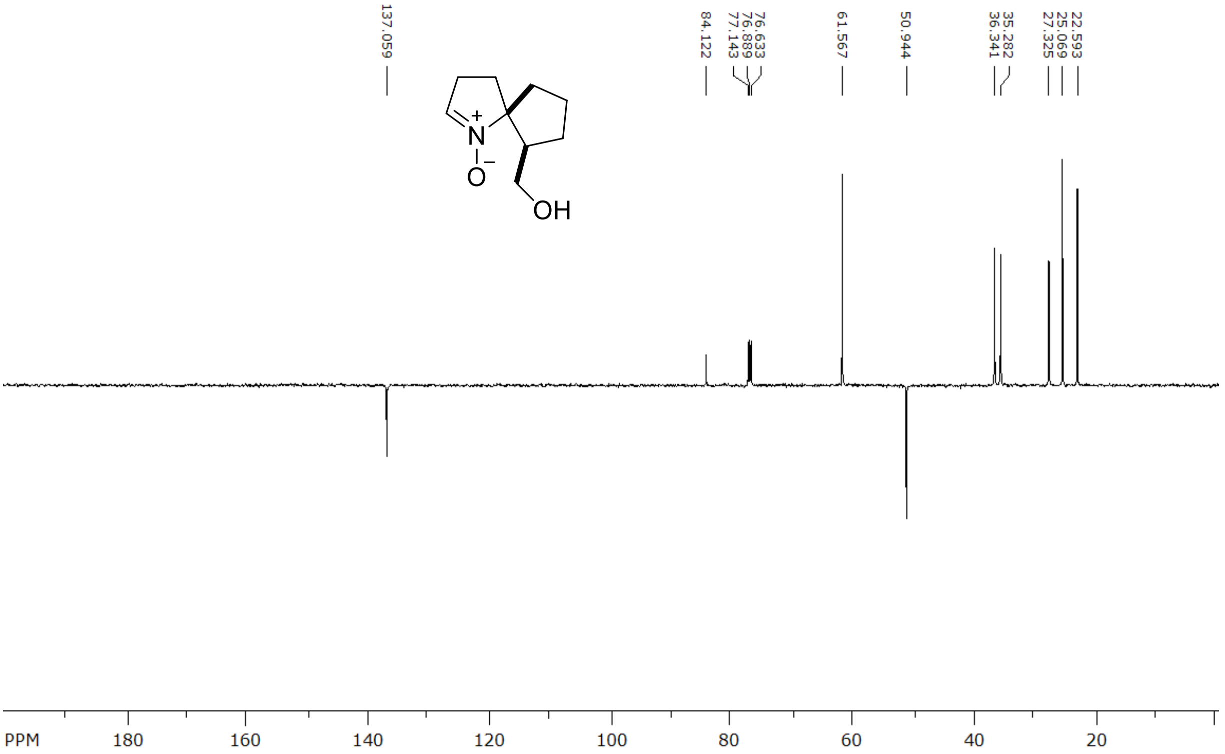

Fig. S32. ^{13}C NMR spectrum of [(5*R*(*S*),6*R*(*S*))-1-Oxido-1-azaspiro[4.4]non-1-en-6-yl]methanol (**9**) in $CDCl_3$ at 125 MHz

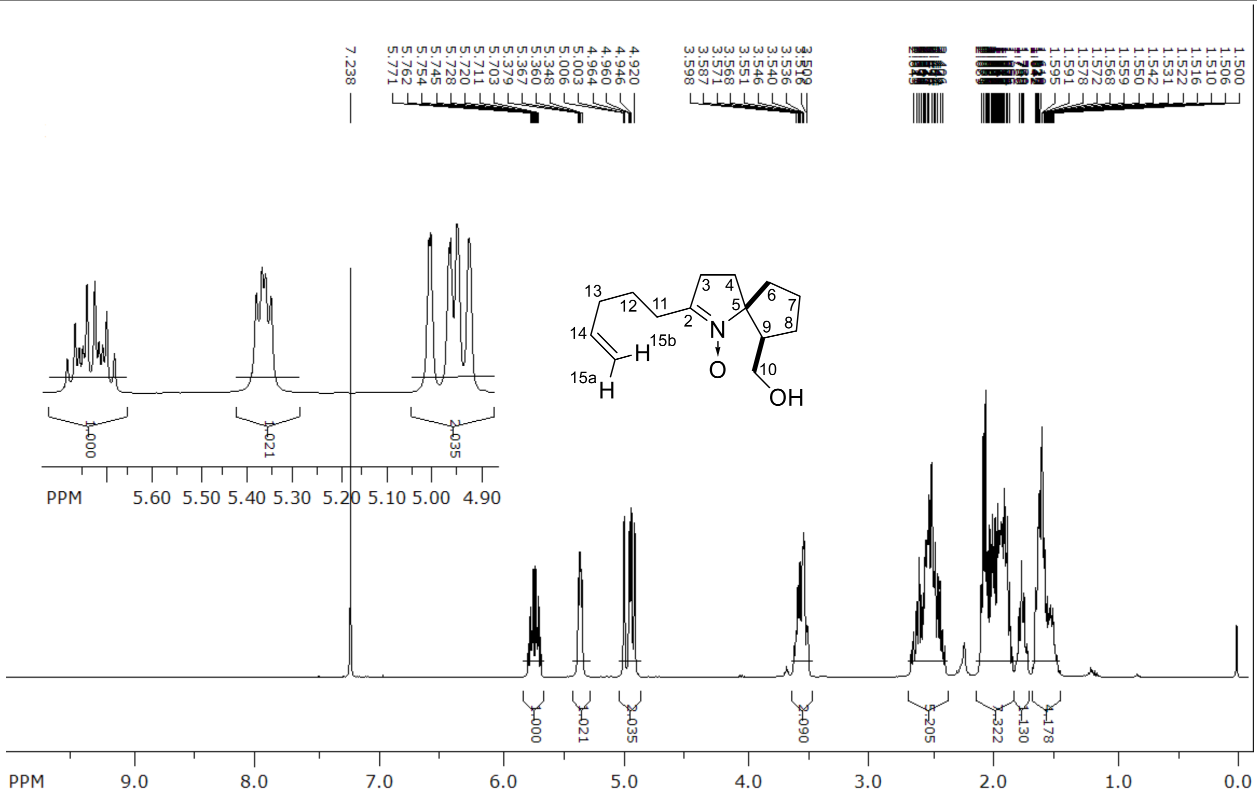

Fig. S33. ^1H NMR spectrum of [(5*R*(*S*),6*R*(*S*))-1-Oxido-2-(pent-4-en-1-yl)-1-azaspiro[4.4]non-1-en-6-yl]methanol (**12**) in $CDCl_3$ at 400 MHz

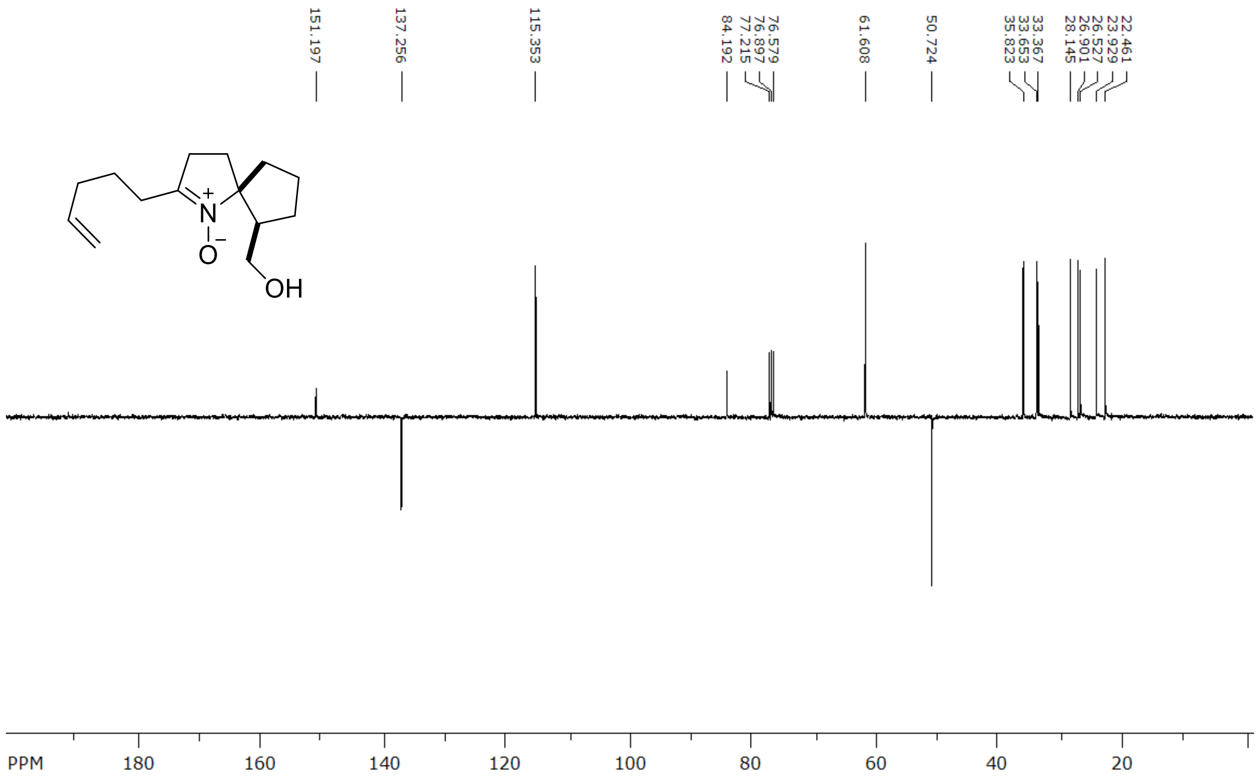

Fig. S34. ^{13}C NMR spectrum of [(5*R*(*S*),6*R*(*S*))-1-Oxido-2-(pent-4-en-1-yl)-1-azaspiro[4.4]non-1-en-6-yl]methanol (**12**) in $CDCl_3$ at 100 MHz

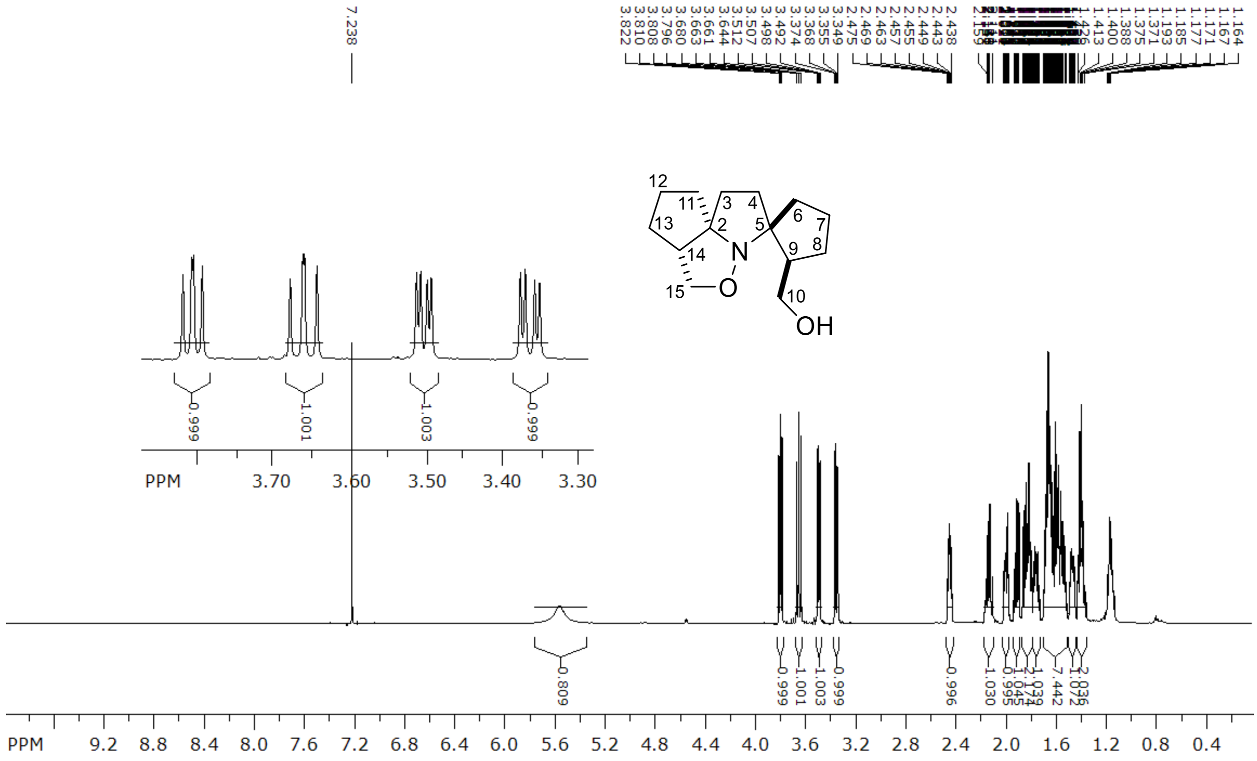

Fig. S35. ^{1}H NMR spectrum of (1*R*(*S*),2*R*(*S*),6a′*R*(*S*),9a′*R*(*S*)-Hexahydro-6′*H*-spiro[cyclopentan-1,3′-cyclopenta[*c*]pyrrolo[1,2-*b*]isoxazol]-2-ylmethanol (**13**) in $CDCl_3$ at 600 MHz

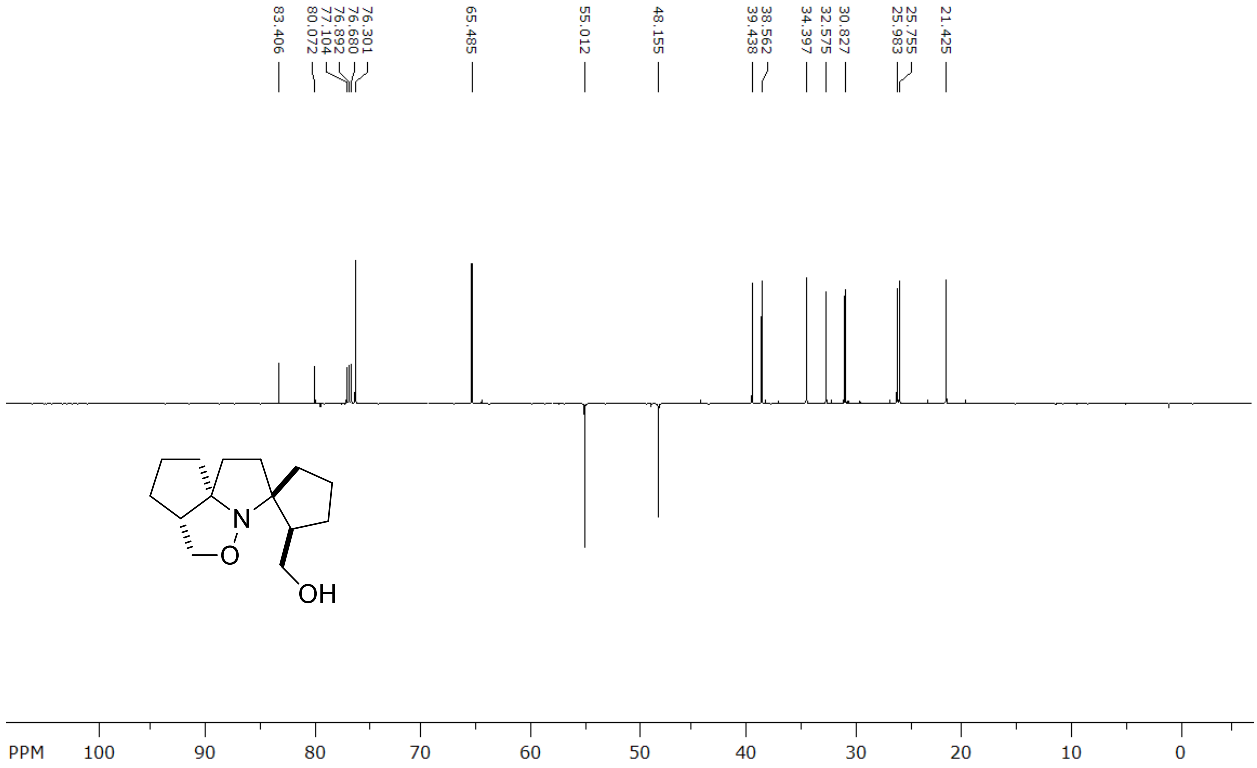

Fig. S36. ^{13}C NMR spectrum of (1*R*(*S*),2*R*(*S*),6a′*R*(*S*),9a′*R*(*S*)-Hexahydro-6′*H*-spiro[cyclopentan-1,3′-cyclopenta[*c*]pyrrolo[1,2-*b*]isoxazol]-2-ylmethanol (**13**) in $CDCl_3$ at 150 MHz

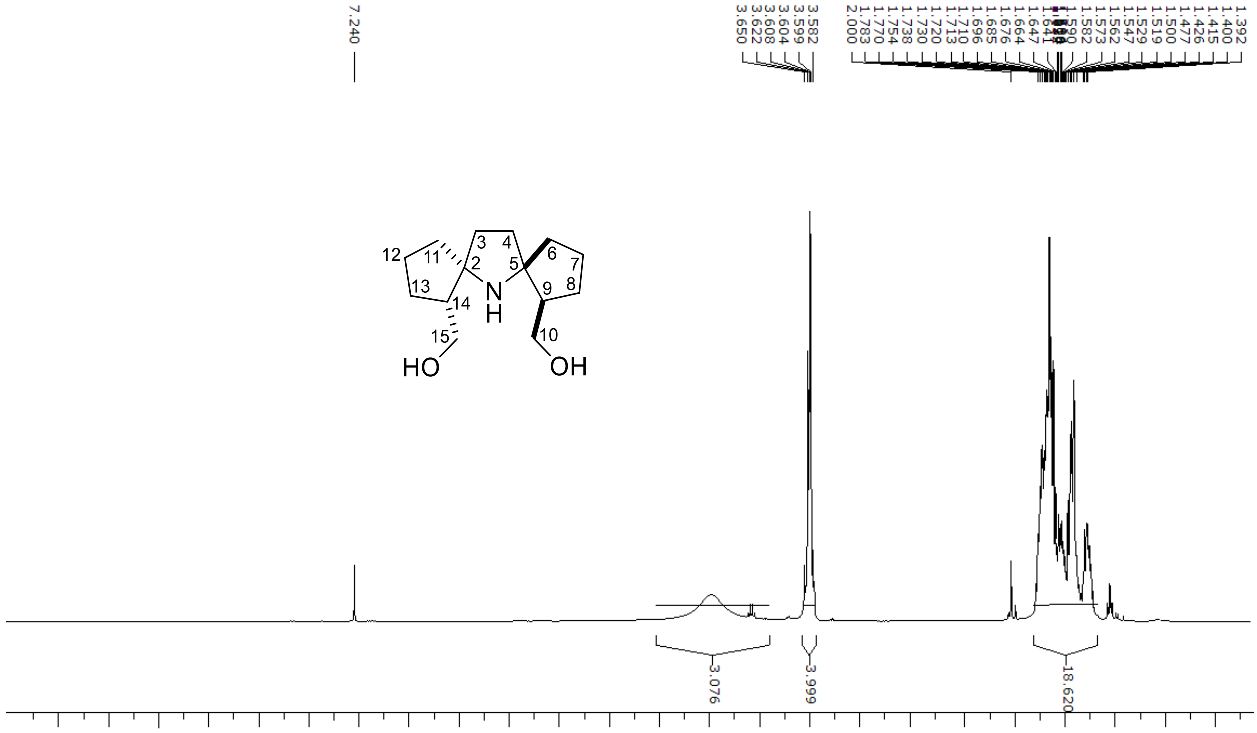

Fig. S37. ^{1}H NMR spectrum of (1*R*(*S*),5*R*(*S*),7*R*(*S*),8*R*(*S*))-6-azadispiro[4.1.4.2]tridecan-1,8-diyldimethanol (**14**) in $CDCl_3$ at 400 MHz

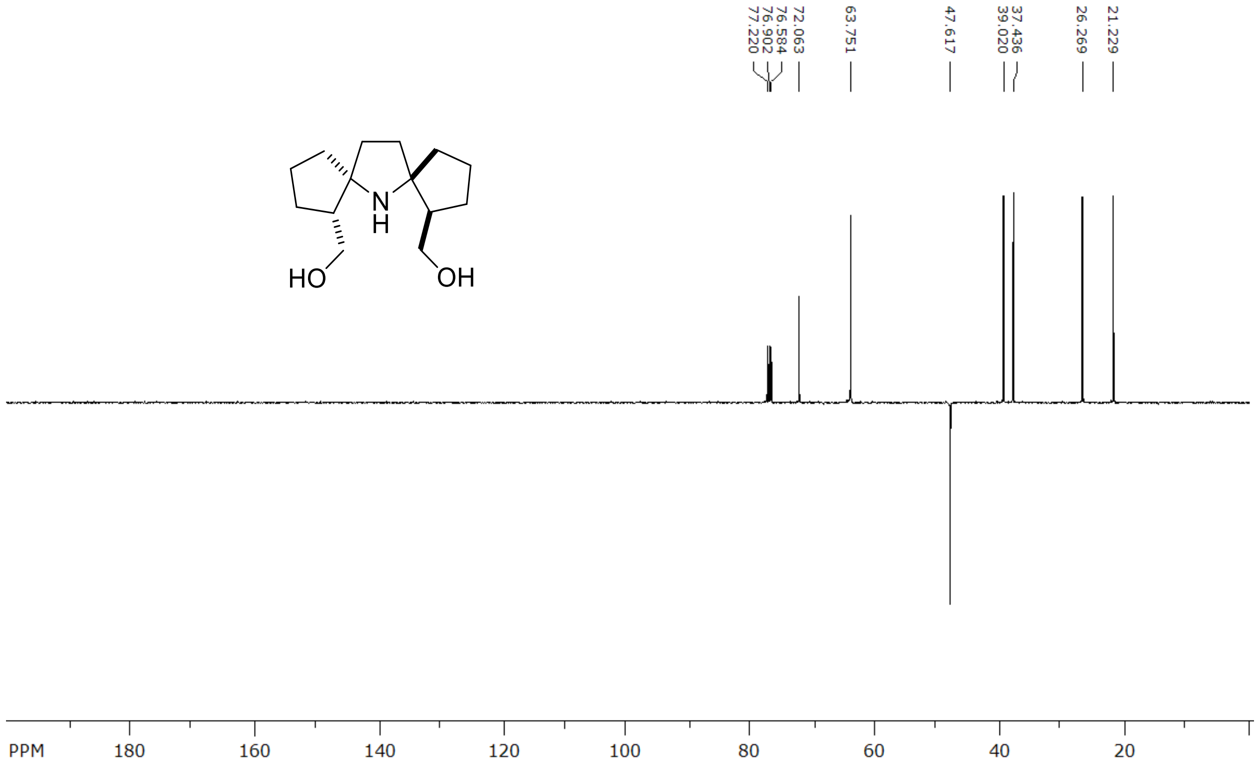

Fig. S38. ^{13}C NMR spectrum of (1*R*(*S*),5*R*(*S*),7*R*(*S*),8*R*(*S*))-6-azadispiro[4.1.4.2]tridecan-1,8-diyldimethanol (**14**) in $CDCl_3$ at 100 MHz

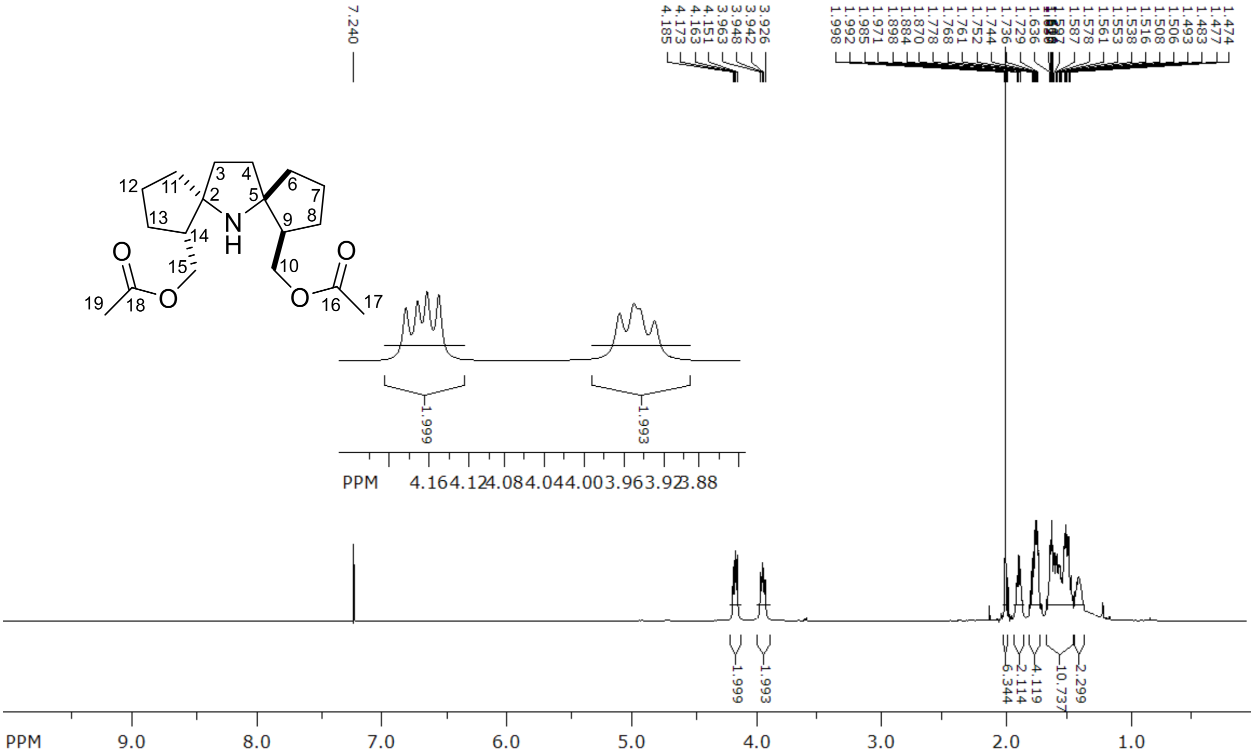

Fig. S39. ^{1}H NMR spectrum of (1*R*(*S*),5*R*(*S*),7*R*(*S*),8*R*(*S*))-6-azadispiro[4.1.4.2]tridecan-1,8-diyldi(methylene) diacetate (**16**) in $CDCl_3$ at 500 MHz

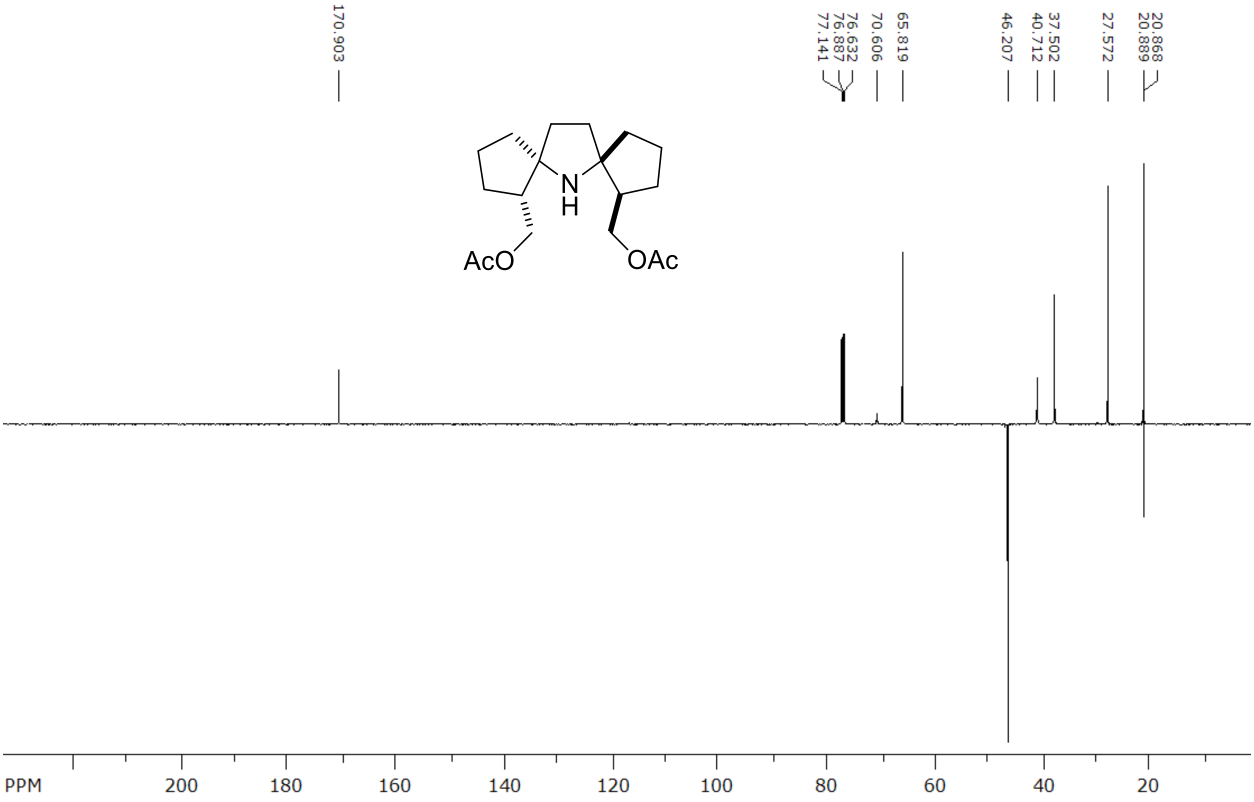

Fig. S40. ^{13}C NMR spectrum of (1*R*(*S*),5*R*(*S*),7*R*(*S*),8*R*(*S*))-6-azadispiro[4.1.4.2]tridecan-1,8-diyldi(methylene) diacetate (**16**) in $CDCl_3$ at 125 MHz

International Journal of *Molecular Sciences*

MDPI

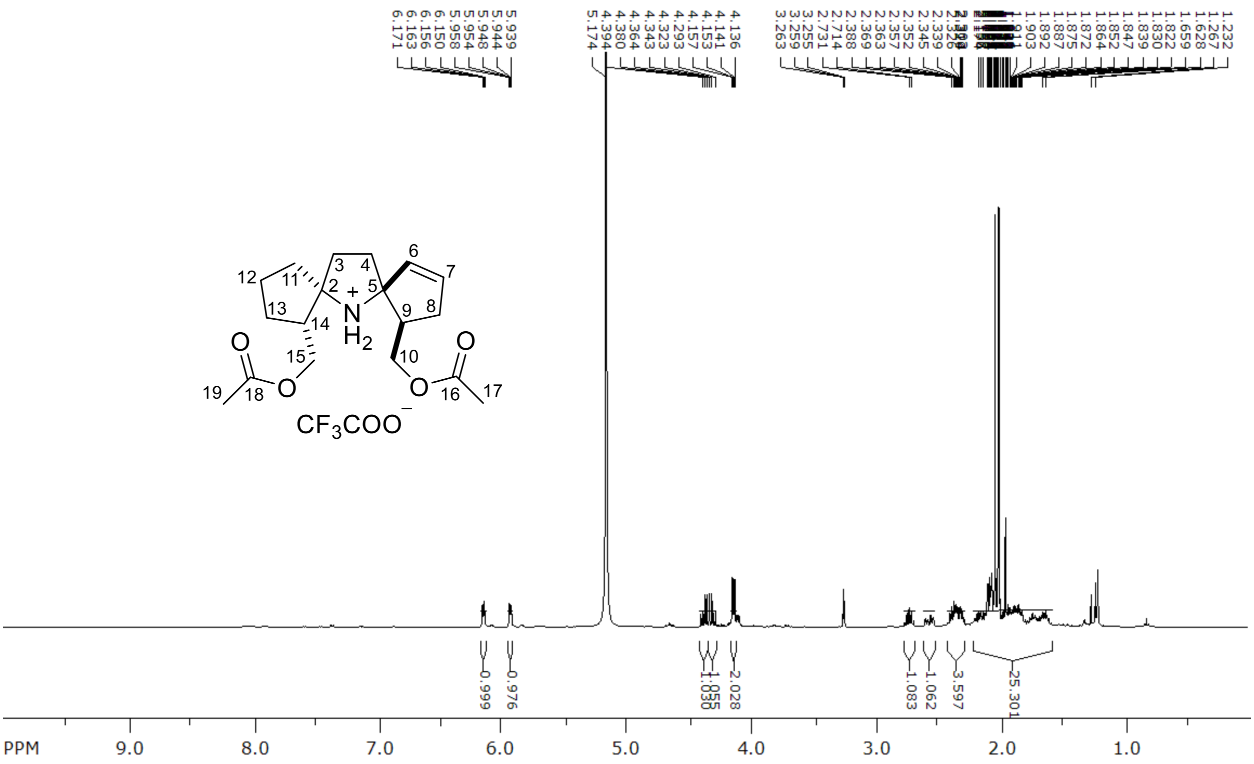

Fig. S41. ^{1}H NMR spectrum of (4*R*(*S*),5*S*(*R*),7*R*(*S*),8*R*(*S*))-4,8-Bis[(acetyloxy)methyl]-6-azoniadispiro[4.1.4.2]tridec-1-ene trifluoroacetate (**19**) in CD_3OD+CF_3COOH at 400 MHz

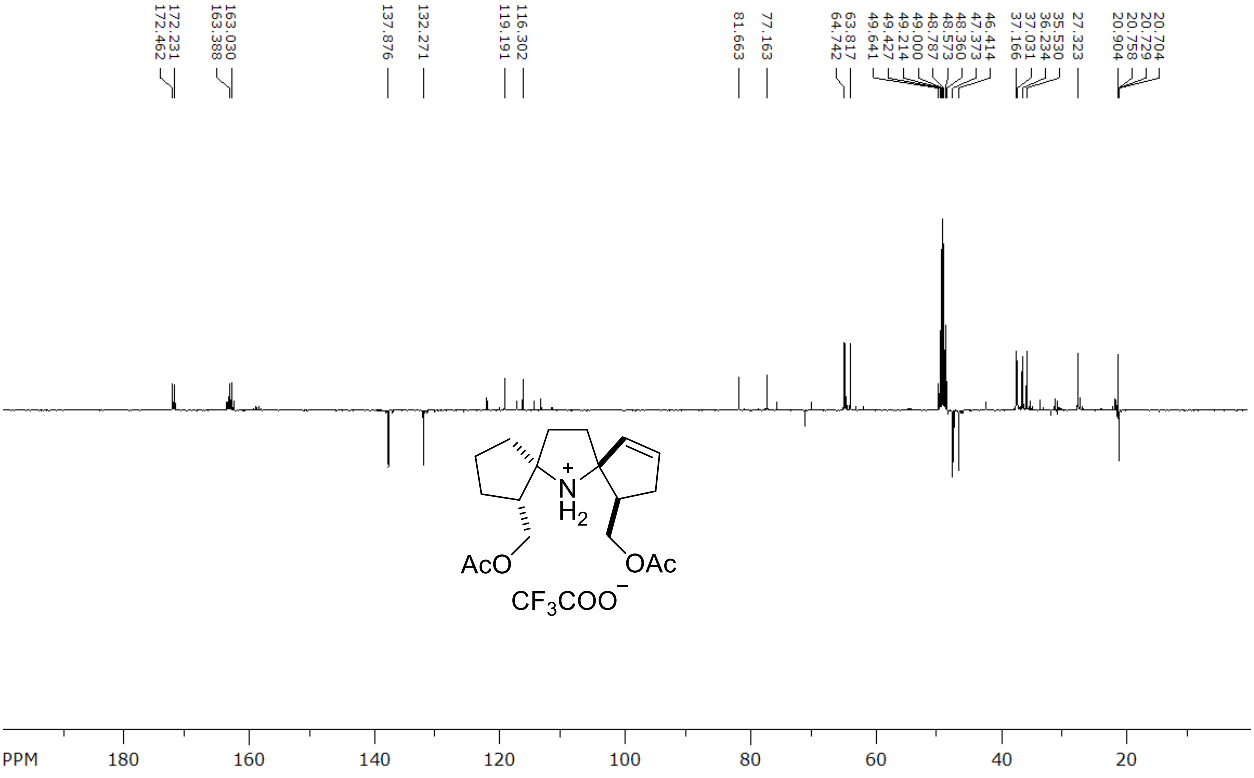

Fig. S42. ^{13}C NMR spectrum of (4*R*(*S*),5*S*(*R*),7*R*(*S*),8*R*(*S*))-4,8-Bis[(acetyloxy)methyl]-6-azoniadispiro[4.1.4.2]tridec-1-ene trifluoroacetate (**19**) in CD_3OD+CF_3COOH at 100 MHz

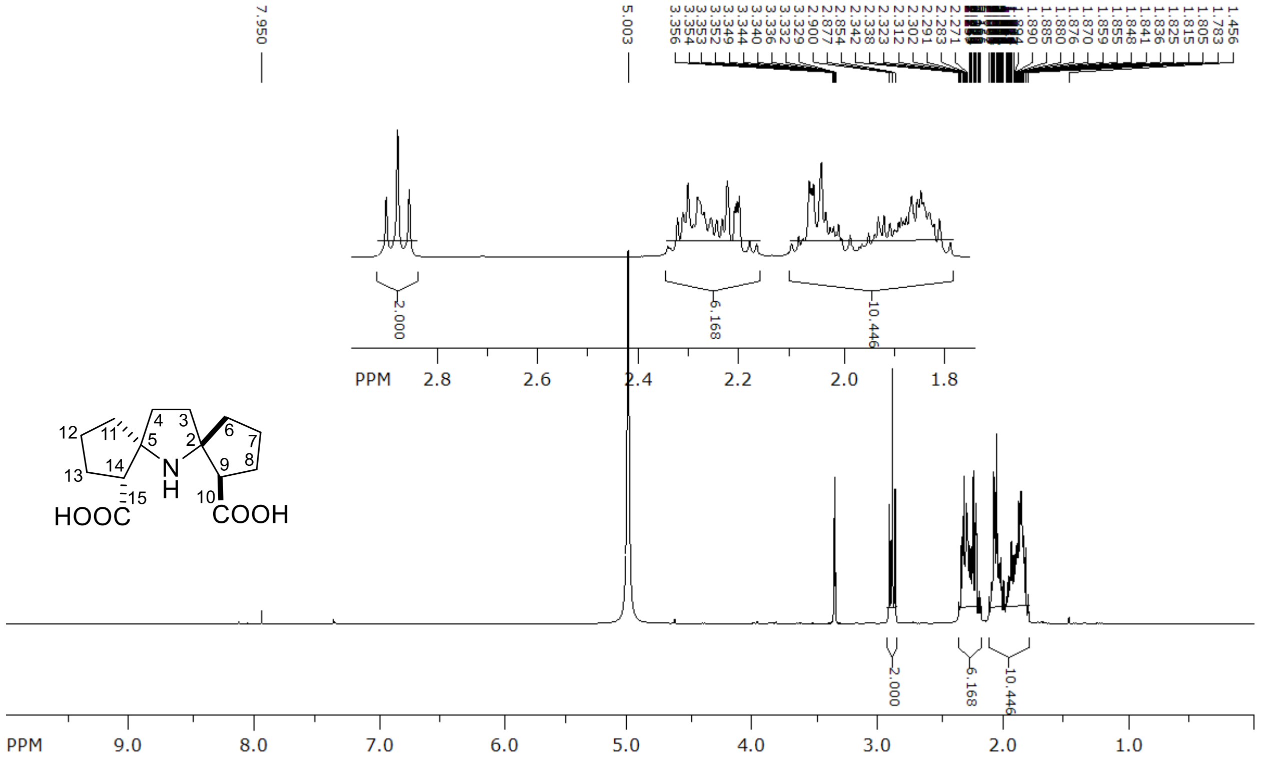

Fig. S43. ^{1}H NMR spectrum of (1*R*(*S*),5*R*(*S*),7*R*(*S*),8*R*(*S*))-6-Azadisporo[4.1.4.2]tridecan-1,8-dicarboxylic acid (**20**) in CD_3OD at 400 MHz

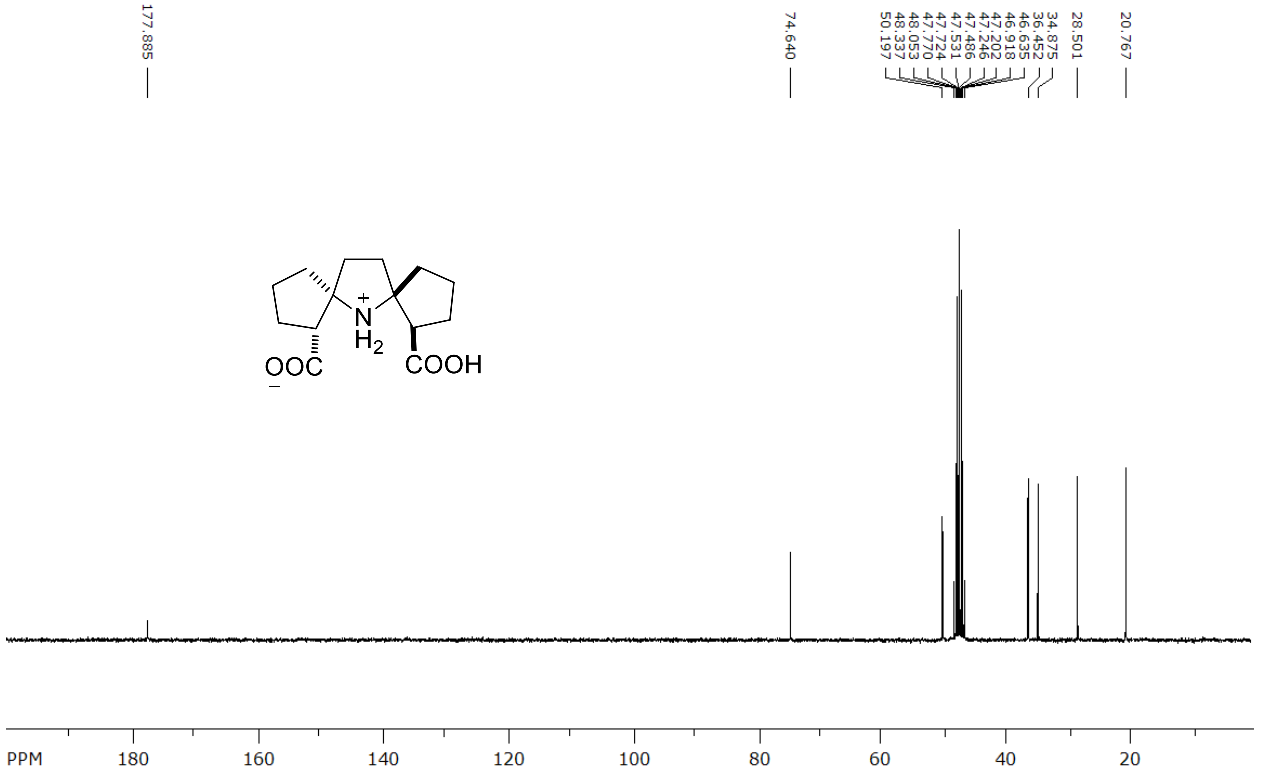

Fig. S44. ^{1}H NMR spectrum of (1*R*(*S*),5*R*(*S*),7*R*(*S*),8*R*(*S*))-6-Azadisporo[4.1.4.2]tridecan-1,8-dicarboxylic acid (**20**) in CD_3OD at 75 MHz

International Journal of
Molecular Sciences

MDPI

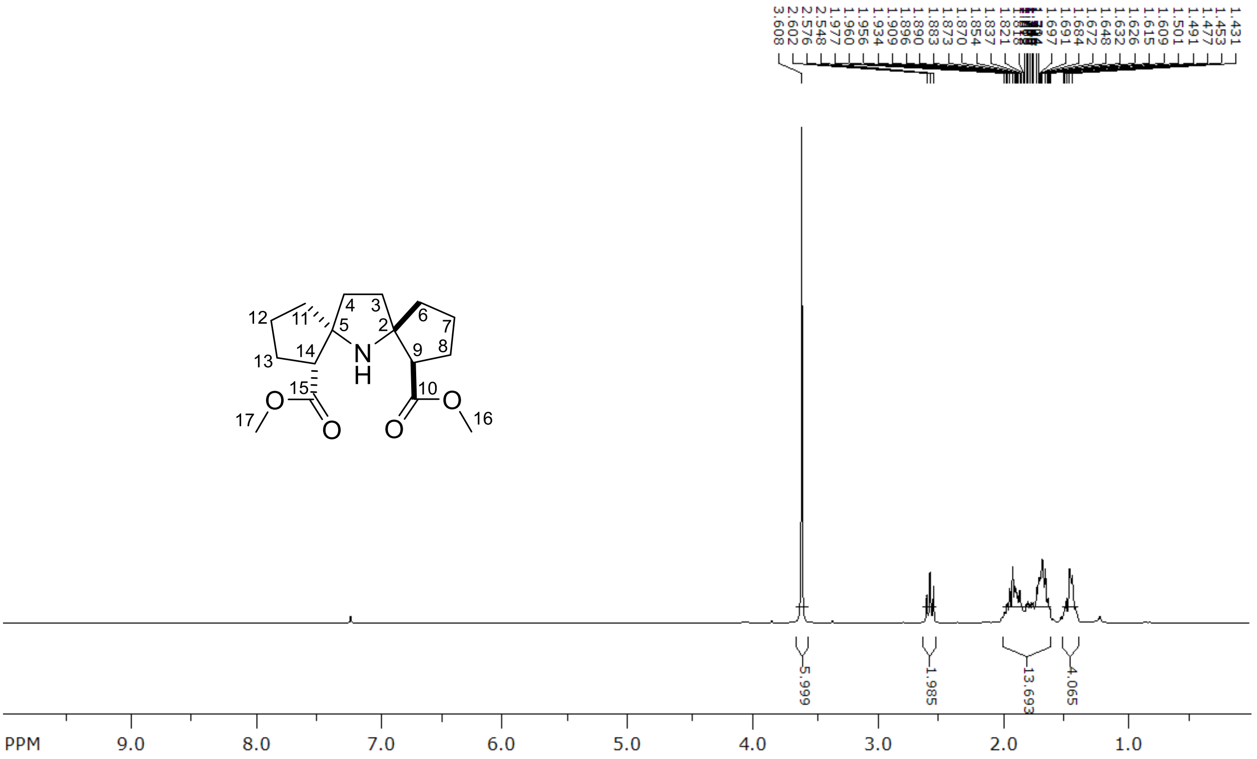

Fig. S45. ^{1}H NMR spectrum of Dimethyl (1*R*(*S*),5*R*(*S*),7*R*(*S*),8*R*(*S*))-6-azadispiro[4.1.4.2]tridecan-1,8-dicarboxylate (**21**) in $CDCl_3$ at 300 MHz

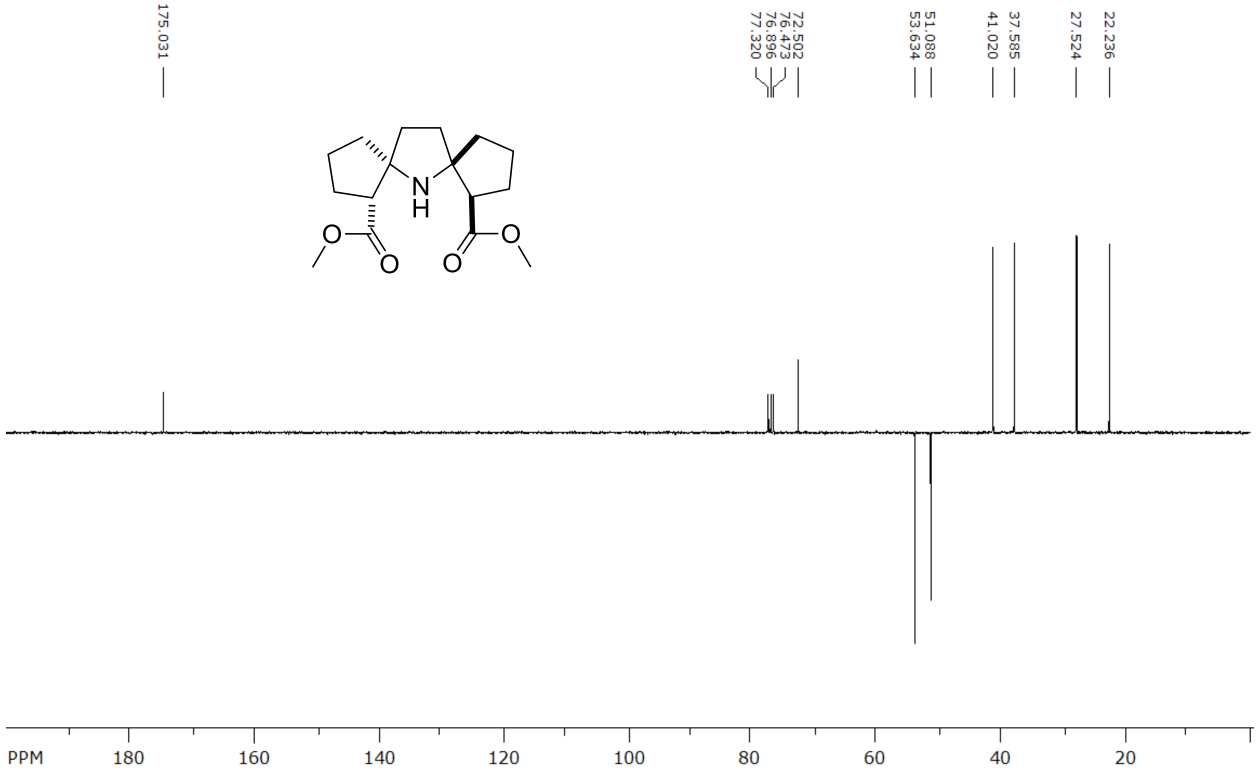

Fig. S46. ^{1}H NMR spectrum of Dimethyl (1*R*(*S*),5*R*(*S*),7*R*(*S*),8*R*(*S*))-6-azadispiro[4.1.4.2]tridecan-1,8-dicarboxylate (**21**) in $CDCl_3$ at 75 MHz

International Journal of
Molecular Sciences
MDPI

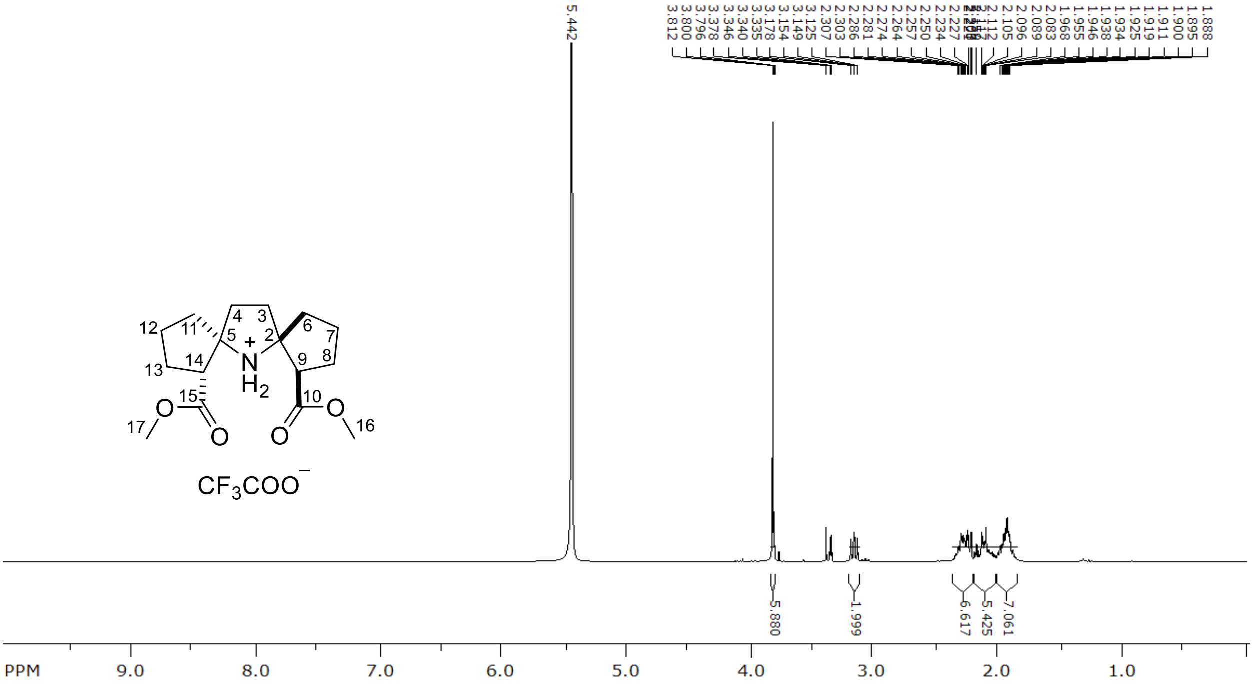

Fig. S47. ¹H NMR spectrum of (1*R*(*S*),5*R*(*S*),7*R*(*S*),8*R*(*S*))-1,8-Bis(methoxycarbonyl)-6-azoniadispiro[4.1.4.2]tridecane trifluoroacetate (**22-red**) in CD_3OD+CF_3COOH at 300 MHz

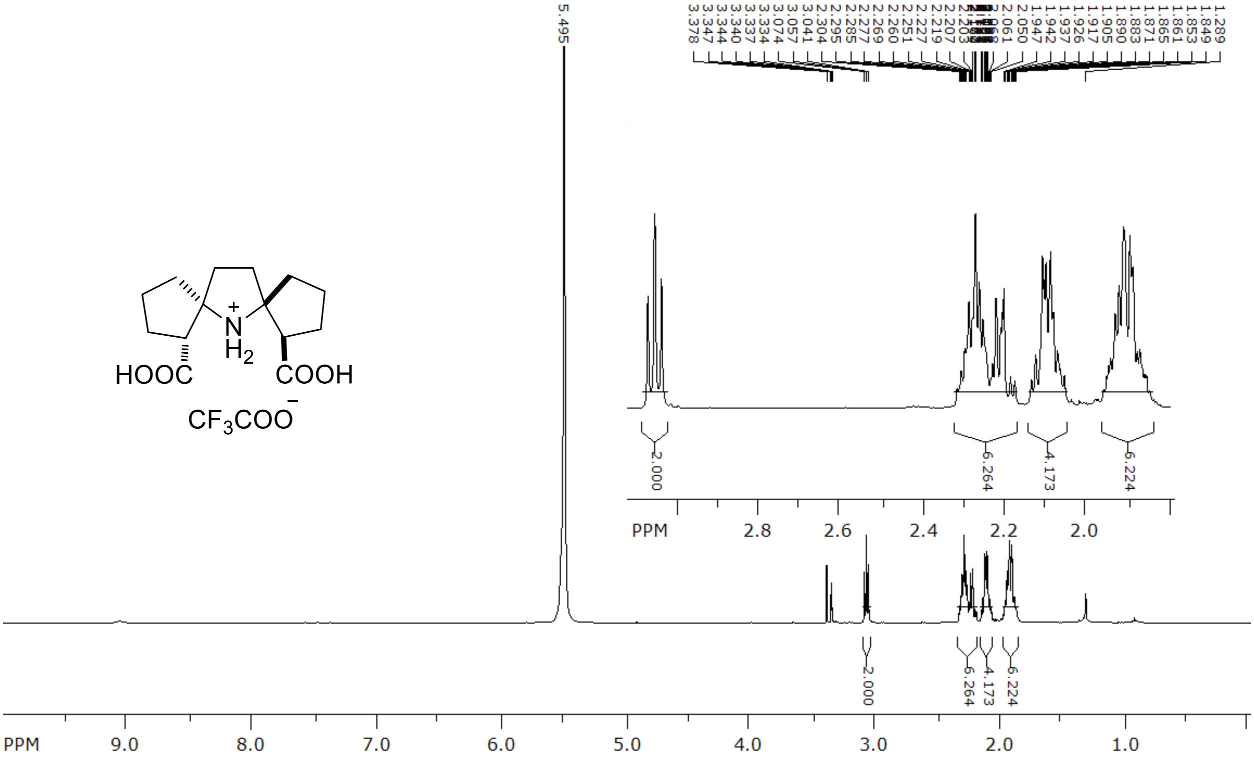

Fig. S48. ¹H NMR spectrum of (1*R*(*S*),5*R*(*S*),7*R*(*S*),8*R*(*S*))-1,8-Dicarboxy-6-azoniadispiro[4.1.4.2]tridecane trifluoroacetate (**23a-red**) in CD_3OD+CF_3COOH at 500 MHz

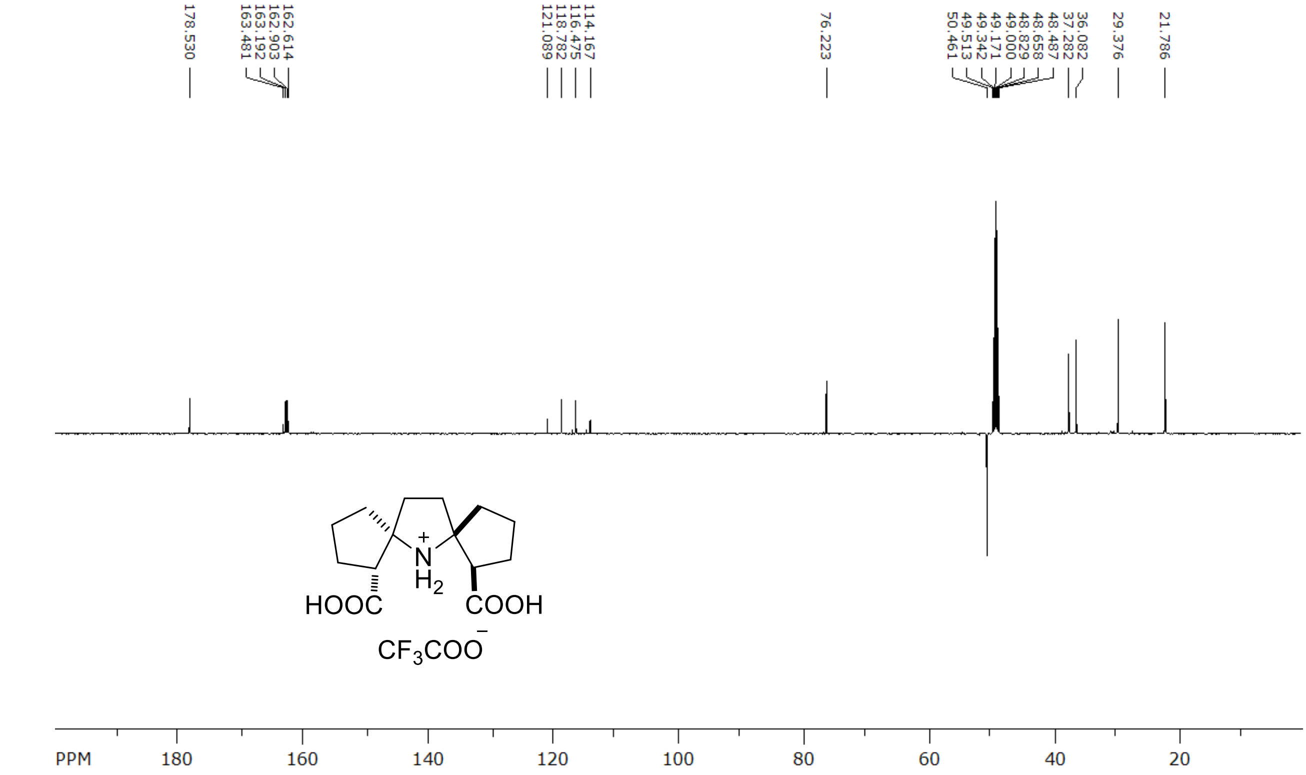

Fig. S49. ^{13}C NMR spectrum of (1*R*(*S*),5*R*(*S*),7*R*(*S*),8*R*(*S*))-1,8-Dicarboxy-6-azoniadispiro[4.1.4.2]tridecane trifluoroacetate (**23a-red**) in CD_3OD+CF_3COOH at 125 MHz

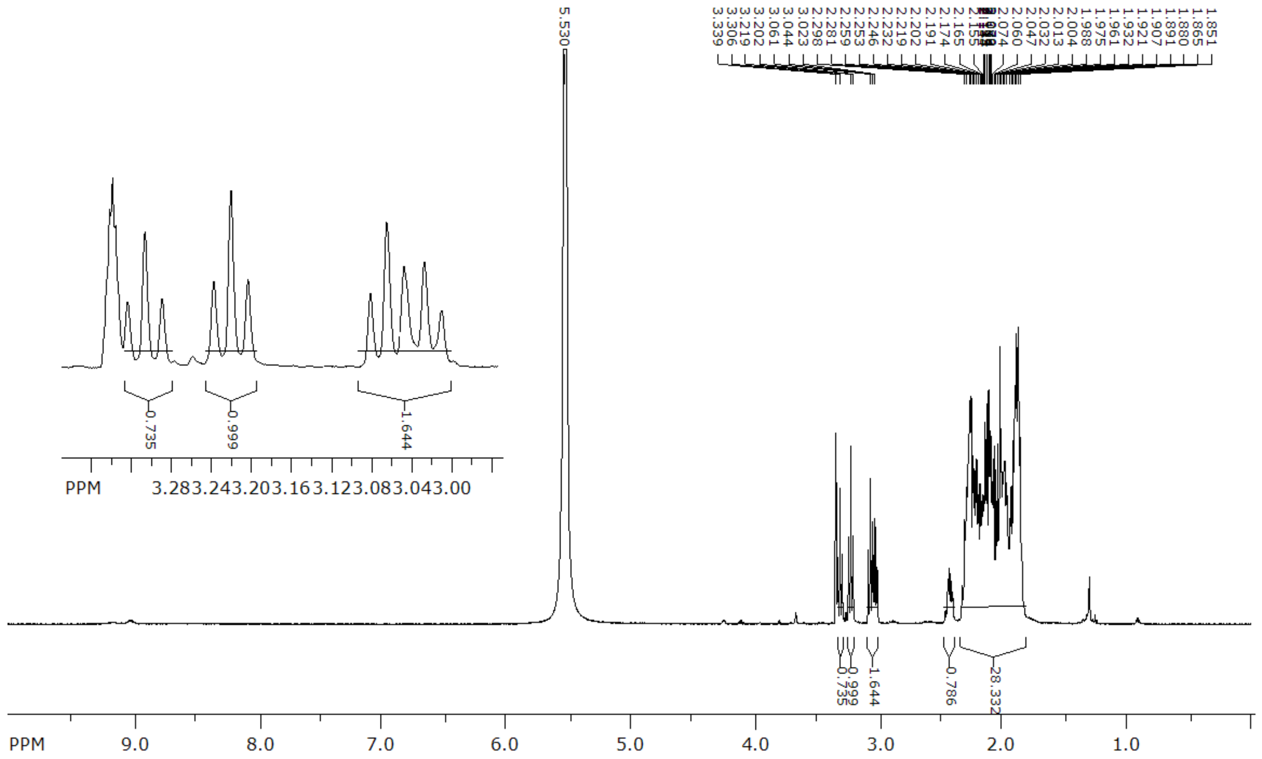

Fig. S50. ^{1}H NMR spectrum of isomeric mixture of 1,8-Dicarboxy-6-azoniadispiro[4.1.4.2]tridecane trifluoroacetates (**23a+23b+23c-red**) in CD_3OD+CF_3COOH at 500 MHz

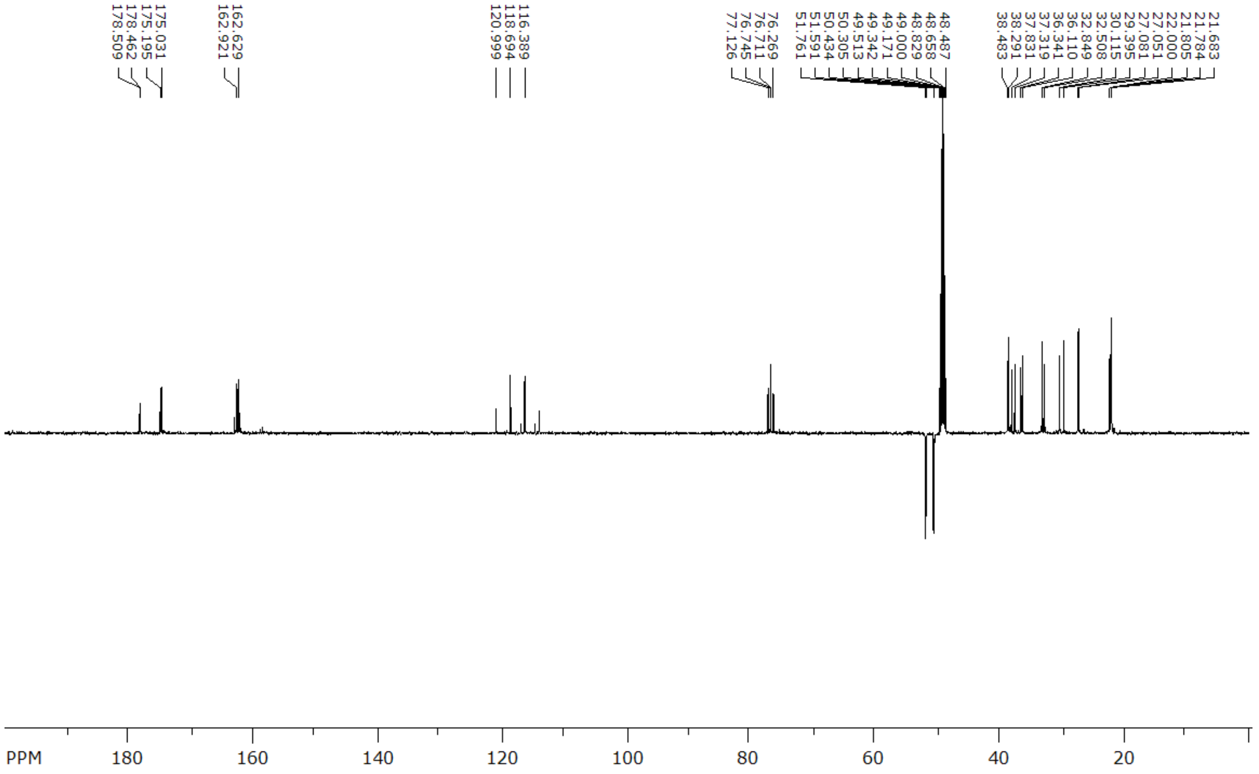

Fig. S51. ^{13}C NMR spectrum of isomeric mixture of 1,8-Dicarboxy-6-azoniadispiro[4.1.4.2]tridecane trifluoroacetates (**23a+23b+23c-red**) in CD_3OD+CF_3COOH at 125 MHz

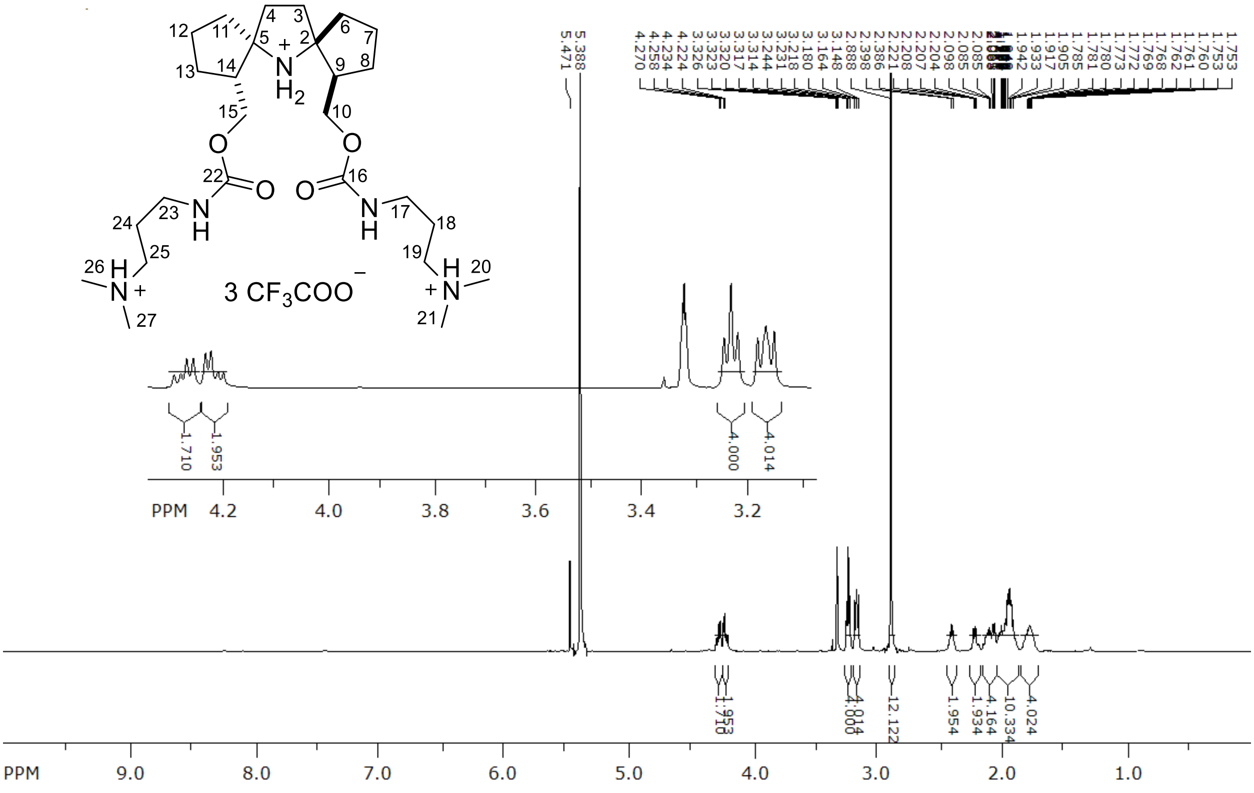

Fig. S52. ^1H NMR spectrum of (1R(S),5R(S),7R(S),8R(S))-1,8-Bis[(([{3-(dimethylamino)propyl}amino]carbonyl)oxy)methyl]-6-azoniadispiro[4.1.4.2]tridecane trifluoroacetate (**27-red**) in CD_3OD+CF_3COOD at 500 MHz

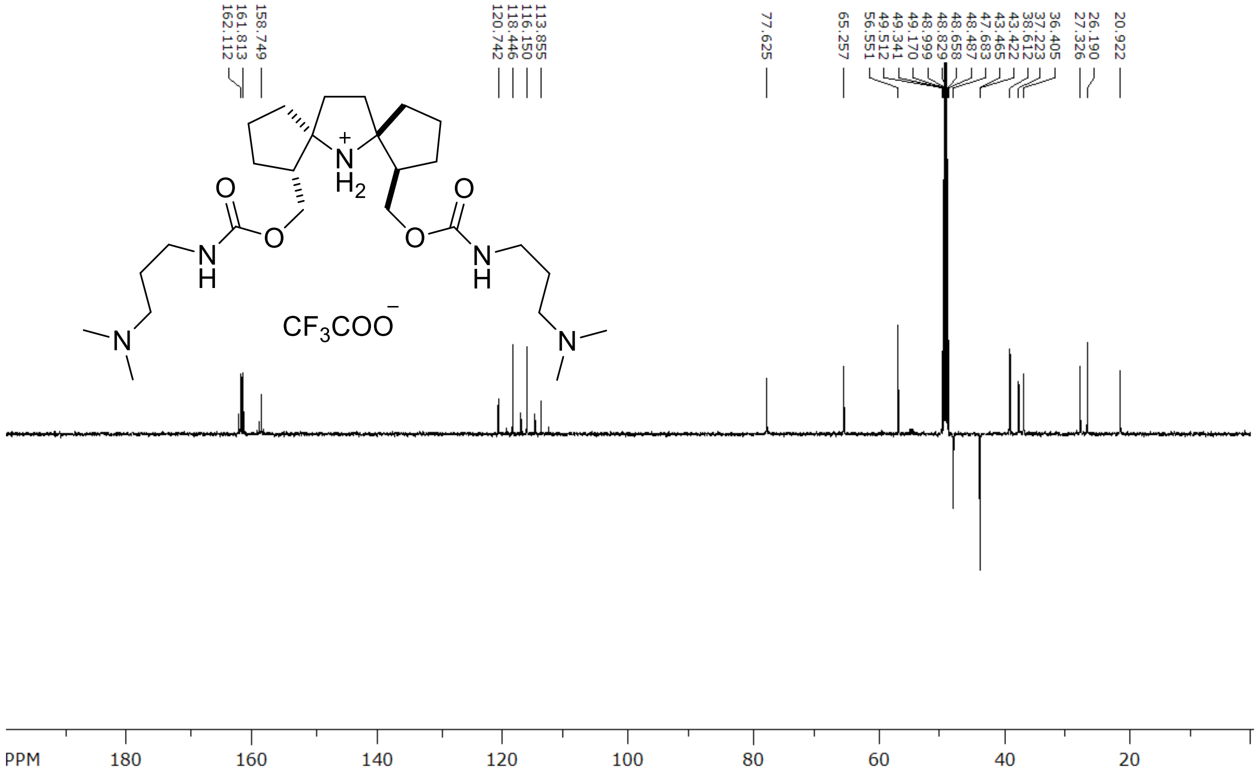

Fig. S53. ^{13}C NMR spectrum of (1R(S),5R(S),7R(S),8R(S))-1,8-Bis[((([{3-(dimethylamino)propyl}amino]carbonyl)oxy)methyl]-6-azoniadispiro[4.1.4.2]tridecane trifluoroacetate (**27-red**) in CD_3OD+CF_3COOD at 125 MHz

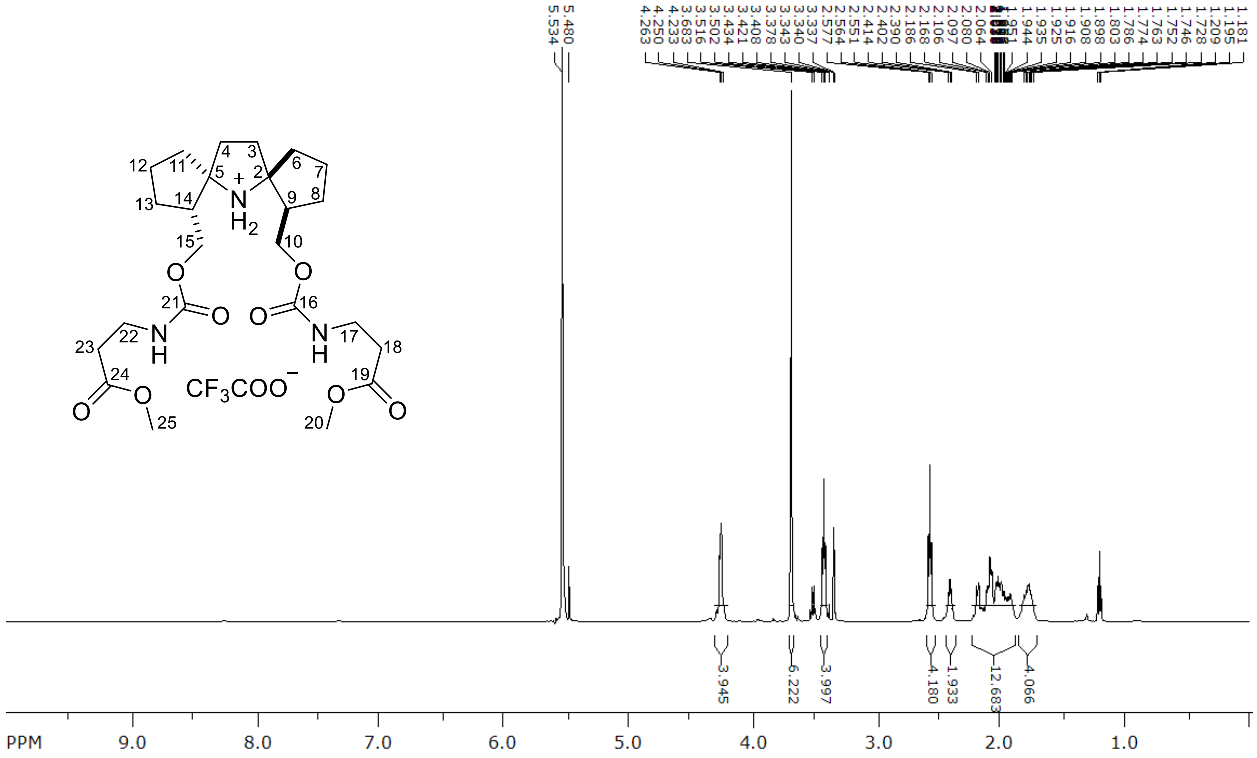

Fig. S54. ^1H NMR spectrum of (1*R*(*S*),5*R*(*S*),7*R*(*S*),8*R*(*S*))-1,8-Bis[((([{3-methoxy-3-oxopropyl}amino]carbonyl)oxy)methyl]-6-azoniadispiro[4.1.4.2]tridecane trifluoroacetate (**28-red**) in CD_3OD+CF_3COOD at 500 MHz

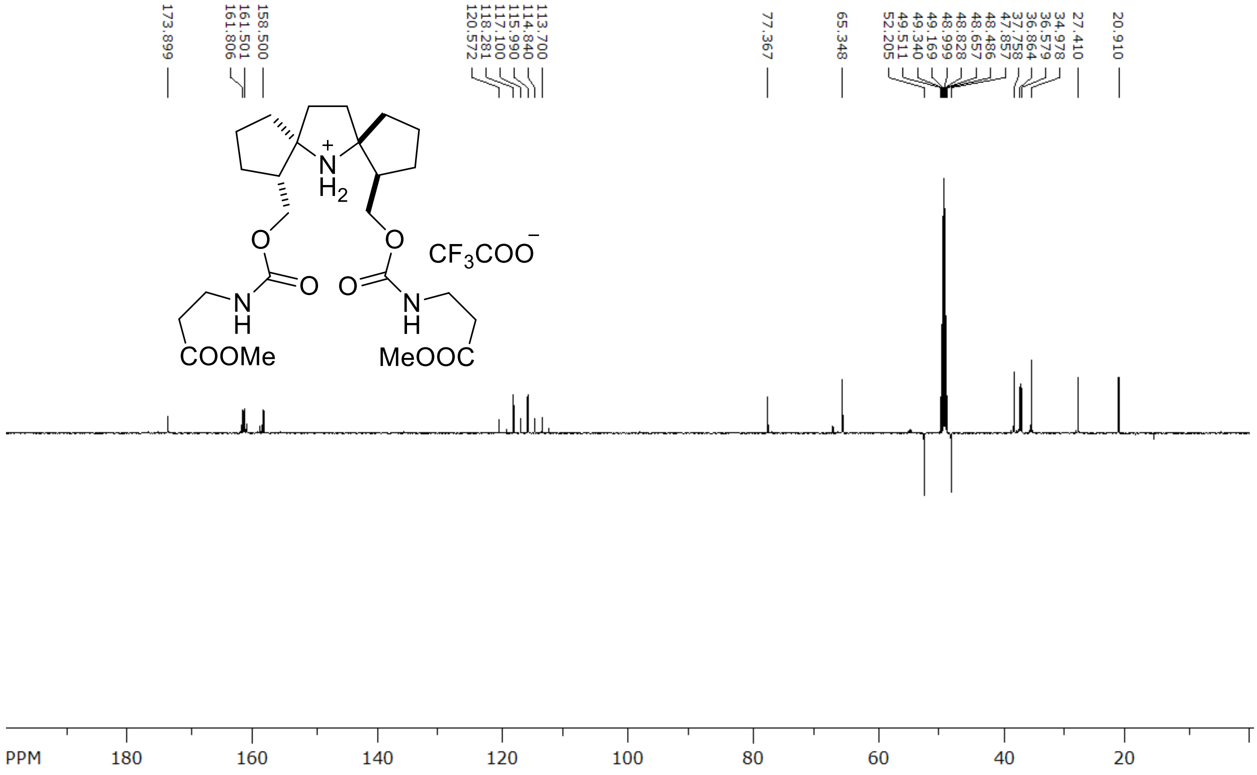

Fig. S55. ^{13}C NMR spectrum of (1*R*(*S*),5*R*(*S*),7*R*(*S*),8*R*(*S*))-1,8-Bis[((([{3-methoxy-3-oxopropyl}amino]carbonyl)oxy)methyl]-6-azoniadispiro[4.1.4.2]tridecane trifluoroacetate (**28-red**) in CD_3OD+CF_3COOD at 125 MHz

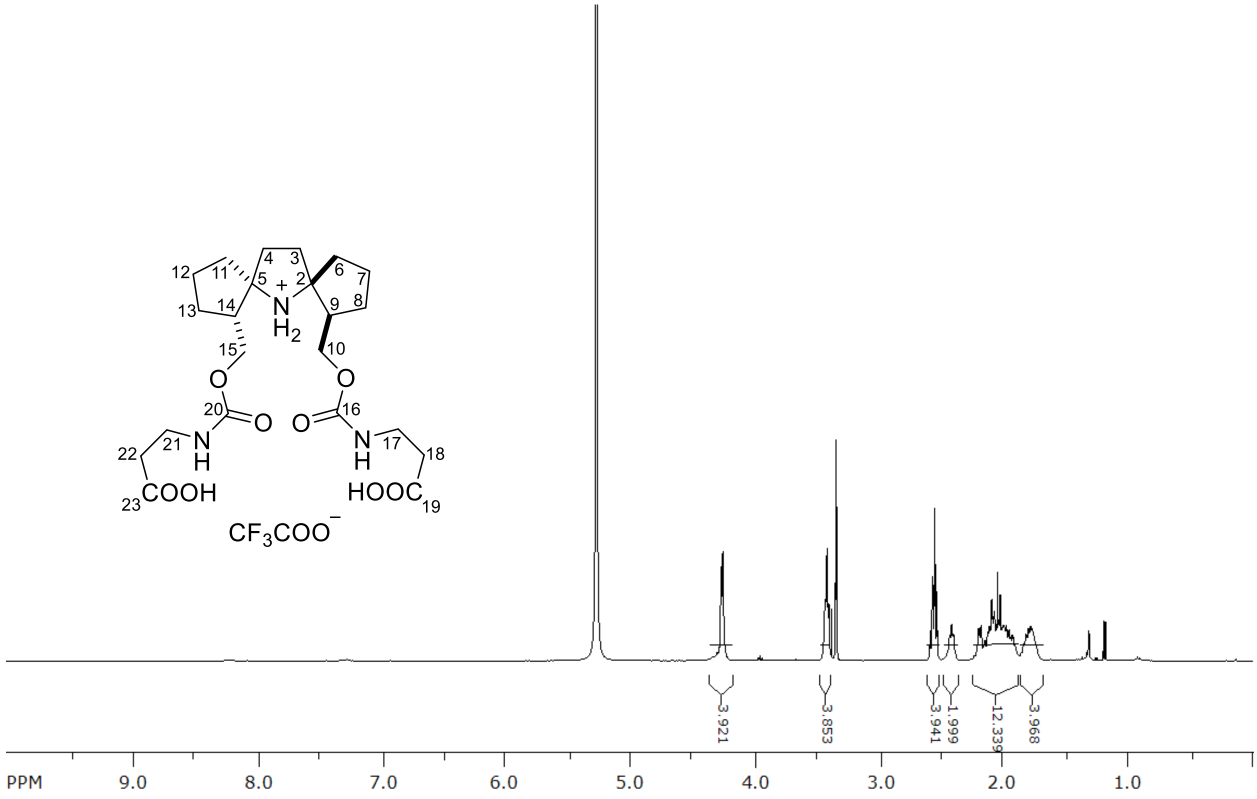

Fig. S56. ^{1}H NMR spectrum of (1*R*(*S*),5*R*(*S*),7*R*(*S*),8*R*(*S*))-1,8-Bis[((([{2-carboxyethyl}amino]carbonyl)oxy)methyl]-6-azoniadispiro[4.1.4.2]tridecane trifluoroacetate (**29-red**) in CD_3OD+CF_3COOD at 400 MHz

International Journal of *Molecular Sciences*

MDPI

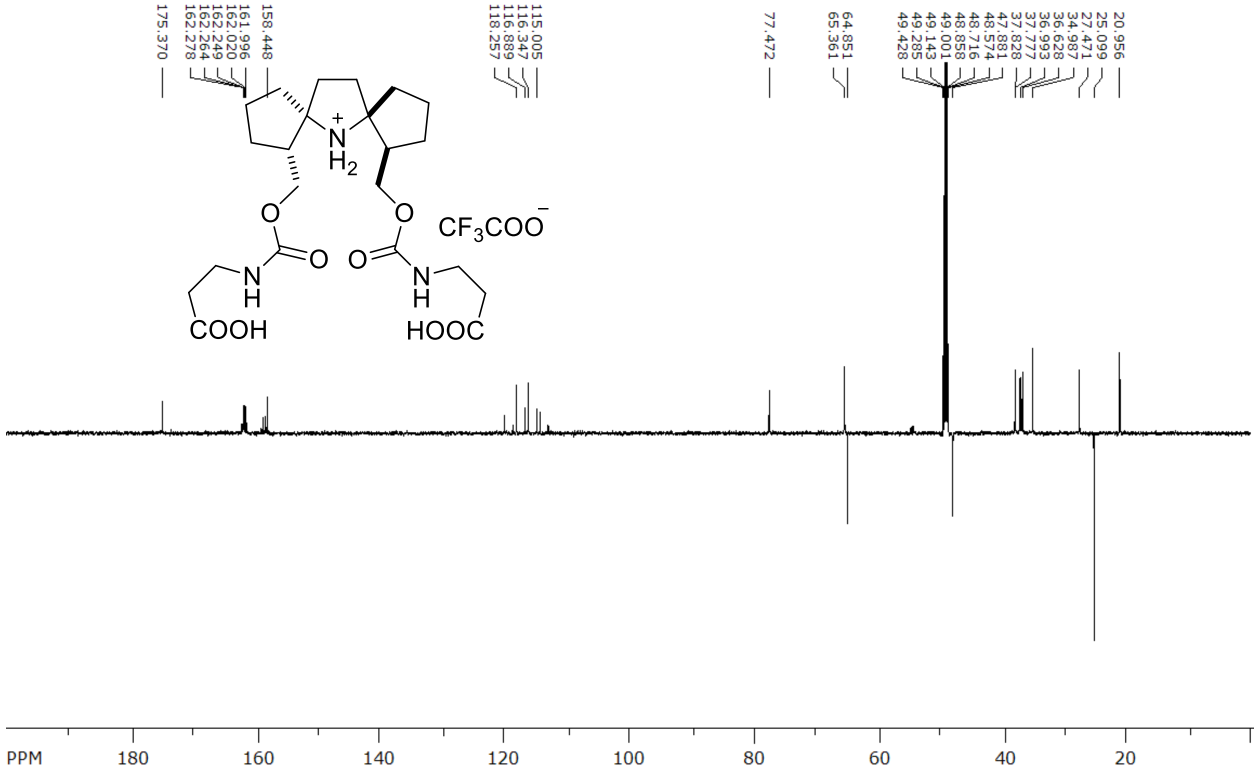

Fig. S57. ^{13}C NMR spectrum of (1*R*(*S*),5*R*(*S*),7*R*(*S*),8*R*(*S*))-1,8-Bis[((([{2-carboxyethyl}amino]carbonyl)oxy)methyl]-6-azoniadispiro[4.1.4.2]tridecane trifluoroacetate (**29-red**) in CD_3OD+CF_3COOD at 150 MHz

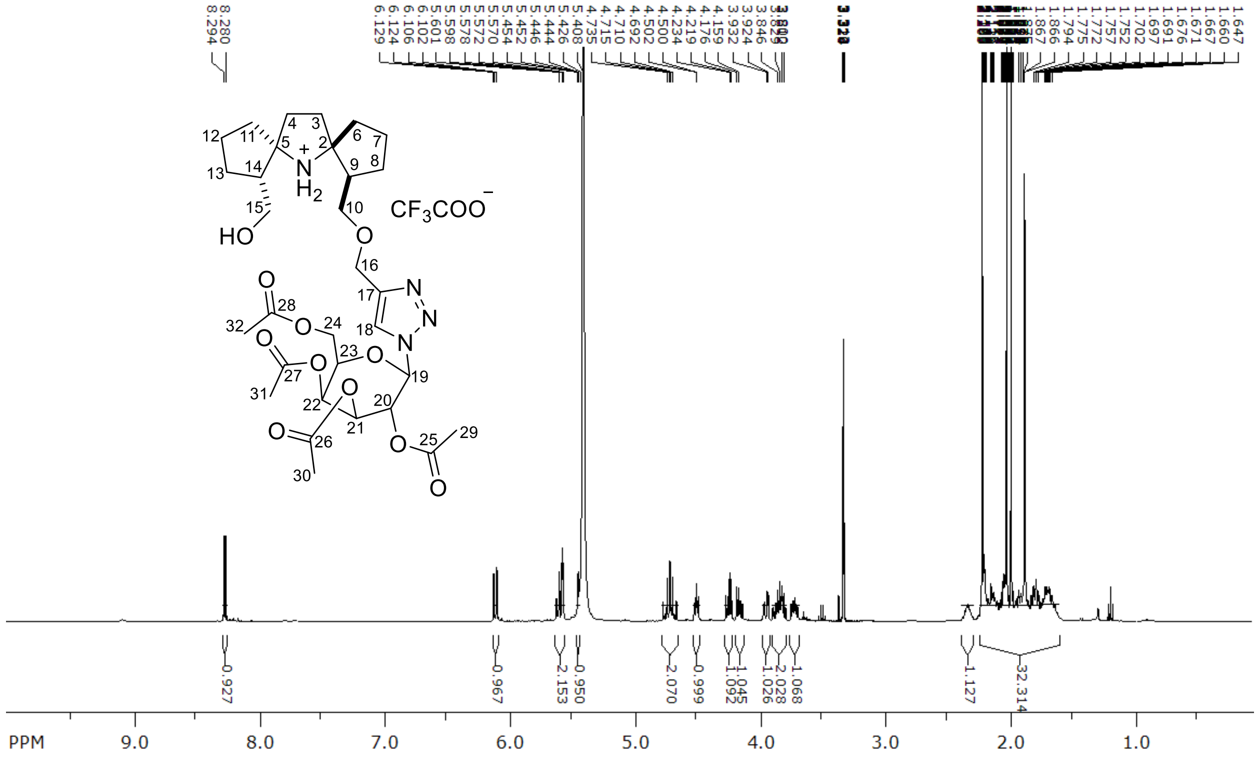

Fig. S58. ^{1}H NMR spectrum of (1*R*(*S*),5*R*(*S*),7*R*(*S*),8*R*(*S*))-1-(Hydroxymethyl)-8-({(1-[2,3,4,6-tetra-*O*-acetyl-β-D-galactopyranosyl]-1*H*-1,2,3-triazol-4-yl)methoxy}methyl)-6-azoniadispiro[4.1.4.2]tridecane trifluoroacetate (**32-red**) in CD_3OD+CF_3COOH at 400 MHz

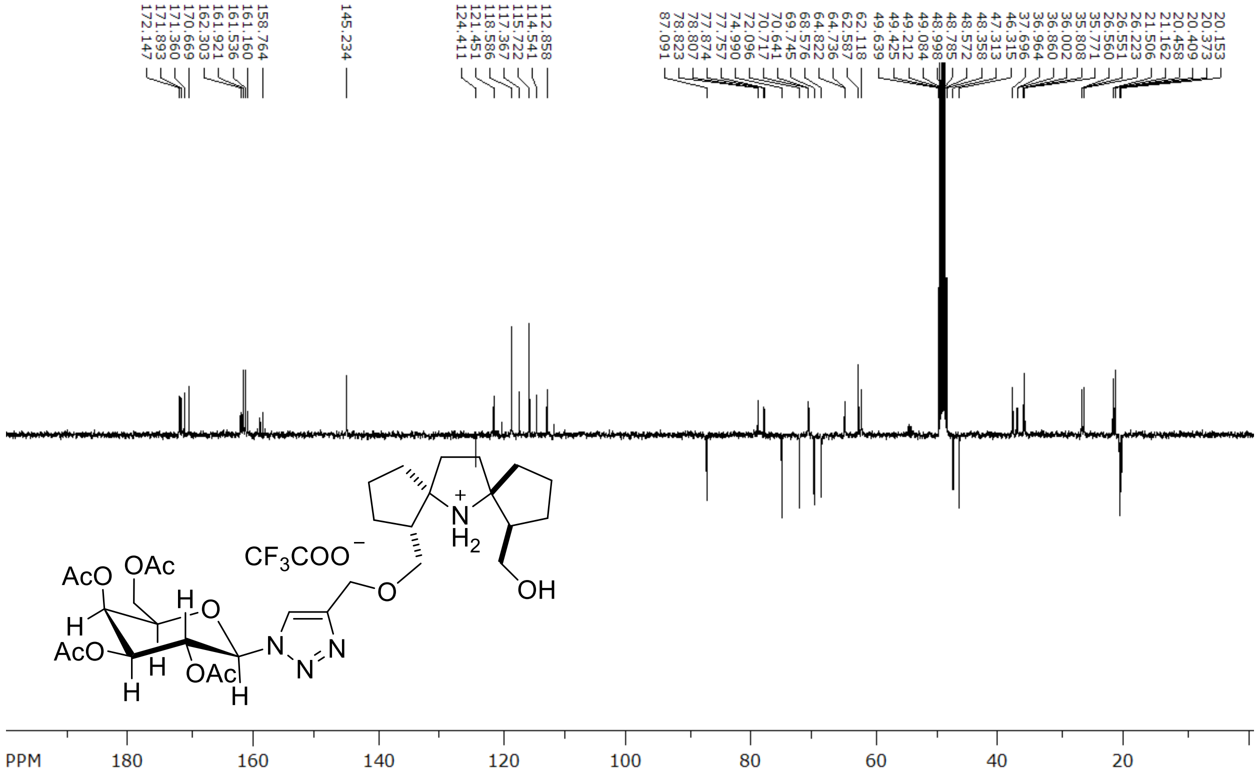

Fig. S59. ^{13}C NMR spectrum of (1*R*(*S*),5*R*(*S*),7*R*(*S*),8*R*(*S*))-1-(Hydroxymethyl)-8-({(1-[2,3,4,6-tetra-*O*-acetyl-β-D-galactopyranosyl]-1*H*-1,2,3-triazol-4-yl)methoxy}methyl)-6-azoniadispiro[4.1.4.2]tridecane trifluoroacetate (**32-red**) in CD_3OD+CF_3COOD at 100 MHz

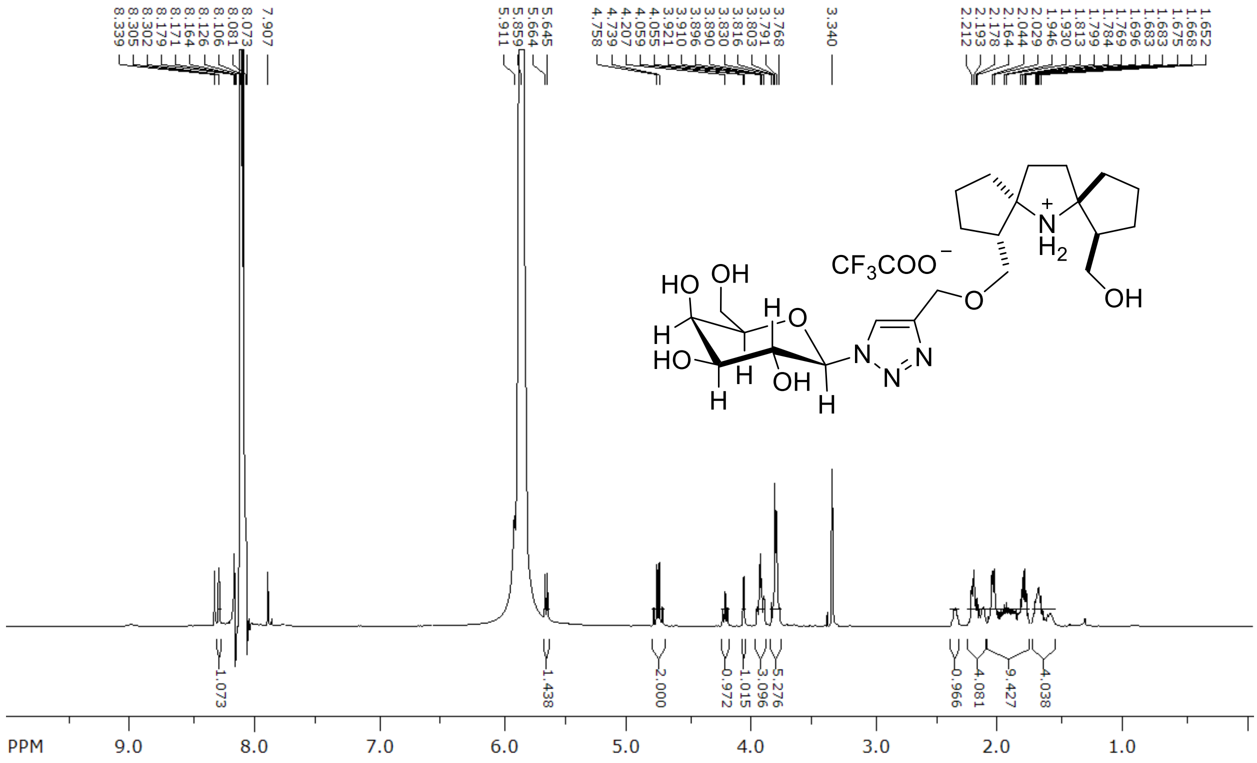

Fig. S60. ^{1}H NMR spectrum of (1*R*(*S*),5*R*(*S*),7*R*(*S*),8*R*(*S*))-1-(Hydroxymethyl)-8-({(1-[β-D-galactopyranosyl]-1*H*-1,2,3-triazol-4-yl)methoxy}methyl)-6-azoniadispiro[4.1.4.2]tridecane trifluoroacetate (**33-red)** in CD_3OD+HCOOH+HOOCCOOH at 500 MHz

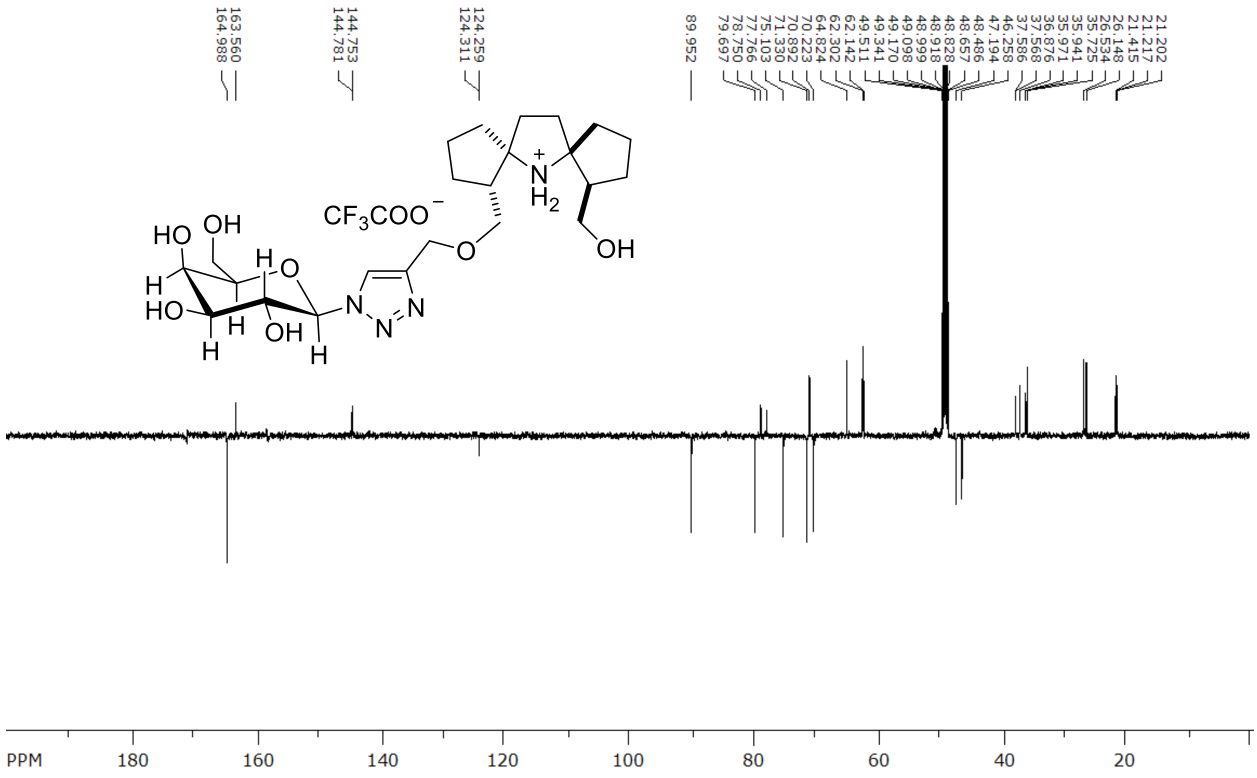

Fig. S61. ^{13}C NMR spectrum of (1*R*(*S*),5*R*(*S*),7*R*(*S*),8*R*(*S*))-1-(Hydroxymethyl)-8-({(1-[β-D-galactopyranosyl]-1*H*-1,2,3-triazol-4-yl)methoxy}methyl)-6-azoniadispiro[4.1.4.2]tridecane trifluoroacetate (**33-red)** in CD_3OD+HCOOH+HOOCCOOH at 125 MHz

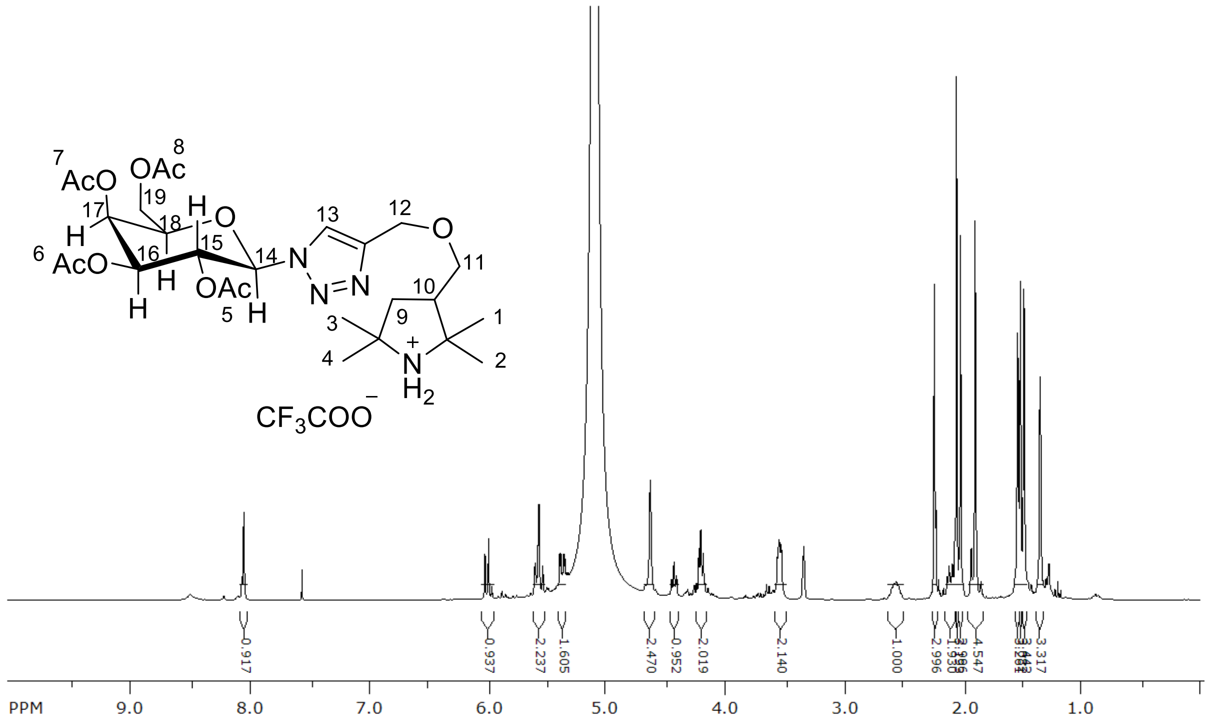

Fig. S62. ^{1}H NMR spectrum of 2,2,5,5-Tetramethyl-3-(((1-(2,3,4,6-tetra-O-acetyl-β-D-galactopyranosyl)-1*H*-1,2,3-triazol-4-yl)methoxy)methyl)pyrrolidinium trifluoroacetate (**38-red)** in CD_3OD+CF_3COOH at 300 MHz

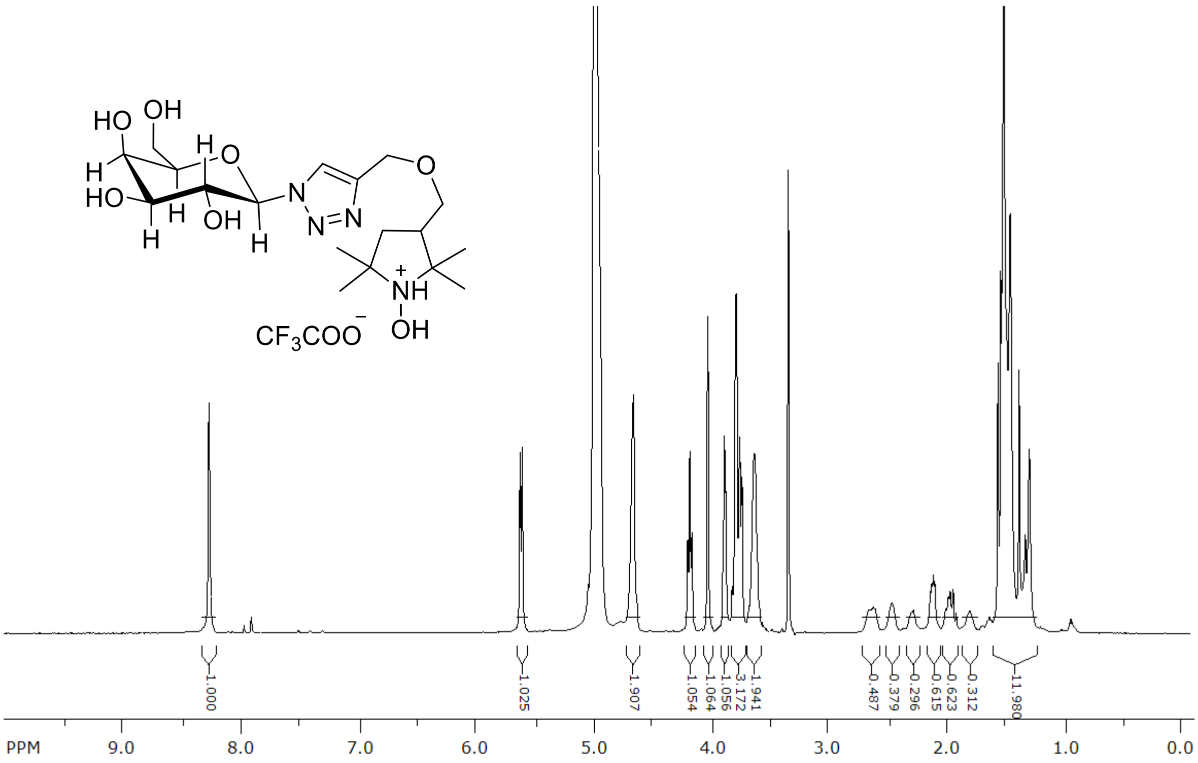

Fig. S63. ^{1}H NMR spectrum of 1-Hydroxy-2,2,5,5-tetramethyl-3-(((1-(β-D-galactopyranosyl)-1*H*-1,2,3-triazol-4-yl)methoxy)methyl)pyrrolidinium trifluoroacetate (**35-red)** in CD_3OD+CF_3COOH at 500 MHz

International Journal of Molecular Sciences

5. 2D NMR spectral data

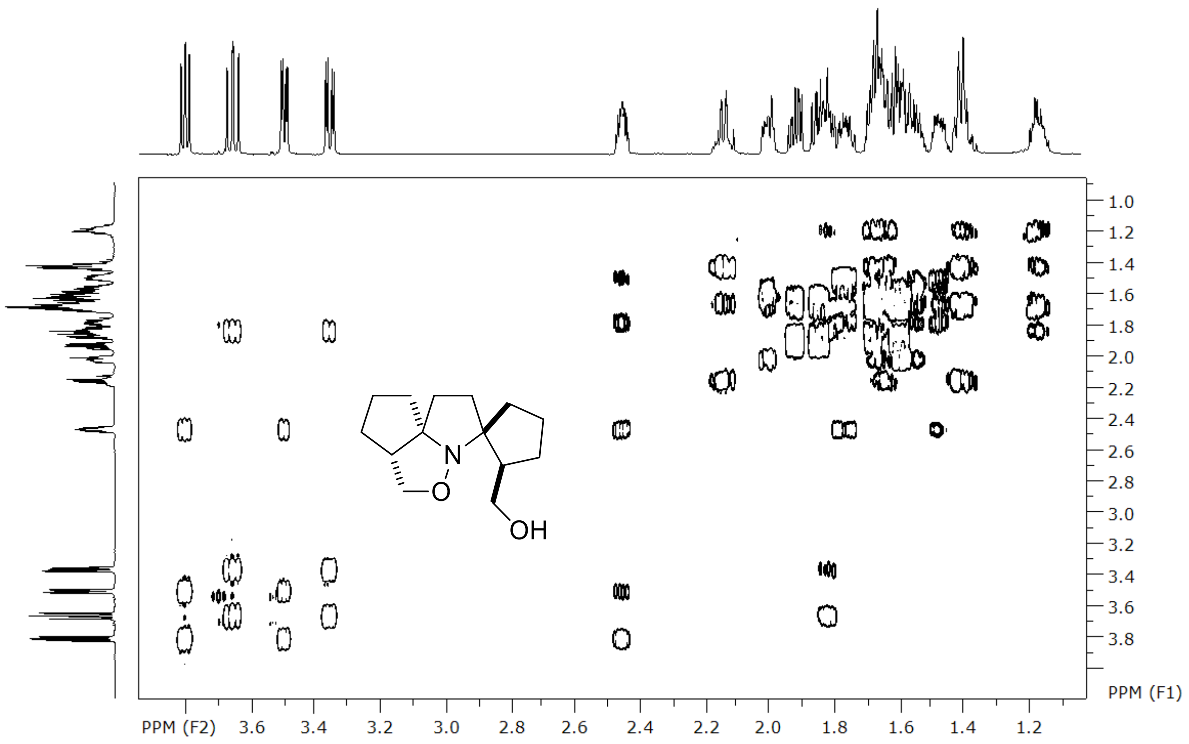

Fig. S64. ^{1}H-^{1}H COSY NMR spectrum of (1*R*(*S*),2*R*(*S*),6a′*R*(*S*),9a′*R*(*S*)-Hexahydro-6′*H*-spiro[cyclopentan-1,3′-cyclopenta[*c*]pyrrolo[1,2-*b*]isoxazol]-2-ylmethanol (**13**) in $CDCl_3$.

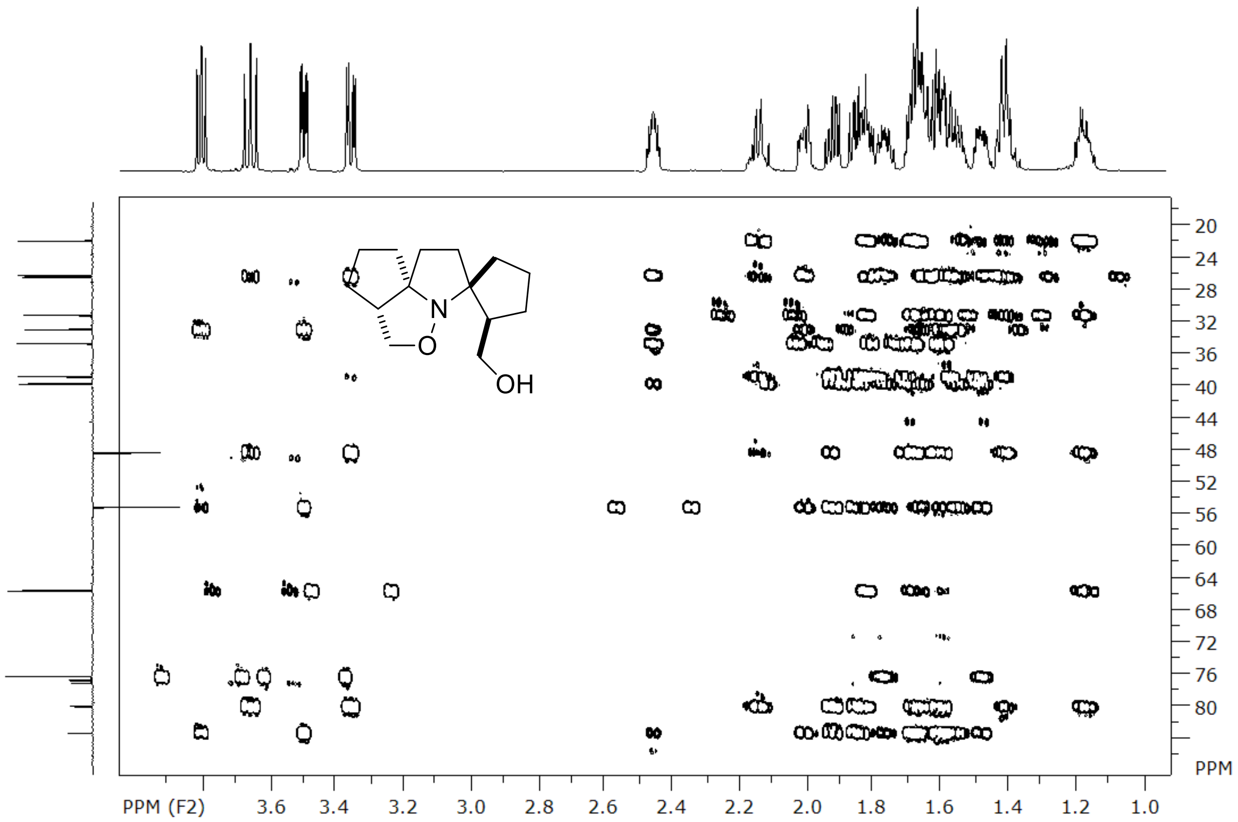

Fig. S65. ^{1}H-^{13}C HMBC NMR spectrum of (1*R*(*S*),2*R*(*S*),6a′*R*(*S*),9a′*R*(*S*)-Hexahydro-6′*H*-spiro[cyclopentan-1,3′-cyclopenta[*c*]pyrrolo[1,2-*b*]isoxazol]-2-ylmethanol (**13**) in $CDCl_3$.

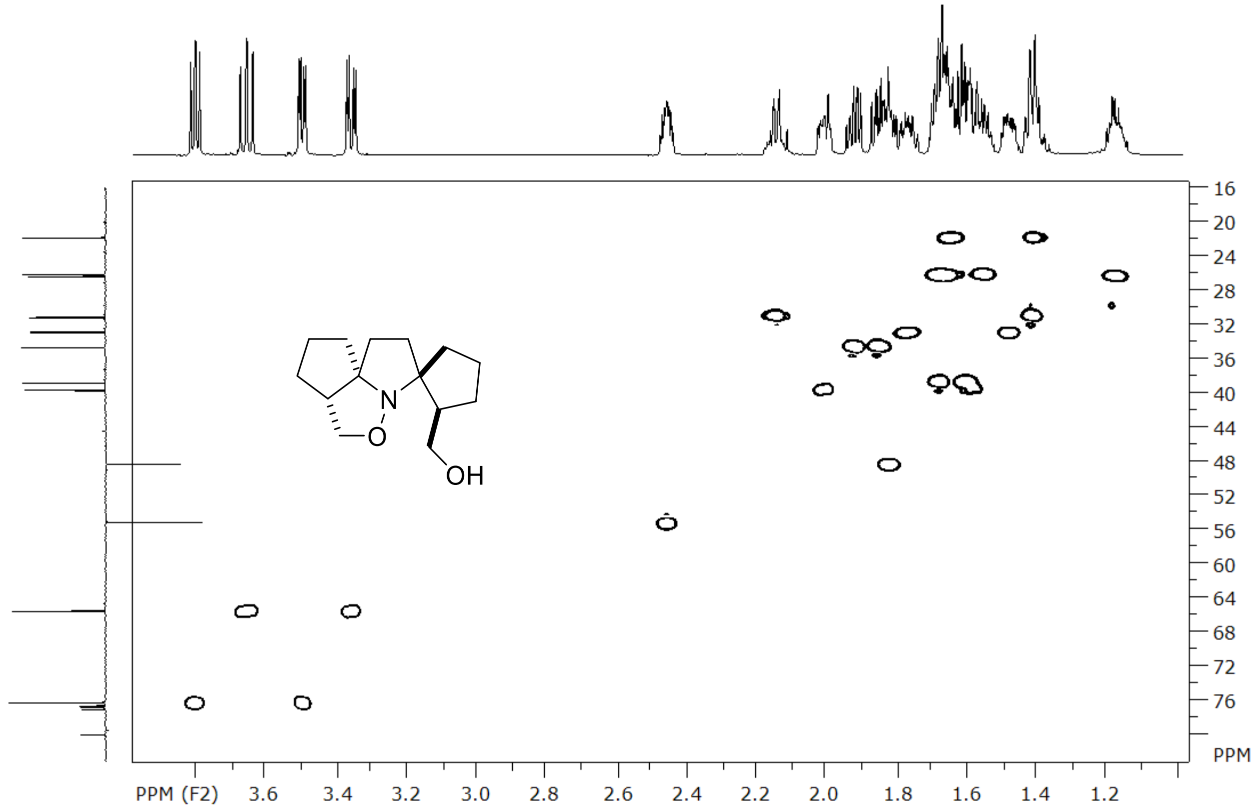

Fig. S66. ^{1}H-^{13}C HSQC NMR spectrum of (1*R*(*S*),2*R*(*S*),6a′*R*(*S*),9a′*R*(*S*)-Hexahydro-6′*H*-spiro[cyclopentan-1,3′-cyclopenta[*c*]pyrrolo[1,2-*b*]isoxazol]-2-ylmethanol (**13**) in $CDCl_3$.

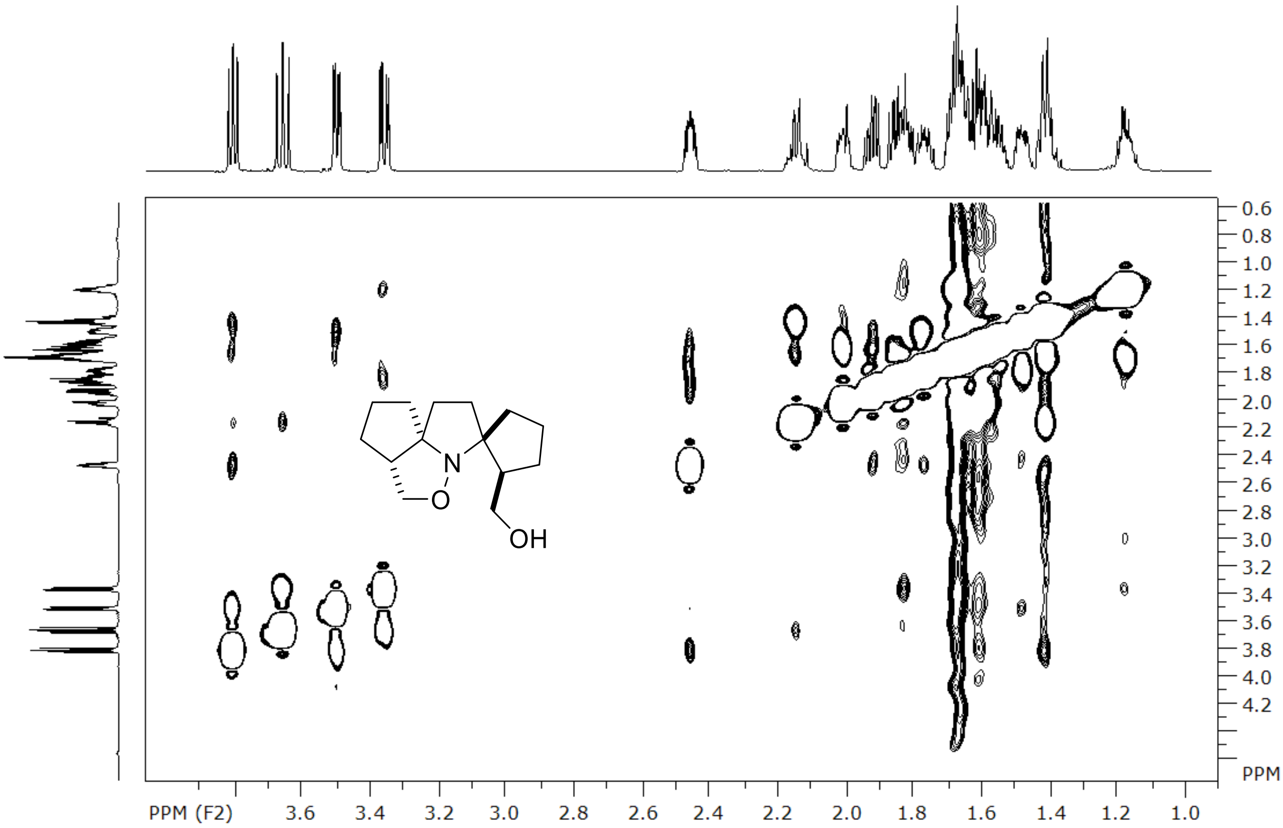

Fig. S67. ^{1}H-^{1}H NOESY NMR spectrum of (1*R*(*S*),2*R*(*S*),6a′*R*(*S*),9a′*R*(*S*)-Hexahydro-6′*H*-spiro[cyclopentan-1,3′-cyclopenta[*c*]pyrrolo[1,2-*b*]isoxazol]-2-ylmethanol (**13**) in $CDCl_3$.

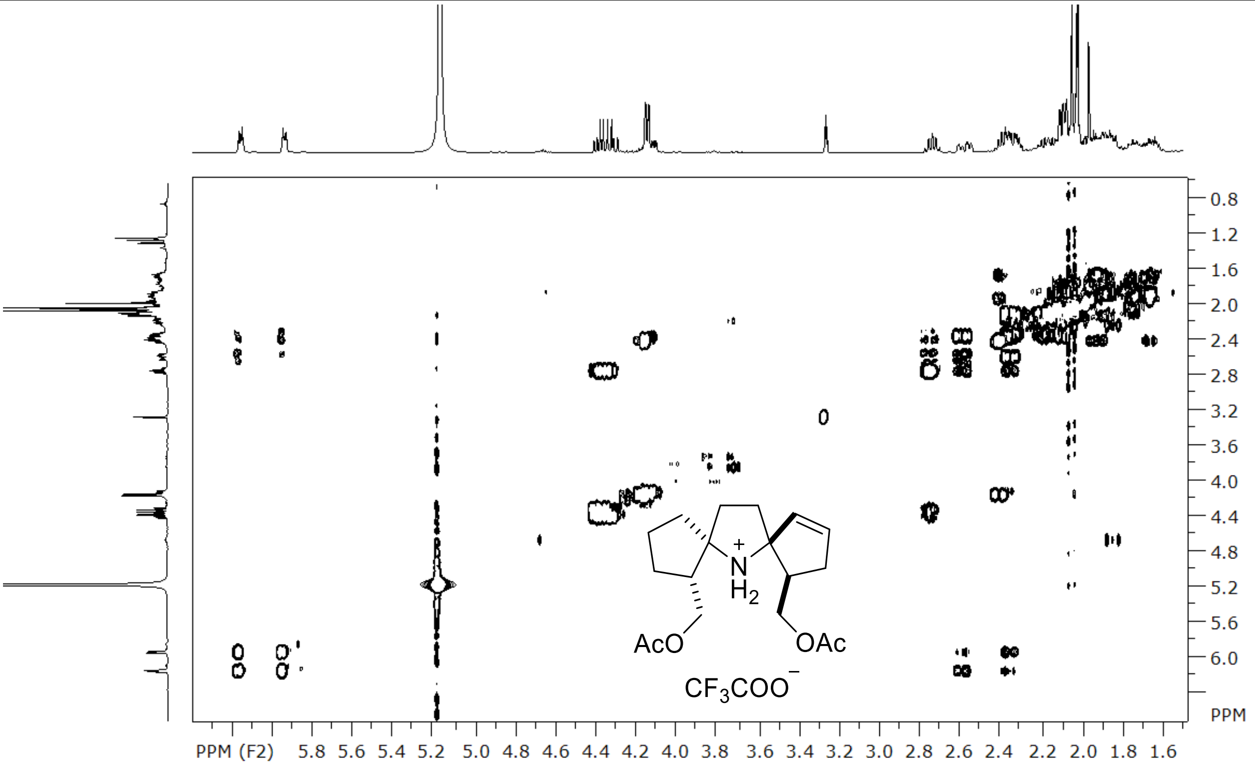

Fig. S68. ^1H-^1H COSY NMR spectrum of (4*R*(*S*),5*S*(*R*),7*R*(*S*),8*R*(*S*))-4,8-Bis[(acetyloxy)methyl]-6-azoniadispiro[4.1.4.2]tridec-1-ene trifluoroacetate (**19**) in CD_3OD+CF_3COOH.

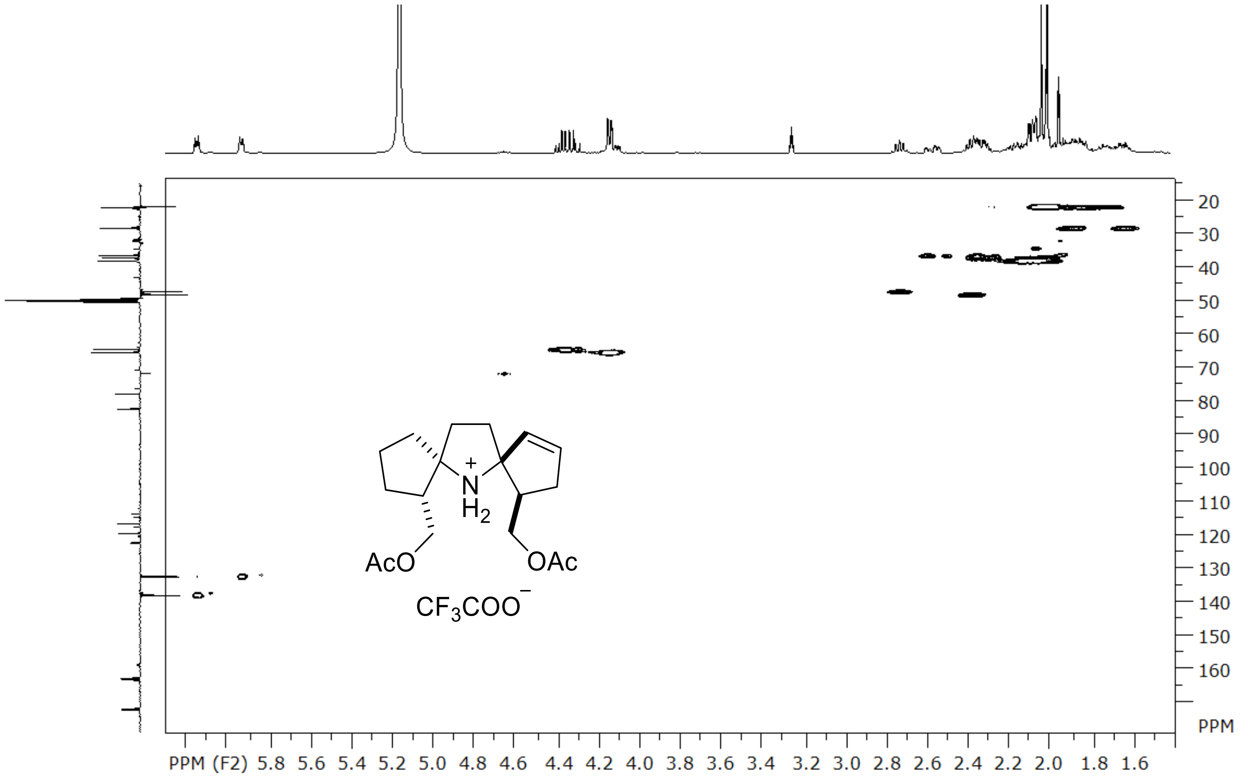

Fig. S69. ^1H-^{13}C HSQC NMR spectrum of (4*R*(*S*),5*S*(*R*),7*R*(*S*),8*R*(*S*))-4,8-Bis[(acetyloxy)methyl]-6-azoniadispiro[4.1.4.2]tridec-1-ene trifluoroacetate (**19**) in CD_3OD+CF_3COOH.

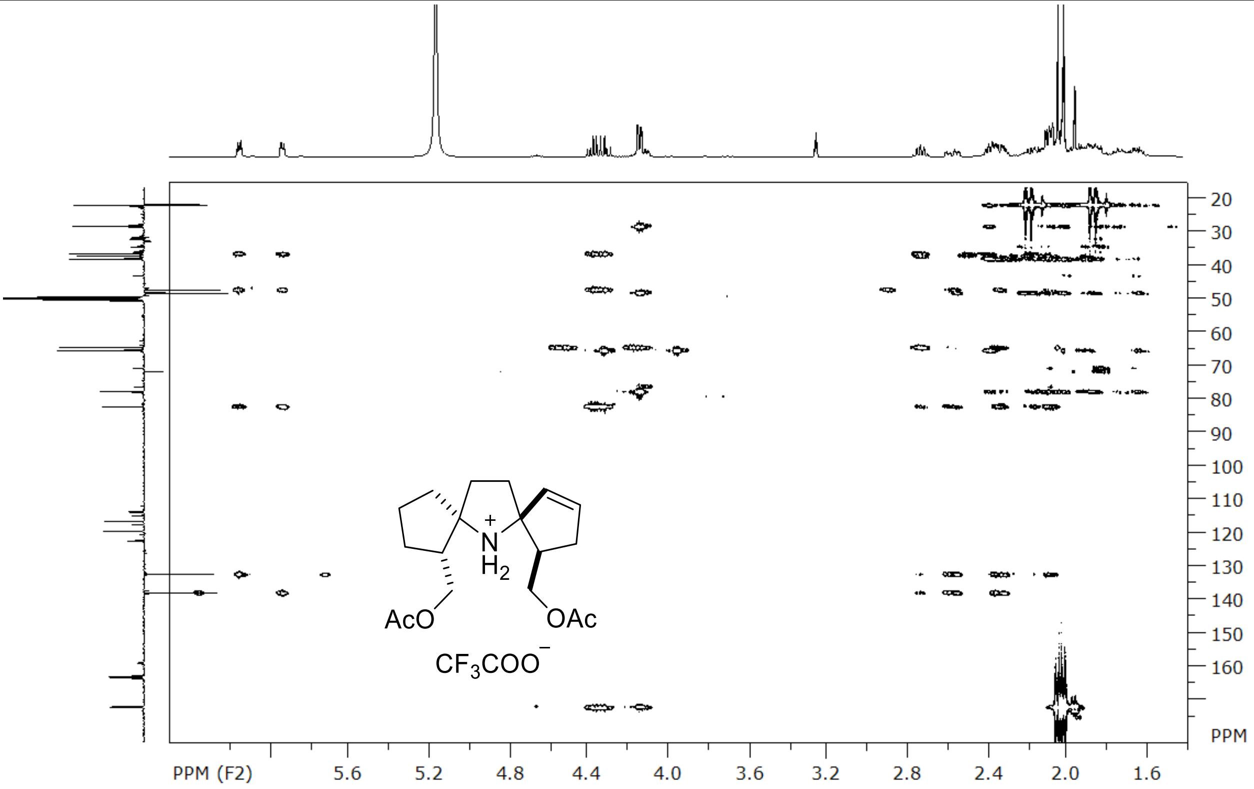

Fig. S70. ^{1}H-^{13}C HMBC NMR spectrum of (4*R*(*S*),5*S*(*R*),7*R*(*S*),8*R*(*S*))-4,8-Bis[(acetyloxy)methyl]-6-azoniadispiro[4.1.4.2]tridec-1-ene trifluoroacetate (**19**) in CD_3OD+CF_3COOH.

6. EPR measurements.

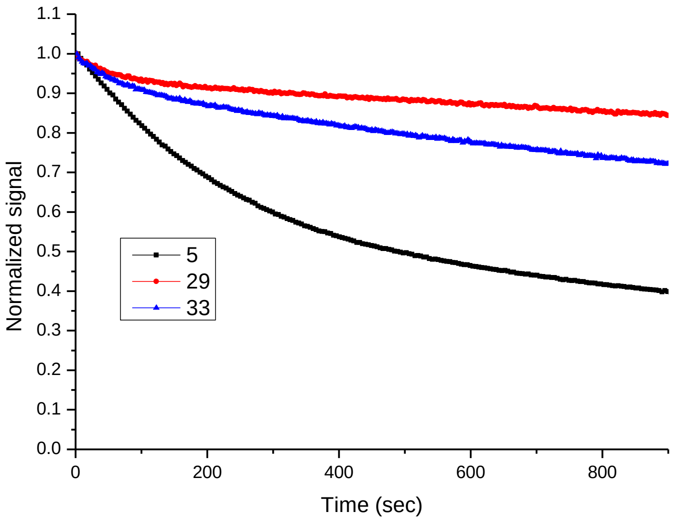

Fig. S71. The kinetics of decrease of EPR signal of nitroxides **5**, **29** and **33** in solution of ascorbic acid (100mM) and glutathione (5mM). These curves were fitted with exponential decay function to calculate the first order reaction rate constants, which were later used for calculation second order reaction rate constants.

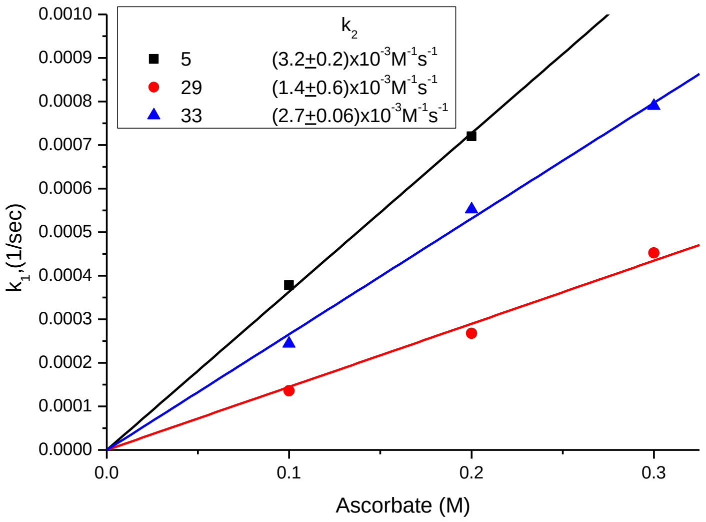

Fig. S72. The dependence of first order reaction rate constants versus ascorbic acid concentration of the radicals **5**, **29** and **33**. Data were fitted with linear dependence starting from zero value. The calculated slopes correspond to second order reaction rate constants.

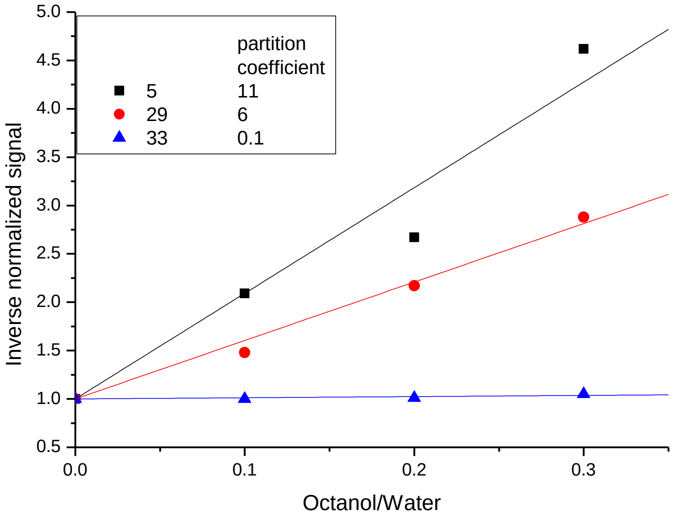

Fig. S73. Data for calculation of partition coefficients of the radicals **5**, **29** and **33**. The inverse value of the relative decrease of EPR signal (inverse normalized value) of the radical in the water were plotted versus octanol/water ratio. Data were fitted with linear dependence starting from one unit value. The absolute value of this slope corresponds to partition coefficient.

Table S1. Experimental details of X-ray diffraction experiments for compounds investigated.

	5	9	14	17
Crystal data				
Chemical formula	$C_{14}H_{24}NO_3$	$C_9H_{15}NO_2$	$C_{14}H_{25}NO_2$	$C_{18}H_{28}NO_5$
M_r	254.34	169.22	239.35	338.41
Crystal system, space group	Triclinic, $P\bar{1}$	Monoclinic, $P2_1/c$	Monoclinic, $C2/c$	Tetragonal, $P4_32_12$
Temperature (K)	299	296	296	296
a, *b*, *c* (Å)	6.5386(6), 18.1055(17), 19.912(2)	5.913(5), 11.952(11), 12.654(12)	25.596(3), 8.3639(7), 25.239(2)	6.9123(3), 6.9123(3), 37.383(2)
α, β, γ (°)	114.383(4), 91.155(4), 96.370(4)	90, 103.27(2), 90	90, 90.021(6), 90	90, 90, 90
V (Å^3)	2128.1(4)	870.4(13)	5403.2(9)	1786.2(2)
Z	6	4	16	4
F(000)	834	368	2112	732
μ (mm^{-1})	0.08	0.09	0.08	0.09
Crystal size (mm)	1.00 × 0.42 × 0.20	1.00 × 0.38 × 0.25	1.00 × 0.80 × 0.15	1.00 × 0.79 × 0.60
Data collection				
T_{min}, T_{max}	0.799, 0.862	0.742, 0.875	0.763, 0.862	0.798, 0.863
No. of measured, independent and observed [$I > 2\sigma(I)$] reflections	52841, 7588, 5736	6851, 1977, 1609	32116, 4879, 2916	30703, 3320, 3035
R_{int}	0.052	0.055	0.043	0.037
θ values (°)	θ_{max} = 25.1, θ_{min} = 1.1	θ_{max} = 27.5, θ_{min} = 2.4	θ_{max} = 25.3, θ_{min} = 2.3	θ_{max} = 35.0, θ_{min} = 3.0
Range of *h*, *k*, *l*	*h* = -7→7, *k* = -21→21, *l* = -23→23	*h* = -7→5, *k* = -14→15, *l* = -16→16	*h* = -30→30, *k* = -10→10, *l* = -30→30	*h* = -10→9, *k* = -11→11, *l* = -56→48
Refinement				
$R[F^2 > 2\sigma(F^2)]$, $wR(F^2)$, S	0.076, 0.241, 1.06	0.056, 0.184, 1.06	0.093, 0.294, 1.04	0.060, 0.164, 1.21
No. of reflections	7588	1977	4879	3320
No. of parameters	493	110	322	111
No. of restraints	12	0	3	0
$\Delta\rho_{max}$, $\Delta\rho_{min}$ (e Å^{-3})	0.45, -0.48	0.46, -0.34	0.47, -0.32	0.30, -0.20
Absolute structure parameter	–	–	–	0.52 (16)

	20	22	23a	26
Crystal data				
Chemical formula	$C_{14}H_{21}NO_4$	$C_{16}H_{24}NO_5$	$C_{14}H_{20}NO_5$	$C_{22}H_{28}N_5O_5$
M_r	267.32	310.36	282.31	442.49
Crystal system, space group	Triclinic, $P\bar{1}$	Orthorhombic, $P2_12_12$	Monoclinic, $P2_1/n$	Monoclinic, $C2/c$
Temperature (K)	296	296	296	299
a, *b*, *c* (Å)	8.4272(7), 8.9015(7), 9.3829(8)	10.9663(7), 11.8601(7), 6.3522(4)	9.4026(5), 11.9073(5), 12.6526(6)	12.6524(14), 10.5198(11), 16.8648(17)
α, β, γ (°)	83.367(3), 83.990(4), 66.215(3)	90, 90, 90	90, 98.191(2), 90	90, 103.860(4), 90
V (Å³)	638.45(9)	826.18(9)	1402.13(12)	2179.4(4)
Z	2	2	4	4
F(000)	288	334	604	940
μ (mm⁻¹)	0.10	0.09	0.10	0.10
Crystal size (mm)	0.48 × 0.12 × 0.09	0.54 × 0.20 × 0.10	0.63 × 0.50 × 0.44	0.60 × 0.19 × 0.16
Data collection				
T_{min}, T_{max}	0.816, 0.862	0.882, 0.958	0.869, 0.914	0.926, 0.958
No. of measured, independent and observed [$I > 2\sigma(I)$] reflections	15091, 2265, 1802	6026, 1455, 1303	22682, 3231, 2477	14914, 2385, 2035
R_{int}	0.041	0.058	0.037	0.040
θ values (°)	θ_{max} = 25.2, θ_{min} = 2.2	θ_{max} = 25.0, θ_{min} = 3.6	θ_{max} = 27.5, θ_{min} = 2.4	θ_{max} = 27.0, θ_{min} = 2.5
Range of *h*, *k*, *l*	*h* = -10→10, *k* = -10→10, *l* = -11→11	*h* = -12→13, *k* = -11→14, *l* = -7→7	*h* = -12→12, *k* = -15→15, *l* = -16→15	*h* = -15→16, *k* = -13→13, *l* = -21→15
Refinement				
$R[F^2 > 2\sigma(F^2)]$, $wR(F^2)$, *S*	0.075, 0.198, 1.04	0.082, 0.250, 1.02	0.060, 0.192, 1.01	0.041, 0.119, 1.07
No. of reflections	2265	1455	3231	2385
No. of parameters	174	139	209	165
No. of restraints	0	47	9	0
$\Delta\rho_{max}$, $\Delta\rho_{min}$ (e Å⁻³)	0.37, -0.29	0.54, -0.32	0.50, -0.41	0.22, -0.14
Absolute structure parameter	–	-0.4 (8)	–	–
